# The waning of the WIMP? A review of models, searches, and constraints

**DOI:** 10.1140/epjc/s10052-018-5662-y

**Published:** 2018-03-10

**Authors:** Giorgio Arcadi, Maíra Dutra, Pradipta Ghosh, Manfred Lindner, Yann Mambrini, Mathias Pierre, Stefano Profumo, Farinaldo S. Queiroz

**Affiliations:** 1 Max Planck Institut für Kernphysik, Saupfercheckweg 1, 69117 Heidelberg, Germany; 2 Laboratoire de Physique Théorique, CNRS, Univ. Paris-Sud, Université Paris-Saclay, 91405 Orsay, France; 3 Centre de Physique Théorique, Ecole Polytechnique, CNRS, Université Paris-Saclay, 91128 Palaiseau Cedex, France; 4 Department of Physics, University of California, Santa Cruz, 1156 High St, Santa Cruz, CA 95060 USA; 5 Santa Cruz Institute for Particle Physics, Santa Cruz, 1156 High St, Santa Cruz, CA 95060 USA

## Abstract

Weakly Interacting Massive Particles (WIMPs) are among the best-motivated dark matter candidates. No conclusive signal, despite an extensive search program that combines, often in a complementary way, direct, indirect, and collider probes, has been detected so far. This situation might change in near future due to the advent of one/multi-TON Direct Detection experiments. We thus, find it timely to provide a review of the WIMP paradigm with focus on a few models which can be probed at best by these facilities. Collider and Indirect Detection, nevertheless, will not be neglected when they represent a complementary probe.

## Introduction

A combination of cosmological observations including (between others) studies of the cosmic microwave background (CMB), distant supernovae, large samples of galaxy clusters, baryon acoustic oscillation measurements has firmly established a standard cosmological model where the Dark Matter (DM), a new yet-to-be discovered form of matter, accounts for about 85% of the matter content of the Universe, and about 27% of the global energy budget [1]. Cold Dark Matter (CDM) is a key ingredient to successfully explain the formation of large-scale structure, producing theoretical predictions in striking agreement with observations [2, 3].

**Fig. 1:**
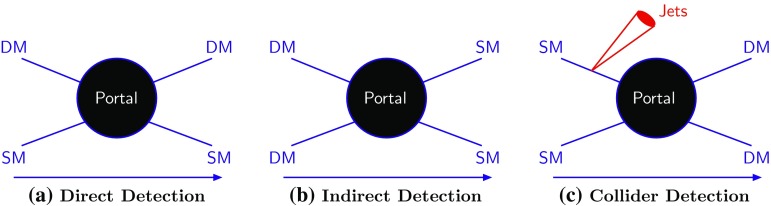
Illustration of the DM interactions with the SM particles. As of today we have no knowledge about how such interaction occurs. Thus, it is literally a black box

Little is, however, as of yet known about DM as a particle; any candidate for (most of) the DM must nevertheless be consistent with the following five observationally-motivated constraints:

(i) The relic abundance of DM needs to account for the observed CDM abundance;
(ii) the DM particle should be non-relativistic at matter-radiation equality to form structures in the early Universe in agreement with the observation. As a result, if the DM was produced as a thermal relic in the early Universe, its mass cannot be arbitrarily light. Specifically, cosmological simulations rule out DM masses below a few keV [4–6].
(iii) The DM should be electromagnetically neutral, as a result of null searches for stable charged particles [7, 8] as well as Direct Detection (DD) experiments, which we will review subsequently.
(iv) The DM particle must be cosmologically stable since its presence is ascertained today, implying that its lifetime is larger than the age of the Universe. Under certain assumptions, much stronger limits are applicable conservatively requiring a lifetime order of magnitude larger can be derived [9–16].
(v) Cluster collisions, such as the Bullet Cluster [17], constrain the level of self-interactions that DM particles can have (see however, Refs. [18, 19] for alternative scenarios).

Within the generous parameter space outlined by the observational requirements listed above, we will argue below that the paradigm of WIMPs [20] is one of the most compelling options for DM as a particle. As such, it has undergone very intricate and effective experimental scrutiny. A very schematic representation of the main DM detection strategies is shown in Fig. 1.

In this work we will attempt to give an up-to-date state of the art of ongoing WIMP searches and possible future prospects.

The extreme broadness of the topic makes, however, very difficult to satisfactory cover all the different DM search strategies. Consequently we will mainly focus on DM DD, motivated by the advent of highly sensitive one- and multi-Ton detectors. We will then investigate, a selection of simple, but well motivated, WIMP models whether and to which extent the WIMP paradigm can be tested through this kind of search strategy.

Even within the WIMP framework, other DM search strategies like collider searches and Indirect Detection (ID) provide a complementary and essential contribution. This complementarity will be highlighted in some relevant cases of study.

Before discussing the main topic, we will anyway provide in the next sections a general and pedagogical introduction to the WIMP paradigm for the generation of the cosmological abundance of the DM relic density and to the three main categories of DM searches: DD, ID and collider searches.

## The WIMP paradigm

The paradigm of thermal decoupling, based upon applications to cosmology of statistical mechanics and particle and nuclear physics, is enormously successful at making detailed predictions for observables in the early Universe, including the abundances of light elements and the CMB [21]. It is somewhat natural to invoke a similar paradigm to infer the abundance of DM as a thermal relic from the early Universe uniquely from the underlying DM particle properties.

Assuming there exist interactions between a cosmologically stable particle $\chi$ – the (generic) DM – with Standard Model (SM) particles, sizable enough so that for a high enough temperature *T* the DM is in thermal equilibrium with the primordial thermal bath, the cosmological evolution of the DM particle can be traced through the following Boltzmann equation:

1 $$\begin{aligned} \frac{dn_{\chi }}{dt} +3 H(T) n_\chi = -\langle \sigma v \rangle (n_{\chi }^2 -n_{\chi , eq}^2), \end{aligned}$$

describing the DM number density $n_\chi$, in turn defined as:

2 $$\begin{aligned} n_\chi (T)=g_\chi \int \frac{d^3 p}{{\left( 2 \pi \right) }^3}f_\chi (p,T), \end{aligned}$$

with $f_\chi$ being the DM distribution function. $g_{\chi }$ is the number of internal degrees of freedom of the DM particle.

The quantity $\langle \sigma v \rangle$, dependent on temperature *T*, is the thermally averaged pair annihilation cross-section associated to the process $\chi \chi \rightarrow$ pair of SM particles while *H*(*T*) is the Hubble rate. Assuming a standard cosmological evolution for the Early Universe, the whole process of DM production occurs while the Universe is dominated by the radiation energy density so that the Hubble expansion parameter is given by:

3 $$\begin{aligned} H(T)=\sqrt{\frac{8\pi }{3}}\frac{1}{M_\mathrm{Pl}}\sqrt{\rho _r (T)},\quad \rho _r(T)=\frac{\pi ^2}{30}g_\mathrm{eff}(T) T^4, \end{aligned}$$

where $g_\mathrm{eff}(T)$ is the effective number of relativistic degrees of freedom at the temperature *T* and $M_\mathrm{Pl}=1.22 \times 10^{19}\,\text{ GeV }$ is the Planck mass. Moreover, $n_{\chi ,eq}$ is the equilibrium number density obtained from Eq. (2) by replacing $f_\chi$ with the equilibrium distribution function (by convenience one typically adopts the Maxwell–Boltzmann distribution):

4 $$\begin{aligned} n_{\chi , eq}= g_{\chi } \frac{m_{\chi }^2 T}{2\pi } K_2\left( \frac{m_\chi }{T}\right) , \end{aligned}$$

where $m_{\chi }$ is the DM mass while $K_i$ is the modified Bessel function of order ‘*i*’. Equation (1) is more easily handled by adopting as dependent variable the ‘yield’ or comoving number density:

5 $$\begin{aligned}&Y_{\chi }=\frac{n_\chi }{s}\quad \mathrm{with}{:} \end{aligned}$$

6 $$\begin{aligned}&s=\frac{2\pi ^2}{45}h_\mathrm{eff}(T)T^3, \end{aligned}$$

being the entropy density ($h_\mathrm{eff}(T)$ is the effective number of entropy degrees of freedom at the temperature *T*), so that it is possible to get rid of the term dependent on the Hubble expansion rate on the left-hand side of Eq. (1), giving:

7 $$\begin{aligned} \frac{dY_\chi }{dt}=\frac{ds}{dt}\frac{\langle \sigma v \rangle }{3H}Y_\chi ^2 \left( 1-\frac{Y_{\chi ,eq}^2}{Y_\chi ^2}\right) . \end{aligned}$$

To obtain the last equation we have used the entropy conservation relation $\frac{ds}{dt}=-3Hs$. Qualitatively, Eq. (7), describes the following picture: If DM interactions are enough efficient, as in the case of WIMPs, at early times the annihilation rate $\varGamma _\mathrm{ann}=\langle \sigma v \rangle Y_\chi s$ exceeds the Hubble expansion rate and Eq. (7) is solved for $Y_\chi = Y_{\chi ,eq}$, meaning that the DM is in thermal equilibrium with the primordial thermal bath. At later times, when the temperature eventually drops below the DM mass, the DM yield becomes Boltzmann suppressed, $Y_{\chi , eq}\propto \exp (-m_\chi /T)$, so that the annihilation rate falls below the Hubble expansion rate leading to the thermal freeze-out of this “cold” relic, i.e., thereafter $Y_\chi$ is approximately constant with time.^1^ Equation (7) can be solved by adopting the temperature *T*^2^ of the thermal bath or $x=m_\chi /T$ as independent variable. A good approximate solution is represented by the following semi-analytical expression [23]:

9 $$\begin{aligned} Y(T_0)\equiv Y_0 \simeq \sqrt{\frac{\pi }{45}}M_\mathrm{Pl}{\left[ \int _{T_0}^{T_f} g_{*}^{1/2} \langle \sigma v \rangle dT \right] }^{-1}, \end{aligned}$$

where:

10 $$\begin{aligned} g_{*}^{1/2}=\frac{h_\mathrm{eff}}{g^{1/2}_\mathrm{eff}}\left( 1+\frac{1}{3}\frac{T}{h_\mathrm{eff}}\frac{dh_\mathrm{eff}}{dT}\right) , \end{aligned}$$

with $T_0$ as the present time temperature while $T_f$ represents the freeze-out temperature which can be determined by solving the equation:

11 $$\begin{aligned} \sqrt{\frac{\pi }{45}}M_\mathrm{Pl} \frac{g_{*}^{1/2} m_\chi }{x^2}\langle \sigma v \rangle Y_{\chi , eq} \delta (\delta +2)=-\frac{d \log Y_{\chi , eq}}{dx}, \end{aligned}$$

where $\delta =(Y_\chi -Y_{\chi , eq})/Y_{\chi , eq}$ is conventionally set to 1.5 while $x=m_\chi /T$.

The DM relic abundance is usually expressed in terms of the parameter $\varOmega _\mathrm{DM}h^2$ where $h \sim 0.7$ is the value Hubble expansion rate at present times in units of 100 (km/s)/Mpc while $\varOmega _\mathrm{DM}$ represents the ratio between the DM energy density $\rho _\mathrm{DM}$ and the so called critical energy density $\rho _\mathrm{cr}$, namely:

12 $$\begin{aligned} \varOmega _\mathrm{DM}= & {} \rho _\mathrm{DM}/\rho _\mathrm{cr}(T_0),\quad \rho _\mathrm{DM}=m_\chi s_0 Y_0, \nonumber \\ \rho _\mathrm{cr}(T)= & {} 3 H(T)^2 M_\mathrm{PL}^2/8 \pi ,\quad \rho _\mathrm{cr}(T_0) \simeq 10^{-5}~{\mathrm {GeV}}~\hbox {cm}^{-3},\nonumber \\ \end{aligned}$$

where $s_0 =s(T_0)$ is the entropy density at present times.

By combining the expressions above, the DM relic density can be numerically estimated as:

13 $$\begin{aligned} \varOmega _\mathrm{DM}h^2 \approx 8.76 \times 10^{-11}\, {\text{ GeV }}^{-2} {\left[ \int _{T_0}^{T_f} g_{*}^{1/2} \langle \sigma v \rangle \frac{dT}{m_\chi } \right] }^{-1}. \end{aligned}$$

**Fig. 2:**
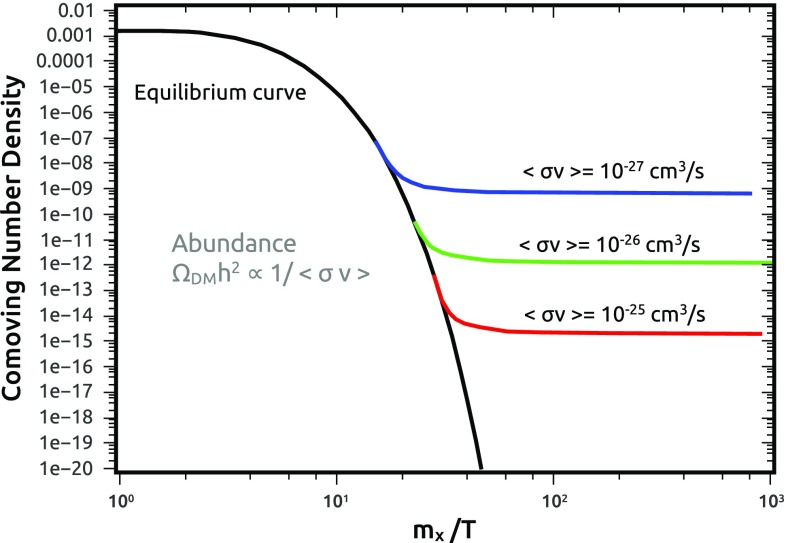
Comoving number density evolution as a function of the ratio $m_{\chi }/T$ in the context of the thermal freeze-out. Notice that the size of the annihilation cross-section determines the DM abundance since $\varOmega _\mathrm{DM} h^2 \propto 1/ \langle \sigma v \rangle$

The behavior of the solution of the Boltzmann equation is illustrated in Fig. 2. As expected, the DM relic density is basically set by the inverse value of the thermally averaged cross-section (calculated at the freeze-out temperature), with a logarithmic dependence on $m_\chi$. It can be straightforwardly verified that the experimental determination of $\varOmega _\mathrm{DM}h^2 \approx 0.12$ [1] is matched by a value of the cross-section of the order of $10^{-9}\, {\text{ GeV }}^{-2}$ corresponding to $\langle \sigma v \rangle \sim 10^{-26}\,{\text{ cm } }^3\,{\text{ s }}^{-1}$.

The WIMP paradigm hence reduces, under the hypothesis of standard cosmological evolution of the Universe, the solution of the DM problem to the determination of a single particle physics input, i.e., the thermally averaged pair annihilation cross-section of the DM.

Its formal definition reads [23]:^3^

14 $$\begin{aligned} \langle \sigma v \rangle= & {} \frac{{K_2\left( \frac{m_\chi }{T}\right) }^{-2}}{8 m_\chi ^4 T}\int _{4 m_\chi ^2}^{\infty } ds~ \nonumber \\&\times \,\sigma (s) \sqrt{s} \left( s-4 m_\chi ^2\right) K_1\left( \frac{\sqrt{s}}{T}\right) , \end{aligned}$$

where $\sigma (s)$ is the DM annihilation cross-section, as computed using the conventional field theory techniques.

Since WIMPs freeze out in the non-relativistic regime, and thus, $v\ll c$ (where *v* is the relative velocity of the two annihilating WIMPs), a useful approximation consists of a velocity expansion (given in the Appendix) $\langle \sigma v \rangle \simeq a + b v^2$. The velocity expansion is, however, not valid in some relevant cases, like for example annihilations through the resonant exchange of an s-channel mediator [25]. For this reason, all the numerical results presented in this work will rely on the full numerical determination of $\langle \sigma v \rangle$, as given in Eq. (14) and on the solution of the DM Boltzmann equation, as provided by the numerical package micrOMEGAs [26–28].

The WIMP “miracle” is the observation that the cosmologically favored value of the DM pair annihilation cross-section is met by a DM featuring electroweak (EW) interactions, i.e., with cross-section scaling as $\langle \sigma v \rangle \sim g^4/m_\chi ^2$, with *g* being a coupling of the order of the EW gauge couplings and mass in the $O(100-1000)\,\text{ GeV }$ range and with typical freeze-out temperature $T_{f}\sim \frac{m_\chi }{20}$. It is important to realize that this coincidence is a statement about cross-sections (and, weakly, masses), and thus, is not unique to the weak scale and weak interactions. In large fraction of this work we will for example consider the scenario in which the DM is a SM singlet interacting (mostly) with the SM fermions through a mediator field. In this case a typically expected scaling, in the case when the DM is lighter than the mediator, for the cross-section would be $\langle \sigma v \rangle \sim \lambda _\chi ^2 \lambda _f^2 \frac{m_\chi ^2}{m_\mathrm{med}^4}$ where $m_\mathrm{med}$ is the mass of the mediator field while $\lambda _\chi$ and $\lambda _f$ are, respectively, the couplings of the DM with the mediator and of the latter with the SM fields. A very simple numerical estimate hence, gives

15 $$\begin{aligned} \langle \sigma v \rangle \sim 10^{-25} {\text{ cm }}^3 {\text{ s }}^{-1} {\left( \frac{m_\chi }{100\,\text{ GeV }} \right) }^2 {\left( \frac{1\,\text{ TeV }}{m_\mathrm{med}}\right) }^4 \lambda _\chi ^2 \lambda _f^2. \end{aligned}$$

This shows that also in this setup, the cosmological relic density can be obtained for EW scale masses of the DM and of the mediator field, provided a suitable assignation of the couplings $\lambda _\chi ,\lambda _f$. It is remarkable to notice that the WIMP paradigm provides, independent of theoretical reasons such as naturalness and the hierarchy problem, a plausible argument to expect new physics at and around the EW scale.

As a result, concrete realizations of WIMP models have been developed in different Beyond the Standard Model (BSM) frameworks, accessible to several different search strategies, as reviewed in the next sections.

## Direct detection

Astrophysical and gravitational evidences indicates the existence of DM halos, surrounding all the visible structures like galaxies and the cluster of galaxies. The halos are formed by DM particles described by a time dependent velocity distribution. DD experiments aim at detecting DM, through scattering off nuclei, belonging to the halo surrounding our galaxy, flowing through the Earth. Several experiments have played an important role in this direction [29–59]. In this section we focus on DD experiments looking for WIMPs scattering, but there are important searches stemming from Neutrino telescopes by measuring the neutrino flux from the Sun [60–63].

Direct DM detection seeks to measure the nuclear recoil imparted by the scattering of a WIMP particle. The WIMP-nuclei differential scattering rate can be written as,

16 $$\begin{aligned} \frac{dR(E,t)}{dE} = \frac{N_T \rho _{\chi } }{m_{\chi }\, m_A} \int _{v_\mathrm{min}}^{v_\mathrm{esc}} v f_E(\mathbf {v},t) \frac{d\sigma (v,E)}{dE} d^3\mathbf {v}, \end{aligned}$$

**Fig. 3:**
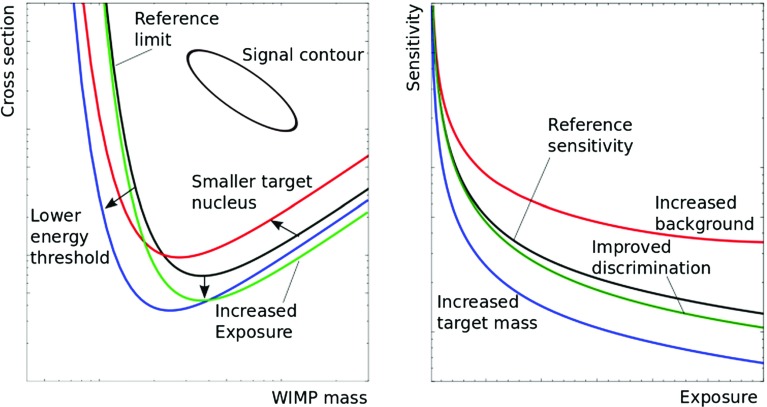
*Left* Illustrative impact of the energy threshold, exposure and target nucleus. *Right* Impact of background and exposure on the sensitivity. These plots are taken from Ref. [73]

where *E* is the recoil energy associated to the scattering events, $N_T$ is the number of target nuclei per kilogram of the detector, $m_{\chi }$ is the DM mass, $\rho _{\chi }$ is the local DM density ($\rho _{\chi } = 0.3\, \mathrm {GeV}/\mathrm {cm}^3$) [64–68], $\mathbf {v}$ is the velocity of the DM particle relative to the Earth, $f_E(\mathbf {v},t)$ is the distribution of velocities of the WIMP in the frame of the Earth,^4^ $v_\mathrm{min}=\sqrt{m_N E/(2\mu ^2)}$ is the minimum WIMP speed required to produce a detectable event at energy *E*, $v_\mathrm{esc}$ is the escape velocity i.e., the velocity for which the WIMP are no longer gravitationally bounded to the Milky Way. $\mu = m_{\chi } m_N/ (m_{\chi } +\, m_N)$ is the WIMP-nucleus reduced mass ($m_N$ is the nucleus mass), $d\sigma$/*dE*(*v*, *E*) is the differential cross-section for the WIMP-nucleus scattering as follows,

17 $$\begin{aligned} \frac{d\sigma }{dE} = \frac{m_N}{2\mu ^2 v^2} (\sigma ^{SI} F^2(q) + \sigma ^{SD} S(q)), \end{aligned}$$

with *q* being the momentum transfer. $F^2(q)$ and *S*(*q*) are the Spin-Independent (SI) and Spin-Dependent (SD) form factors, as described e.g., in Refs. [69–72].

After measuring the scattering rate, the next and fundamental task is to discriminate the signal from the background. This is done by using the detector response to electron and nuclear scattering, which might vary from one experiment to another. For instance, in Germanium detectors ionization yield is used to discriminate signal from background, whereas in experiments that use Xenon, the ionization/scintillation ratio is the discriminating variable. What determines an experiment’s sensitivity to a WIMP signal is a combination of:

1. *Energy threshold*: drives the sensitivity to low WIMP masses, and consequently the sharpening of the DD limits on the scattering cross-section at low masses as shown in Fig. 3;
2. *Control over the background and exposure:* determine the overall sensitivity of the experiment pushing the limits to lower scattering cross-sections assuming that they are statistically dominated;
3. *Target:* has an impact on the experimental sensitivity to low and heavy WIMP masses, as well as on the capability to probe SD scatterings.

All these facts are illustrated in Fig. 3 where we plotted the impact of exposure, energy threshold and mass of the nucleus target on DD experiments sensitivity for WIMP-nucleon scatterings. Figure 3 is described as follows: *Left panel:* (i) Comparing the solid black and blue curves at low WIMP mass one can see that the energy threshold determines the smallest WIMP mass accessible to a given DD experiment. The blue line refers to a DD experiment with lower energy threshold; (ii) Notice that stronger bounds on the scattering cross-section are possible with larger exposure as represented in the solid green line; (iii) The target nucleus can influence the WIMP mass where the strongest limit on the scattering cross-section lies at and also the sensitivity to lower and larger WIMP masses. This is visible by comparing the red and black lines. *Right panel:* (i) Comparing the black and blue curves we can see the importance of increasing exposure; (ii) Red and green curves exhibit the impact of background discriminations.

It is important to highlight that from going to the measured scattering rate in Eq. (16) to the derivation of a limit on the WIMP-nucleon scattering cross-section as a function of the WIMP mass, there are some assumptions that have to be made about the velocity distribution, nuclear form factor, type of WIMP-nucleon scattering, and local DM density that suffers from large uncertainties [74–76]. In particular, the common assumptions are that there is a smooth halo of DM particles in our galaxy well described by a Maxwellian velocity distribution [77–79], that the nucleus can be treated as a hard sphere as indicated by the Helm form factor [70], and that the WIMP-nucleon scattering is elastic. Our results rely on the same set of assumptions throughout this manuscript (see Refs. [80–84] for further discussions on these topics). Interestingly, if the uncertainties present in the astrophysical input are under control and precise measurements on the scattering cross-section can be realized, then one might even determine the nature of DM using DD experiments alone [85].

In summary, the present measurement of the scattering rate has not yet observed any excess over the background, which after some assumptions, translates into limits on the WIMP-nucleon scattering cross-section as a function of the DM mass. In this work we will be using the following limits and projections:

- *Current spin-independent limit:*

We adopt the latest result from XENON1T [86], based on 34.2 live days of exposure and a fiducial mass of $1042 \pm 12\,\text{ kg }$. The maximal sensitivity corresponds to a DM mass of $35\,\text{ GeV }$ for which SI scattering cross-sections above $7.7\times 10^{-47}\,{\text{ cm }}^2$ are excluded at $90\%$ confidence level. Despite of the limited exposure time, this result improves the previous limits provided by LUX, with strongest exclusion (90% C.L) of $2.2\times 10^{-46}\,{\mathrm{cm}}^2$ at the value 50 GeV [57] of the WIMP mass based on $3.35\times 10^4$ kg  day exposure and, PandaX [87], whose maximal sensitivity $2.5\times 10^{-46}\,{\text{ cm }}^2$ based on $3.3 \times 10^4$ kg day exposure is reached for WIMP mass of 40 GeV, hence showing the potential capability of the detector in probing the WIMP parameter space.

- *Current spin-dependent limit:*

We adopt the latest results by LUX considering the full exposure time [88]. For the SD cross-section on neutrons, a limit as strong as $1.6 \times 10^{-41}\,{\mathrm{cm}}^2$ for a WIMP of mass 35 GeV is obtained. Also in this case similar limits have been reported by the PandaX collaboration, based on $3.3 \times 10^4$ kg day of exposure. Hopefully PandaX will continue to run and improve their sensitivity and possibly unearth a DM signal.

- *Projected spin-independent limit:*

We adopt the projected XENON1T SI limits outlined in Ref. [89] assuming a 2 years exposure and the LZ collaboration referred as baseline in Ref. [90].

- *Projected spin-dependent limit:*

In the lack of published projections for SD scattering we simply rescaled the current SD limits taking into account the planned exposure. In practice, we derived the scaling factor between latest LUX SI limit and the LZ projection, and then applied this same scaling factor to derive the LZ/XENON1T projection for SD scattering. In the light of no large background, the limits will roughly be improved simply by exposure, justifying our method.

**Fig. 4:**
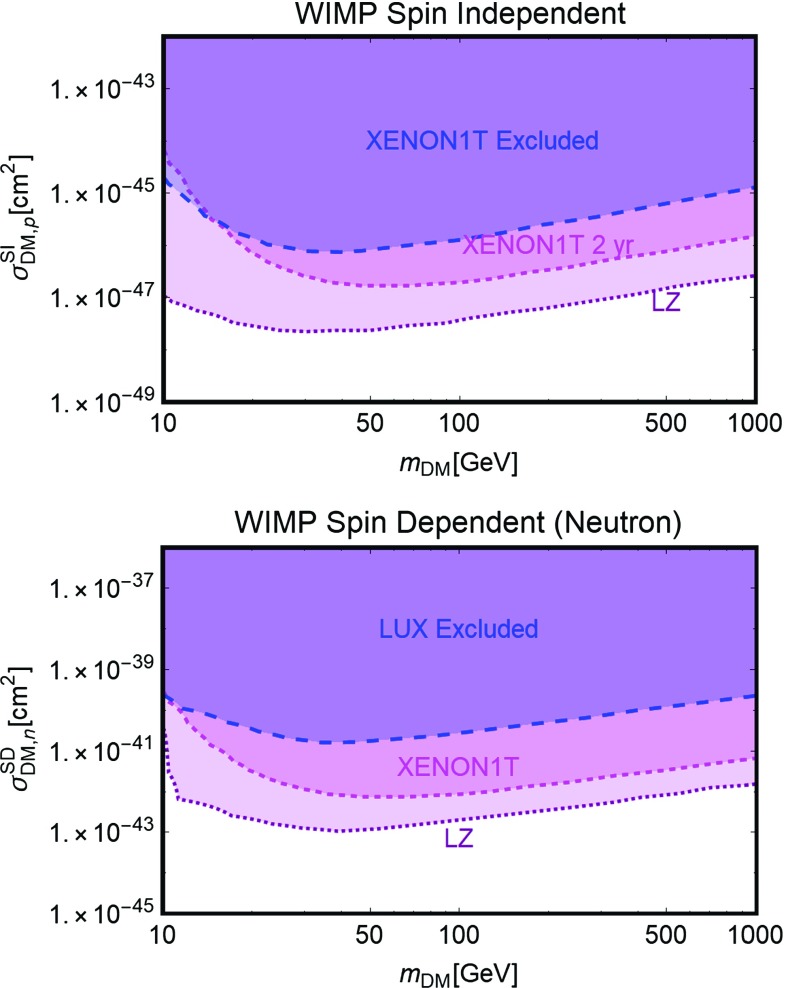
Limits (by LUX) and projected sensitivities (by XENON1T and LZ) adopted in this study for SI (top panel) and SD (bottom panel) interactions in the bi-dimensional plane (DM mass, cross-section). For SI interactions we have reported, as usual, the scattering cross-section of the DM on protons; for SD we have, instead, considered scattering on neutrons since we are considering “Xenon based” detectors. The blue region are, respectively, currently excluded by LUX while the magenta/purple regions will be excluded in case of absence of signals at XENON1T/LZ

The limits (current and projected) which will be adopted throughout all this work are illustrated in Fig. 4. The two panels report, in a bi-dimensional plane of DM mass and scattering cross-section, the region which are/will be excluded, under the null result, by the concerned experiments. As evident that the experimental sensitivity appears to be very different between SI and SD interactions. This is due to the fact that the SI cross-section of the DM on a nucleus originates from a coherent sum of the contributions from the interactions of the DM with the single nucleons. Targets, like Xenon, with atomic number of the order of 100 feature then an enhanced sensitivity to SI interactions. The case of SD interactions is, instead very different, since the contribution to the different nucleons tend to interfere destructively, so that a sizable cross-section is obtained only for targets with an unpaired nucleon.

As customary, we have expressed the limits on SI interactions in terms of the scattering cross-section of the DM on protons. For SD interactions we have instead reported the scattering cross-section on neutron since Xenon targets, characterizing all the three considered experiments, are mostly sensitive to this process having Xenon isotopes with unpaired neutrons.

In most of the models considered in this work the DM candidate will have unsuppressed (i.e., independent from the DM velocity or the momentum transfer) SI interactions. Given the stronger sensitivity, we will consider just the limits from SI interactions in presenting our results while report the ones from SD interactions only when relevant.

## Indirect detection

ID of the DM relies on the detection of the byproducts of WIMPs annihilations over the expected background at galactic or extra-galactic scales, using Earth based telescopes such as HESS and CTA, or satellites such as AMS and Fermi-LAT [91–105]^5^

In this regard, the search for gamma-rays and cosmic-rays and neutrinos offer an exciting possibility of DM detection. Here we will focus on gamma-rays. The gamma-ray flux from WIMP annihilation is proportional to:

- The number density squared of particles, i.e., $n_{\chi }^2=\rho ^2/m_{\chi }^2$;
- The WIMP annihilation cross-section today, $\sigma$;
- The mean WIMP velocity *v*;
- Volume of the sky observed within a solid angle $\varOmega$;
- Number of gamma-rays produced per annihilation at a given energy and for a given annihilation final state, also known as the energy spectrum (*dN* / *dE*).

In summary, it is found to be:18Therefore, the differential gamma-ray flux in Eq. (18) is determined with the three different inputs, DM annihilation cross-section, energy spectrum, the integral over the line of sight (l.o.s) of the DM density distribution, as represented above. The DM annihilation cross-section along with the energy spectrum capture the particle physics input of the model, whereas the DM distribution accounts for the astrophysical input. At the end of the day, the most important information needed on the particle physics side is to know whether the DM annihilation cross-section depends on the relative velocity of the WIMP. If it does, then the annihilation cross-section today is bound to be suppressed, rendering ID probes sub-dominant or irrelevant. The energy spectrum plays also an important role, because different final states produce distinct gamma-ray yields. Some final states produce hard gamma-rays at high energies, while others at lower energies. For this reason, the flux of gamma-ray due to DM annihilation directly depends on the final state annihilation. The DM density profile which is not so well-known serves as a normalization quantity, since it does not feature any energy dependence, but gives an overall constant for a given target region.

In Eq. (18) the DM density is integrated over the l.o.s from the observer to the source. The DM density is not tightly constrained, and several DM density profiles have been considered in the literature leading to either spike or core DM densities toward the center of galaxies [115–121]. In this work we adopt the Navarro–Frenk–White (NFW) profile [117] which reads,

19 $$\begin{aligned} \rho (r) = \frac{r_s}{r} \frac{\rho _s}{[1+ r/r_s]^2}, \end{aligned}$$

where $r_s=24.42$ kpc is the scale radius of the halo, as used by Fermi-LAT collaboration in Ref. [101], and $\rho _s=0.184$ is a normalization constant to guarantee that the DM density at the location of the Sun is $0.3\mathrm{~GeV/cm^3}$. It is important to emphasize that the NFW profile is known as steep profile, since it leads to a large DM density toward the inner regions of the galactic center. Therefore, it is not a conservative choice [122]. Different profiles, core-like, can significantly weaken limits from ID probes.

From Eq. (18) it is clear that ID probes complementary properties of the DM particles. It is sensitive to how the DM is distributed, to the annihilation cross-section today, which might be different than the annihilation cross-section relevant for the relic density, and to the WIMP mass. Therefore, after measuring the flux of gamma-rays from a given source, we compare it with the background expectations. If no excess is observed, we can choose a DM density profile and select an annihilation final state needed for *dN* / *dE*, and then derive a limit on the ratio $\sigma v/m_\chi ^2$ according to Eq. (18). This is the basic idea behind experimental limits. Although, more sophisticated statistical methods have been conducted such as likelihood analysis.

An interesting aspect of indirect DM detection, when it comes to probe WIMP models, is the fact that if the annihilation cross-section, $\sigma v$, is not velocity dependent, bounds on $\sigma v$ today are directly connected to the DM relic density. In particular, the observation of gamma-rays from dwarf spheroidal galaxies (dSphs) results in stringent limits on the plane of *annihilation cross-section vs WIMP mass*. If for a given channel the annihilation cross-section of $10^{-26}~\mathrm{cm}^3~\mathrm{s}^{-1}$ is excluded for DM masses below 100 GeV, it also means that one cannot reproduce the right relic density for WIMP masses below 100 GeV.^6^ In other words, in this particular case, ID limits will trace the relic density curve. This effect will be clearly visible in many instances.

## Collider searches

LHC proton–proton collisions might result in the production of WIMPs in association with one or more QCD jets, photons as well as other detectable SM debris. Since WIMPs are electrically neutral and cosmologically stable massive particles, they manifest at colliders as missing transverse momentum. For this reason searches for DM are based on the observation of the visible counterpart of the event such as charged leptons, jets or a photon, generally referred to as mono-X searches. By selecting events with large missing transverse momentum/energy one can reduce the SM background and potentially disentangle a DM signal. However, as mentioned above, what colliders identify is missing energy, and therefore they cannot uniquely ascertain the presence of DM in a signal event. They can simply confirm the presence of a neutral and “stable” particle, that might have even decayed outside the detector.

Nevertheless, colliders offer an exciting and complementary search strategy to identify WIMPs. Indeed, assuming that the production of WIMPs at colliders is uniquely connected to the WIMP-nucleon scatterings at underground laboratories, one can use the non-observation signals with large missing transverse momentum to derive limits on the WIMP-nucleon scattering cross-section [123–130].

A large part of this work will be devoted to the study of the cases in which the interactions between DM and SM particles are due to a neutral spin-0 or spin-1 s-channel mediator (see next sections). In this kind of scenario a compelling complementary collider probe is represented by the searches of its “visible” (i.e., into SM final states) decay channels.

We now review in some detail the specifics of the aforementioned search channels for WIMPs at colliders.

### Mono-X searches

Mono-X searches stand for the search of WIMPs produced in association with one or more QCD jets or potentially other SM particles, such as $\gamma$, *h*, *Z* etc. The idea is to search for events with a jet of high transverse momentum $p_T$ within an event with large missing transverse momentum. In particular, the most recent studies performed at the LHC include up to four jets and require the leading jet to have $p_T > 250$ GeV [131, 132], while others do not limit the number of jets while selecting events with at least one jet with $p_T > 100$ GeV [133]. While being more inclusive, these recent searches have become more challenging due to the number of jets analyzed, requiring a substantial improvement on the background coming from $Z+jet$ and $W+jet$ channels.

There are important detector effects, such as fake jets, and QCD backgrounds that weaken the LHC sensitivity to WIMPs, and for these reasons mono-jet searches are subject to large systematics. Nevertheless, fortunately an enormous effort has been put forth in this direction with data driven background and optimized event selections, which combined with the increase in luminosity has led to an overall improvement on the LHC sensitivity to WIMPs.

That said, in the review, we will be using the latest results from CMS and ATLAS collaborations in the search for DM based on mono-X searches [133, 134].

Now we discuss the WIMP production at colliders and also address another collider constraint relevant for the DM purposes which has to do with the invisible decay widths of the SM-Higgs as well as Z bosons.

### Invisible Higgs decays

If WIMPs are lighter than 62.5 GeV, the Higgs boson might invisibly decay into WIMP pairs. In this case, one can use bounds from LHC on the invisible branching ratio (Br) of the Higgs, $\mathrm{Br (h\rightarrow inv)} \le 0.25$ at 95% C.L. [135, 136], to set constraints on WIMP models . Throughout the manuscript whenever applicable we compute the invisible decay rate of the Higgs into WIMPs and impose the aforesaid upper limits to obtain the limits displayed in the figures.

### Invisible Z decays

The decay width of the Z boson has been precisely measured and therefore stringent limits can be derived on any extra possible decay mode of the Z boson. In some of the models as we will discuss further, the DM particle does couple to the Z boson, thus, when mass of the DM is smaller than half of the Z mass stringent limits are applicable. In particular, one can use only direct measurements of the invisible partial width using the single photon channel to obtain an average bound which is derived by computing the difference between the total and the observed partial widths assuming lepton universality. The current limit is $\varGamma \mathrm{(Z\rightarrow inv)} \le 499 \pm 1.5$ MeV [137].

### Searches of visible decays of the mediator

As already mentioned a relevant case of study of this review is represented by the case in which a new neutral field mediates interactions between SM fermion pairs and DM pairs. Provided that its mass is within the reach of a collider, it can be singly produced thanks to its coupling with the SM fermion pairs. The mono-X searches discussed before essentially probe the decay channels of the mediator into DM pairs (the mono-X is radiated by one of the initial state fermions). A potentially even more powerful probe is, however, provided by the visible decay channels of the BSM mediator. Its on-shell production, and subsequent decay into SM states, may lead to spectacular signals represented by dijet and/or dilepton resonances^7^ peaked at the mass of the mediator field. Among the models which will be discussed here these kinds of signal are particularly prominent in the case of spin-1 mediators, since their gauge-like couplings with the SM fermions allow a high production cross-section for these resonances. The considered models with spin-0 mediators feature instead poorer prospects since a Yukawa-type structure will be assumed for their couplings with the SM fermions. We will consequently specialize our discussion to the case of spin-1 resonances.

Among dijet and dilepton resonance searches, the latter have typically the potential to exclude larger portions of the parameters space. This can be understood by noticing the different backgrounds, QCD jets and Drell–Yan production for dijet and dileptons respectively. Dilepton resonances typically represent a much clearer signal, hence provide the strongest constraints unless the couplings of the spin-1 mediator with SM leptons are very suppressed, with respect to the ones with the SM quarks, or even null.

In this work we will mostly consider models with spin-1 mediators with couplings with the SM quarks and leptons of similar size. We have then applied to them the LHC limits from searches of narrow resonances decaying into dileptons, considering an integrated luminosity of $37\,{\text{ fb }}^{-1}$ and 13 TeV of center-of-mass energy [138]. Under the assumption, considered in [138], that the new spin-1 neutral resonance is coupled only with the SM states, the present absence of signals is translated, according the assignations of the couplings of the spin-1 mediator, into very stringent lower bound on its mass. Masses below 4 TeV are, for example, excluded in the case when the couplings of the mediator with quarks and leptons are of the same size of the ones of the *Z*-boson. This picture might be potentially altered by the presence of the DM. In case the decay of the spin one mediator into a DM pair is kinematically allowed, the actual limit follows the simple rescaling relation given by [139]:

20 $$\begin{aligned} \sigma _\mathrm{ll}=\left( 1-\mathrm{Br}_\mathrm{DM}\right) \sigma _\mathrm{ll}^{exp}, \end{aligned}$$

where $\mathrm{Br}_\mathrm{DM}$ is the decay branching fraction of the neutral spin-1 resonance into DM pairs and $\sigma _\mathrm{ll}^{exp}$ is the limit obtained from experimental searches. It is then evident that the limit on the mass of the spin-1 mediator from the searches of its visible decay channels is actually also dependent on the DM mass and couplings.

## Model setup: dark portals

In order to maximally profit of the information from the different kind of experimental searches we need an efficient interface between the experimental outcome and theoretical models. The processes responsible for the DM relic density and its eventual detection can be described by simple extensions of the SM in which a DM candidate interacts with the SM states (typically the interactions are limited to the SM fermions) through a mediator state (dubbed portal). This idea is at the base of the so-called “Simplified Models” [140–154] which are customarily adopted especially in the context of collider studies, see e.g., Refs. [130, 155–166].

Keeping a similar spirit we will discuss a selection of DM setups, with increasing degree of refinement, which we will globally refer to as “Dark Portals”. These are characterized by spin-0, spin-1/2 and spin-1 DM candidates (we will distinguish whenever relevant the cases of self- and not self-conjugated DM) interacting with the SM fields, typically the electrically neutral bosons, and/or possibly a spin-0 or spin-1 mediator field.^8^

A schematic representation of the Dark Portal models considered in this work is provided by the following Lagrangians (for simplicity we will report only the interaction terms):

21 $$\begin{aligned} {\mathscr {L}}= & {} \xi \mu _\chi ^S \lambda _\chi ^S \chi \chi S +\xi (\lambda _\chi ^S)^2 \chi ^2 S^2+\frac{c_S}{\sqrt{2}}\frac{m_f}{v_h}\overline{f} f S,\nonumber \\ {\mathscr {L}}= & {} \xi g_\psi \overline{\psi }\psi S+\frac{c_S}{\sqrt{2}}\frac{m_f}{v_h}\overline{f} f S,\nonumber \\ {\mathscr {L}}= & {} \mu _V^S \eta _V^S V^\mu V_\mu S+\frac{1}{2}(\eta _V^S)^2 V^\mu V_\mu SS\nonumber \\&+\frac{c_S}{\sqrt{2}}\frac{m_f}{v_h}\overline{f} f S, \end{aligned}$$

22 $$\begin{aligned} {\mathscr {L}}= & {} i g \lambda _\chi ^{Z'} \left( \chi ^{*} \partial _\mu \chi -\chi \partial _\mu \chi ^{*}\right) Z^{'\,\mu } \nonumber \\&+\,g^2 (\lambda _\chi ^{Z'})^2 |\chi |^2 Z^{'\mu }Z^{'}_\mu +g \overline{f} \gamma ^\mu (V_f^{Z^{'}}-A _f^{Z^{'}} \gamma _5) Z_\mu ^{'} f, \nonumber \\ {\mathscr {L}}= & {} g \xi \overline{\psi }\gamma ^\mu (V_\psi ^{Z^{'}}-A _\psi ^{Z^{'}} \gamma _5)\psi Z_\mu ^{'}\nonumber \\&+\,g \overline{f} \gamma ^\mu (V_f^{Z^{'}}-A _f^{Z^{'}} \gamma _5)f Z_\mu ^{'}, \nonumber \\ {\mathscr {L}}= & {} g \eta _V^{Z'} [[VVZ^{'}]]+g \overline{f} \gamma ^\mu (V_f^{Z^{'}}-A _f^{Z^{'}} \gamma _5)f Z_\mu ^{'}, \nonumber \\ {\mathscr {L}}= & {} g \eta _V^{Z'}\epsilon ^{\mu \nu \rho \sigma }V_\mu Z_\nu V_{\rho \sigma } +g\overline{f} \gamma ^\mu (V_f^{Z^{'}}-A _f^{Z^{'}} \gamma _5)f Z_\mu ^{'}.\nonumber \\ \end{aligned}$$

We will describe in detail the different Lagrangians in dedicated subsections. A brief overview of some relevant details will nevertheless be provided at the end of this section.

We have generically labeled a (real) scalar^9^ and vector mediators as *S* and $Z^{'}$, respectively. We have indicated a scalar, fermionic and vectorial DM as $\chi$, $\psi$ and *V*, respectively while *f* generically refers to a SM fermion. Throughout this work we refer to models described by Lagrangians of the type as schematically shown in Eqs. (21) and (22) as “s-channel portals” since the DM annihilation processes into the SM states are described by diagrams in which the mediator is exchanged in the s-channel. We will also assume that the s-channel mediators interact with all the SM fermions (sums over all the SM fermion species are, hence, implicitly intended in Eqs. (21) and (22)). In addition to these models we will consider “t-channel portals”, schematically described by:

23 $$\begin{aligned} {\mathscr {L}}&=\lambda _{\varPsi f} \overline{\varPsi }_f \chi f+\text{ h.c. }, \nonumber \\ {\mathscr {L}}&=\lambda _{\Sigma _f} \overline{\psi }\Sigma _f f +\text{ h.c., } \end{aligned}$$

with $\Sigma _f$ and $\varPsi _f$ being, respectively, a scalar and a fermionic field with the same quantum numbers as of the SM fermion *f*. Here annihilations into the SM states are associated with the exchange of mediator field in the t/u-channel. Contrary to the case of s-channel mediators, which can be coupled with all the SM fermions, t-channel mediators, according their quantum number, are coupled only to one type of the SM fermions, i.e., left/right-handed leptons, left/right-handed up-type quarks or left/right-handed down-type quarks.

Some further comments about Lagrangians of Eqs. (21) and (22) are discussed subsequently. The trilinear coupling between a spin-0 mediator and spin-0 (spin-1) DM is dimension-full. This has been re-expressed as the product of a dimensionless quantity $\lambda _\chi ^S$ ($\eta _V^S$) and a mass scale $\mu _\chi ^S$ ($\mu _V^S$). We will assign specific value, according to the considered theoretical framework, for the latter while regards $\lambda _\chi ^S$ ($\eta _V^S$) as free parameter. The coupling of the scalar mediator with the SM fermions has been written as the SM Yukawa coupling, i.e., $m_f/{\sqrt{2}v_h}$, times a free scaling factor $c_S$. The first two Lagrangians in Eq. (21) and the second Lagrangian of Eq. (22) are substantially equivalent for self-conjugate (i.e., real scalar or Majorana fermion) and non self-conjugate (i.e., complex scalar and/or Dirac fermion) DM with $\xi =1/2$ and 1, respectively. For this reason we are explicitly reporting Lagrangians only for the case of a real scalar and a Dirac fermionic DM. Note, however, that $V_\psi ^{Z'}$ is identically zero for a Majorana fermionic DM. The first Lagrangian of Eq. (22) can only be written for a complex scalar DM. In the case of a spin-1 DM with spin-1 mediator, two sensitively different Lorentz structures are allowed for non-self conjugate (third Lagrangian of Eq. (22)) and self-conjugate DM (fourth Lagrangian of Eq. (22)) which we have reported individually. In the case of a non self-conjugate vectorial DM the interactions are substantially analogous to the one of two SM *W* bosons with the *Z* boson. Thus,

24 $$\begin{aligned}{}[[VVZ^{'}]]\equiv & {} i \left[ V_{\mu \nu } V^{\dagger \, \mu }Z^{'\nu }-V^\dagger _{\mu \nu } V^{\mu }Z^{'\nu }\right. \nonumber \\&\left. +\,\frac{1}{2}Z_{\mu \nu } \left( V^\mu V^{\dagger \,\nu }-V^\nu V^{\dagger \,\mu }\right) \right] , \end{aligned}$$

and for this reason we will often refer to this scenario as non-Abelian DM. We, however, will use Abelian DM for the case of a self-conjugate vectorial DM coupled through the Levi-Civita operator to the spin-1 mediator. We finally remark that in all the cases when the DM is interacting with a spin-1 mediator, we have normalized the couplings with the SM gauge coupling *g*. The Lagrangians of Eqs. (21)–(23) describe interactions of the DM candidates which are singlet under the SM gauge group. In the last part of this work we will, instead, relax this assumption and consider a DM (at least partially) charged under EW interactions. These kind of models have a richer phenomenology and are better motivated from the theoretical point of view though they still feature a limited number of free parameters. In addition they are still somehow correlated to the Dark Portal models and Eq. (21), Eq. (22) represent a possible UV completion for some of them.

In summary we will discuss a collection of models mostly built according to a bottom-up approach and maintains a substantial degree of simplicity. Nevertheless, contrary to the aforementioned “simplified models”, they also aim at a more faithful description of theoretically motivated scenarios. For example, in most cases we will adopt specific choices of the parameters inspired by possible theoretical completions of the models under consideration. In the same fashion we will account, in our analysis, besides experimental constraints, the theoretical limitations of these frameworks (see e.g., Refs. [151, 152, 161, 162, 168] for more extensive discussions.)

Besides these considerations the choice of the models under study is dictated by the main purpose, already stated in the introduction, of assessing the capability of present and, more importantly, near future DD facilities of probing the WIMP paradigm. For each of the models under scrutiny we will determine the portion of the parameter space excluded by current DD limits, as set by the XENON1T experiment, and the parameter space which would be ruled out in case of absence of signals, after 2 years of exposure time at the XENON1T experiment itself, and at the multi-TON detector LZ. Most of the considered models will then be characterized by efficient interactions of the DM with nucleons. At the same time we will discuss possible scenarios capable of surviving even eventual limits from high sensitivity future experiments like LZ. Limits from other kind of DM searches, i.e., ID and LHC, will not be neglected when competitive or complementary with the ones from DD.

To make simpler the orientation among the many different models which will be individually discusses in the following sections we have adopted the following classification:

1. SM portals: These represent a special case of s-channel portals, i.e., Eqs. (21) and (22), in which $S=h$ and $Z^{'}=Z$ with *h* and *Z* being the SM Higgs and *Z* bosons, respectively.^10^ These are the most predictive models since the couplings of the mediators with the SM states are fixed. Moreover, $\mu _\chi ^h=\mu _V^h=v_h$. These leave us with just two free-parameters, the DM mass and an adimensional coupling. On the other hand, these models are also the most strongly constrained ones so that they are already strongly disfavored by the present limits;
2. BSM s-channel portals: These are the models fully described by Eqs. (21) and (22). Here the DM is coupled with the SM fermions either by a new spin-0 (real) or by a spin-1 electrically neutral state. These scenarios, besides the SM portals, are the most sensitive ones to DD of the DM;
3. Portals evading DD: Here we will instead consider the case of a pseudoscalar s-channel mediator, in which constraints from DD are particularly weak so that the complementarity with other search strategies becomes crucial. We will also considered a more theoretically refined scenario in which the pseudoscalar mediator is part of a complex scalar field. The presence of an additional scalar component will reintroduce DD bounds. A broad region of the parameter space for thermal DM is nevertheless re-opened by considering a very light pseudoscalar, which can be interpreted as a pseudo-Goldostone boson of a global symmetry carried by the original complex scalar field;
4. t-channel portals: In this alternative version of the Dark Portals the mediator field has non-trivial quantum numbers with respect to the SM gauge group. In these kind of setups DM annihilation arise from t-channel interactions while s-channel interactions are responsible for DD;
5. Portals to secluded sectors: We assume here that the mediator field cannot be directly coupled with the SM fermions. Even in these kind of constructions a portal can be originated by mass mixing with the SM Higgs, in the case of spin-0 mediators, and by kinetic mixing with the Z-boson in the case of a spin-1 field, so that the DM actually interacts through a double s-channel mediator, represented by a SM and a BSM state;
6. Portals with DM (partially) charged under $SU(2) \times U(1)$: In these models the DM is charged under the EW symmetry group, being either the lightest electrically neutral component of a (or more) *SU*(2) multiplet (we will discuss in detail only the case of *SU*(2) doublets) or a mixed state between the latter and a SM singlet. Note that this category of models also contain renormalizable completions of the Higgs and Z-portal models although the actual phenomenology features sensitive differences.

We also remark that we have assumed, throughout all our study, real couplings and hence, no CP-violation.

The SM portals are the simplest models since they are characterized by only two free parameters, the DM mass and couplings. As a consequence the comparison between DM relic density and DD limits/prospects (and eventual additional constraints) can be performed in full generality in a bi-dimensional plane of the two quantities. s- and t-channel portals, as appear for the aforementioned model categories 2, 3 and 4, feature two mass scales, the DM mass and the mediator, and one or at most two relevant couplings (in some cases this actually implies additional assumptions, clarifications will be provided in the dedicated sections). For these scenarios we have adopted the two masses as freely varying parameters and assigned $\mathscr {O}(1)$ values to the couplings unless this option is precluded by theoretical considerations.

There is very modest loss of generality in this choice. The DM annihilation and scattering cross-section feature a very similar dependence on the free couplings, lower values of the couplings correspond to weaker limit/prospect from DD but, at the same time, progressively disfavor the achievement of the correct relic density so that the overall picture is only marginally affected. For the last category of models the interplay between DD and relic density is less trivial. In this case we have considered, besides representations in bi-dimensional planes of two relevant parameters, also scans in which all the free parameters of the models are varied.

Concerning, in particular, the DM mass, we have typically assumed a range of variation from 10 GeV to a few TeV. The choice of the lower bound is motivated by our focus on DD as DM search strategy. For masses below 10 GeV, experimental sensitivity is strongly reduced because of the approaching of the energy threshold of the experiments under consideration. We also remark that achieving the correct relic density becomes increasingly more difficult as the DM mass decreases, because of the reduced number of accessible final states (for this reason in the case of DM interactions mediated by BSM fields the lowest value of the DM mass shown in the figures is 100 GeV). Finally for several realizations, namely the cases in which the annihilation cross-section is velocity independent (s-wave), thermal DM below 10 GeV is already ruled out by ID as well as CMB measurements [1].

Concerning the maximal value of the DM mass, i.e., a few TeV, this should be regarded mostly as an assumption. We remark, nevertheless, that in bottom-up setups it might result difficult to accommodate viable thermal DM without encountering issues of theoretical consistency. In other cases, like the ones discussed in Sect. 12, for a high value of the DM mass the correct relic density cannot be achieved because of a systematic suppression of the annihilation cross-section. Relevant viable WIMP scenarios, featuring DM heavier than a few TeV, like the so-called “minimal DM”, have been proposed in Refs.  [169–171]. These kind of models will not be explicitly revised here; we will nevertheless discussed them in slightly more detail in Sect. 12.

## SM portals

The first class of models which will be the object of study are the SM Dark Portals,^11^ i.e., models in which the DM interacts with the SM state through the Higgs or the Z-boson. In the case when the DM is a pure SM singlet, gauge invariant renormalizable operator connecting the DM with the Z or the SM-Higgs boson can be build only in the latter case and only for scalar and vectorial DM. In the other cases one should rely either on higher dimensional operators, or on the case that the coupling with the Higgs and/or the Z is originated by their mixing with new neutral mediators. The latter case can imply the presence of additional states relevant for the DM phenomenology and will be then discussed later on in the text. We will instead quote below some example of higher dimensional operator but we will not refer to any specific construction for our analysis. Alternatively one could assume that the DM has some small charge under $SU(2)_L$ or $U(1)_Y$, see e.g., Refs. [173–179]. We will not review these scenarios here.

### Higgs portal

The most economical way to connect a SM singlet DM candidate with the SM Higgs doublet *H* is through four field operators built to connect the Higgs bilinear $H^\dagger H$, which is a Lorentz and gauge invariant quantity, with a DM bilinear. Assuming CP conservation, the possible^12^ operators connecting the SM-Higgs doublet with scalar, fermion and vector DM are given by [181–191]:

25 $$\begin{aligned} \xi \lambda ^H_{\chi } \chi ^{*} \chi H^\dagger H, \,\,\, \xi \frac{\lambda ^H_{\psi }}{\varLambda } \overline{\psi }\psi H^{\dagger } H \quad \mathrm{and}\quad \xi \lambda ^H_V V^\mu V_\mu H^\dagger H, \end{aligned}$$

where, in the unitary gauge, $H ={\left( 0\,\,\,\frac{v_h+h}{\sqrt{2}}\right) }^T$ with $h,\,v_h$ denoting the physical SM Higgs boson, Vacuum Expectation Value (VEV) and $\xi =1/2 (1)$ in case the DM is (not) its own antiparticle. From Eq. (25) note that stability of the DM is protected either by a discrete ${\mathbb {Z}}_2~(\mathrm{for}~ \psi ,\,V_\mu$ and when $\chi =\chi ^*$) or by a $U(1)~(\mathrm{for}~\chi \ne \chi ^*)$ symmetry.

As already pointed out, in the case of a scalar and vectorial DM it is possible to rely on a dimension-4 renormalizable operator; on the contrary a fermionic DM requires at least a dimension-5 operator which depends on an unknown Ultra-Violet (UV) scale $\varLambda$.

After EW symmetry breaking (EWSB), trilinear couplings between the Higgs field *h* and DM pairs are induced. In the case of fermionic DM it is possible to absorb the explicit $\varLambda$ dependence by a redefinition of the associated coupling, i.e., $\lambda _\psi ^H \frac{v_h}{\varLambda }$ as $\lambda _\psi ^H$, so that it does not appear explicitly in computations.

The models defined by Lagrangians of Eq. (25) have only two free parameters, the DM masses $m_{\chi ,\psi ,V}$ and couplings $\lambda _{\chi ,\psi ,V}^H$ with the SM-Higgs. The constraints on these models can be then easily summarized in bi-dimensional (i.e., DM mass vs its coupling with the SH-Higgs) planes.

**Fig. 5:**
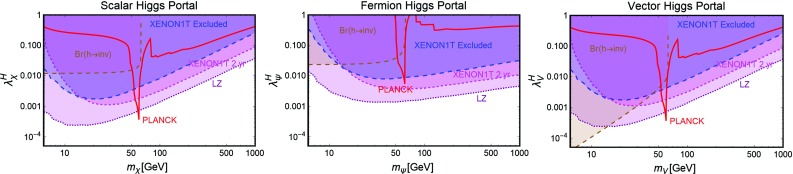
Illustration of the SM-Higgs portal in the relevant bi-dimensional planes for a scalar (left panel), fermionic (middle panel) and vectorial (right panel) DM. In each plot, the red line represents the model points featuring the correct DM relic density. The blue region is excluded by the current SI DD limits. The magenta region would be excluded in case of absence of signals in XENON1T after 2 years of exposure time while the purple region is within the reach of future LZ limits. Finally, the brown region is excluded because of a experimentally disfavored invisible decay branching fraction of the SM-Higgs boson

In Fig. 5 we summarize our results for scalar, fermionic and vectorial DM, respectively. All the plots report basically three set of constraints.^13^ The first one (red contours) is represented by the achievement of the correct DM relic density.^14^ The DM annihilates into SM fermions and gauge bosons, through s-channel exchange of the SM-Higgs boson, and, for higher masses, also into Higgs pairs through both s- and t-channel diagrams (in this last case a DM particle is exchanged). Since the coupling of the SM-Higgs with SM fermions and gauge bosons depends on the masses of the particles themselves, the DM annihilation cross-section is suppressed, at the exception of the pole region $m_\chi \sim m_h /2$, until the $W^+W^-$, *ZZ* and $\overline{t} t$ final states are kinematically accessible. Even in this last case, the cosmologically allowed values for the couplings are in strong tension with the constraints from DD of the DM, which for all the considered spin assignation of the DM, arise from SI interactions of the DM with the SM quarks originated by t-channel exchange of the SM-Higgs boson. As can be easily seen that the entire parameter space corresponding to thermal DM is already ruled out, at the exception, possibly, of the pole region, for DM masses at least below 1 TeV. Eventual surviving resonance regions will be ruled-out in case of absence of signals at the forthcoming XENON1T, assuming a 2 years of exposure time. As expected, the most constrained scenario is the fermionic DM one because of the further suppression of the p-wave suppression of its annihilation cross-section.

Notice that, scalar and vectorial DM, due to the s-wave annihilation cross-section, might also be probed through ID. The corresponding limits are nevertheless largely overpower by the ones from DD and hence, have been then omitted for simplicity. The limits from DD experiments are complemented at low DM masses, i.e., $m_{\chi ,\psi ,V} < m_h/2$, by the one from invisible decay width of the Higgs. Indeed this constraint would exclude DM masses below the energy threshold of DD experiments. Our findings are in agreement with the other recent studies in the topic [143, 145, 192–205].

### Z-portal

An interaction between the *Z*-boson and a SM singlet DM candidate is not gauge invariant for any dimension-4 operators. In the case of scalar and fermionic DM models, the simplest option is to consider a dimension 6 operator^15^ [178, 206–208]. In the case of scalar DM it is of the form:

26 $$\begin{aligned} {\mathscr {L}}= \lambda _\chi \frac{H^\dagger \overleftrightarrow {D^{\mu }} H}{\varLambda ^2} \chi ^* \overleftrightarrow {\partial _{\mu }} \chi , \end{aligned}$$

which give rise to a trilinear interaction between the *Z*-boson and a DM pair once the SM-Higgs field in the Lagrangian is replaced by its VEV, so that $H \overleftrightarrow {D^\mu } H \rightarrow \frac{g v_h^2}{4 \cos \theta _W}Z^\mu$. $\varLambda$ is again the relevant cutoff scale of the effective theory. Similar to the case of a fermionic DM in Higgs portal we can absorb it in the definition of a dimensionless coupling as $\lambda ^Z_\chi \equiv \lambda _\chi v^2_h/\varLambda ^2$.

In addition, after EWSB, an effective dimension-4 interaction like $(g^2/16\cos ^2\theta _W)\lambda ^{ZZ}_{\chi \chi }{|\chi |}^2 Z^\mu Z_\mu$ can emerge from the dimension-6 SM gauge invariant operator $\lambda _{\chi \chi }$ $(D^\mu H)^\dagger$ $D_\mu H {|\chi |}^2/\varLambda ^2$ such that $\lambda ^{ZZ}_{\chi \chi }=\lambda _{\chi \chi }v^2_h/\varLambda ^2$. For simplicity we maintain a rescaling with powers of the $SU(2)_L$ gauge coupling *g*.

The interaction Lagrangian for the DM, along with the relevant SM parts, can thus be written as:

27 $$\begin{aligned} {\mathscr {L}}= & {} i \frac{g}{4 c_W} \lambda _\chi ^Z \chi ^* \overleftrightarrow {\partial _{\mu }} \chi Z^\mu +\frac{g}{4 c_W} \sum _f \overline{f} \gamma ^\mu \left( V_f^Z-A_f^Z \gamma _5\right) f Z_\mu \nonumber \\&+\,\frac{g^2}{16 c^2_W} \lambda ^{ZZ}_{\chi \chi } {|\chi |}^2 Z^\mu Z_\mu , \end{aligned}$$

where $c_W=\cos \theta _W$ and $\theta _W$ is Weinberg angle [137, 209]. Note that we have used a normalization of $g/4\cos \theta _W$ throughout in analogy to the SM $\overline{f} f Z$ couplings.

The interaction Lagrangian for fermion DM is built in a similar fashion as the scalar case. In the case of Dirac DM the starting operator is:

28 $$\begin{aligned} {\mathscr {L}}= \frac{H^\dagger \overleftrightarrow {D_\mu } H}{\varLambda ^2}\left( \overline{\psi }\gamma ^\mu \left( v_\psi ^Z-a^Z_\psi \gamma ^5\right) \psi \right) , \end{aligned}$$

which, after the EWSB, together with the apposite SM part leads to:

29 $$\begin{aligned} {\mathscr {L}}= & {} \frac{g}{4 \cos \theta _W} \overline{\psi }\gamma ^\mu \left( V_\psi ^Z-A^Z_\psi \gamma ^5\right) \psi Z_\mu \nonumber \\&+\, \frac{g}{4 \cos \theta _W} \sum _f \overline{f}\gamma ^\mu \left( V^Z_f-A^Z_f \gamma ^5\right) f Z_\mu , \end{aligned}$$

with ${V}_\psi ^Z={v}_\psi ^Z \frac{v_h^2}{\varLambda ^2}$ and ${A}_\psi ^Z={a}_\psi ^Z \frac{v_h^2}{\varLambda ^2}$. In the case of Majorana DM $V_\psi ^Z=0$ and we rescale the remaining DM coupling by a factor of 1 / 2.

In the case of spin-1 DM we will consider two possible kind of interactions, namely, self- (Abelian) and not self-conjugated (non-Abelian) DM, respectively. For the latter, along with the necessary SM parts, we can write the following Lorentz invariant interaction:

30 $$\begin{aligned}&{\mathscr {L}}=\frac{g}{4 \cos \theta _W} \eta ^Z_V [[VVZ]]\nonumber \\&\qquad +\, \frac{g}{4 \cos \theta _W} \sum _f \overline{f}\gamma ^\mu \left( V^Z_f-A^Z_f \gamma ^5\right) f Z_\mu ,\nonumber \\&\mathrm{with~~}[[VVZ]] \equiv i \left[ \frac{1}{2}V_{\mu \nu } V^{\dagger \, \mu }Z^\nu -V^\dagger _{\mu \nu } V^{\mu }Z^\nu \right. \nonumber \\&\quad \left. +\,\frac{1}{2}Z_{\mu \nu } \left( V^\mu V^{\dagger \,\nu }-V^\nu V^{\dagger \,\mu }\right) \right] , \end{aligned}$$

where $V_{\mu \nu },V^\dagger _{\mu \nu },Z_{\mu \nu }$ represent the respective field strengths. In Eq. (30) the [[*VVZ*]] coupling is normalized as $g/4\cos \theta _W$ while the model specific information are parametrized as $\eta ^Z_V$.

In the case of self-conjugate spin-1 DM, an interaction with the gauge boson can be built through the Levi-Civita symbol as Ref. [210]:

31 $$\begin{aligned} {\mathscr {L}}= & {} \frac{g}{4 \cos \theta _W} \eta ^{Z}_V \epsilon ^{\mu \nu \rho \sigma } V_{\mu } Z_{\nu } {V}_{\rho \sigma } \nonumber \\&+\,\frac{g}{4 \cos \theta _W}\sum _f\overline{f}\gamma ^\mu \left( V^{Z}_f-A^{Z}_f \gamma ^5\right) f Z_\mu . \end{aligned}$$

Similar to the previous cases the coupling $\eta _V^Z$ in Eq. (31) encodes a cut-off scale (see e.g., Refs. [211–214] for the construction of effective theories). The theoretical derivation of Eq. (30) is however, more contrived.

**Fig. 6:**
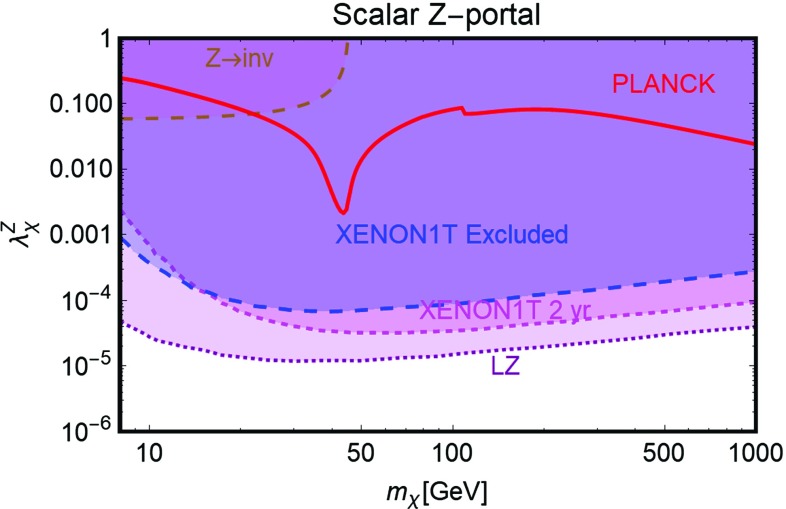
Combined constraints for Z-portal with scalar DM in the $m_\chi$-$\lambda ^Z_\chi$ bi-dimensional plane. Color specifications are the same as Fig. 5, except the fact that now the brown region represents experimentally excluded invisible decay width of *Z*-boson

**Fig. 7:**
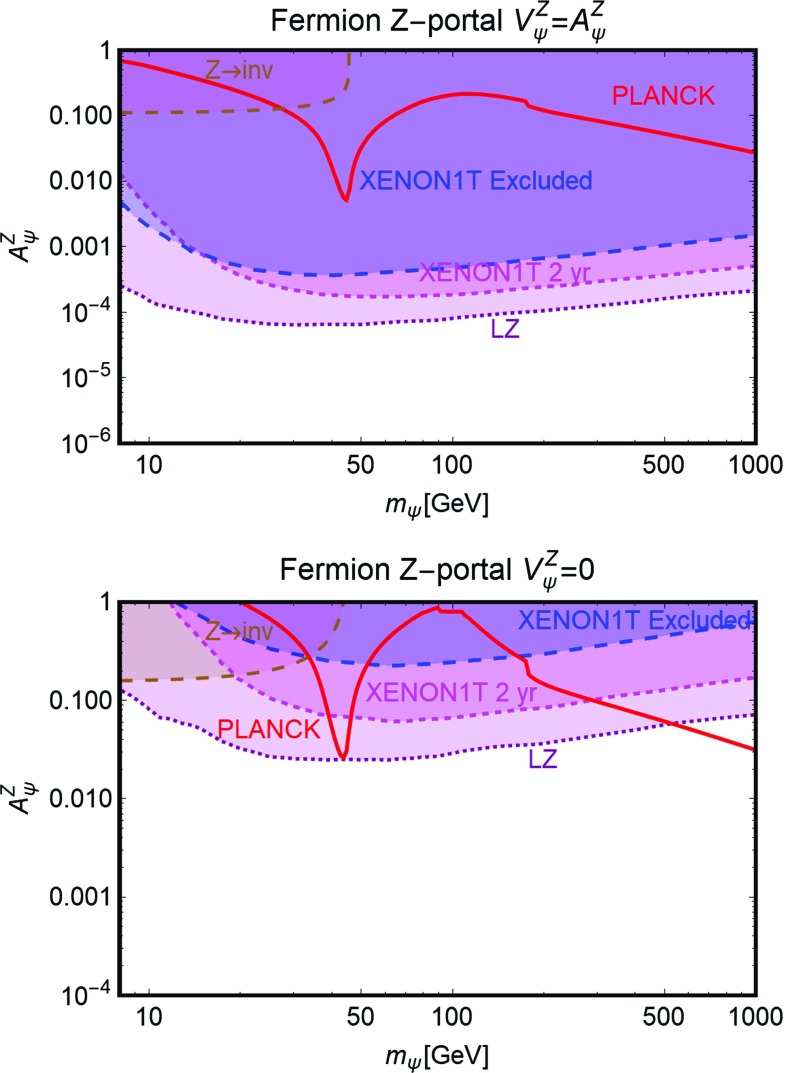
The same as Fig. 6 but for Dirac fermion DM in the relevant bi-dimensional plane $m_\psi$-$A^Z_\psi$ with both vectorial and axial couplings (top panel), set to the same value, and only axial couplings (bottom panel) with the *Z*-boson

**Fig. 8:**
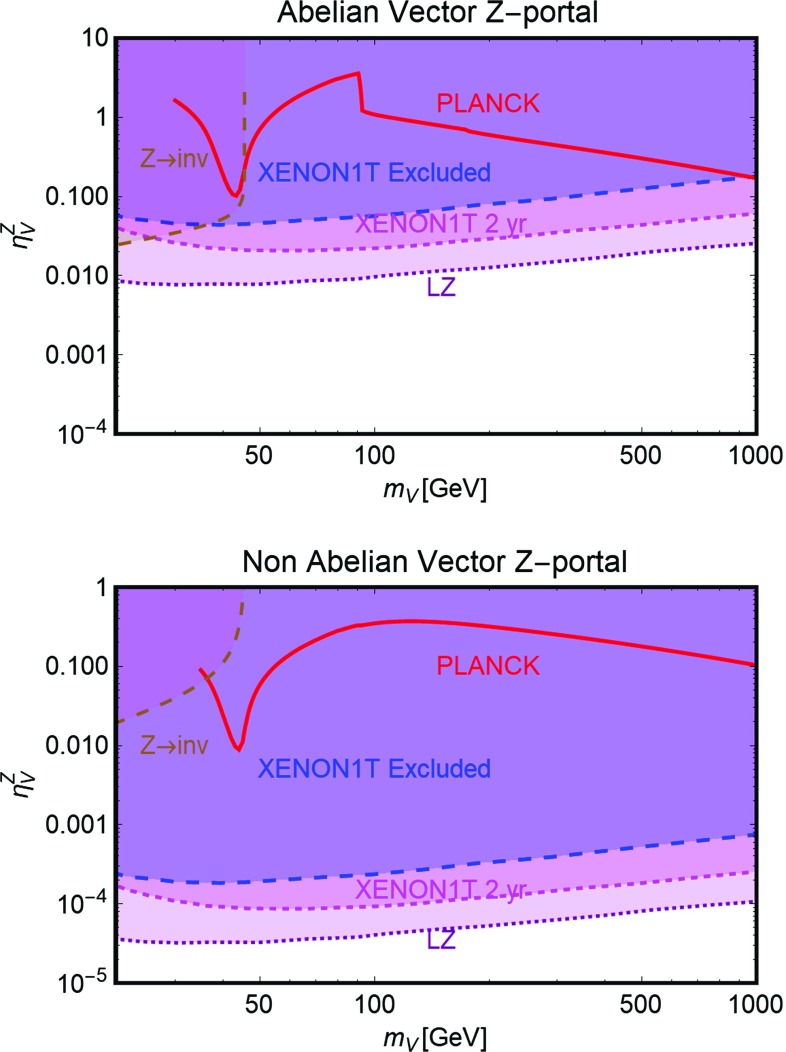
The same as Fig. 6 but for vectorial DM with (i) Abelian case (top panel) and (ii) non-Abelian case (bottom panel) in the pertinent bi-dimensional plane $m_V$-$\eta ^Z_V$

Further, similar to the Higgs portal, the Z-portal models are fully defined by two parameters so that one can repeat the same kind of analysis performed in the previous subsection. The results are summarized in Figs. 6, 7 and 8.^16^

As evident, in all but the Majorana *Z*-portal case, thermal DM is already excluded, even for masses above the TeV scale, by current constraints from XENON1T. These constraints are even stronger with respect to the case of the SM-Higgs portal. This is because, apart from the lighter mediator, the scattering cross-section on Xenon nuclei is enhanced by the isospin violating interactions of the *Z* with light quarks. Low DM masses, possibly out of the reach of DD experiments, are instead excluded by the limit on the invisible decay width of the *Z*-boson. As already pointed out, the only exception to this picture is represented by the case of Majorana DM where the SI component of the DM scattering cross-section is largely suppressed due to the absence of a vectorial coupling of the DM with the *Z*. This scenario is nevertheless already (partially) within the reach of current searches for a SD component of the scattering cross-section. The increased sensitivity of XENON1T will allow to exclude DM masses below 300 GeV, except the “pole” region. The latter, however, will meet the same fate from a projected future LZ sensitivity.

## BSM s-channel portals

The results presented in the previous cases for the SM-Higgs and *Z*-boson portals will be generalized and discussed in more details in the case of generic, BSM spin-0 and spin-1 mediators interacting with a pair of scalar, fermion or vector DM fields. Contrary to the case of SM portals, interactions of the mediators with the gauge bosons are not mandatory. We will thus stick, in this section to the case, analogous to the so-called simplified models, in which the DM is coupled only to the SM fermions. The case of interactions with gauge bosons will be discussed separately later in the text, where unlike the aforementioned models, we will also assume interactions with both quarks and leptons.

### Spin-0 portals

#### Scalar dark matter

We will consider the following Lagrangian:

32 $$\begin{aligned} {\mathscr {L}}=-\xi \mu _\chi ^S {|\chi |}^2 S- \xi \lambda _\chi ^{S} {|\chi |}^2 S^2 - \frac{c_S}{\sqrt{2}} \frac{m_f}{v_h}\overline{f} f S, \end{aligned}$$

where *S* is a real scalar field and $\xi$ denotes the normalization factor, accounting, similar to the previous section, for the case when the DM coincides (or not) with its own antiparticle. In the case of SM fermions we have assumed a Yukawa-like structure of the couplings with the mediator while for the scalar DM $(\chi )$ we have parametrized all the information, including possible normalization factors (e.g., factor of 1 / 2 in the second term of Eq. (32)), in the respective couplings. Note that $\mu _\chi ^S$ parameter has the dimension of mass. Unless differently stated we will assume $\mu _\chi ^S=\lambda _\chi ^S m_S$ with $\lambda _\chi ^S$ being a dimensionless coupling and $m_S$ as the mass of *S*. We will also add self interaction term for the scalar field given by:

33 $$\begin{aligned} {\mathscr {L}}_S=-\frac{1}{3!}m_S \lambda _S S^3. \end{aligned}$$

The assignation for the dimensional couplings, as well as the introduction of the Lagrangian term in Eq. (33), are inspired to scenarios in which the scalar field *S* acquires a VEV. In this setup the Lagrangian of Eq. (33) originates from the quartic term in the scalar potential whose presence cannot be forbidden by any symmetry argument. In the same fashion a quartic interaction term $S^2 H^\dagger H$ with the SM-Higgs doublet, responsible for a mixing of the *S* and *h* states, should also be included. For simplicity we will assume here that the coupling of this last operator is negligible and postpone the discussion of the most general case to a dedicated section.

**Fig. 9:**
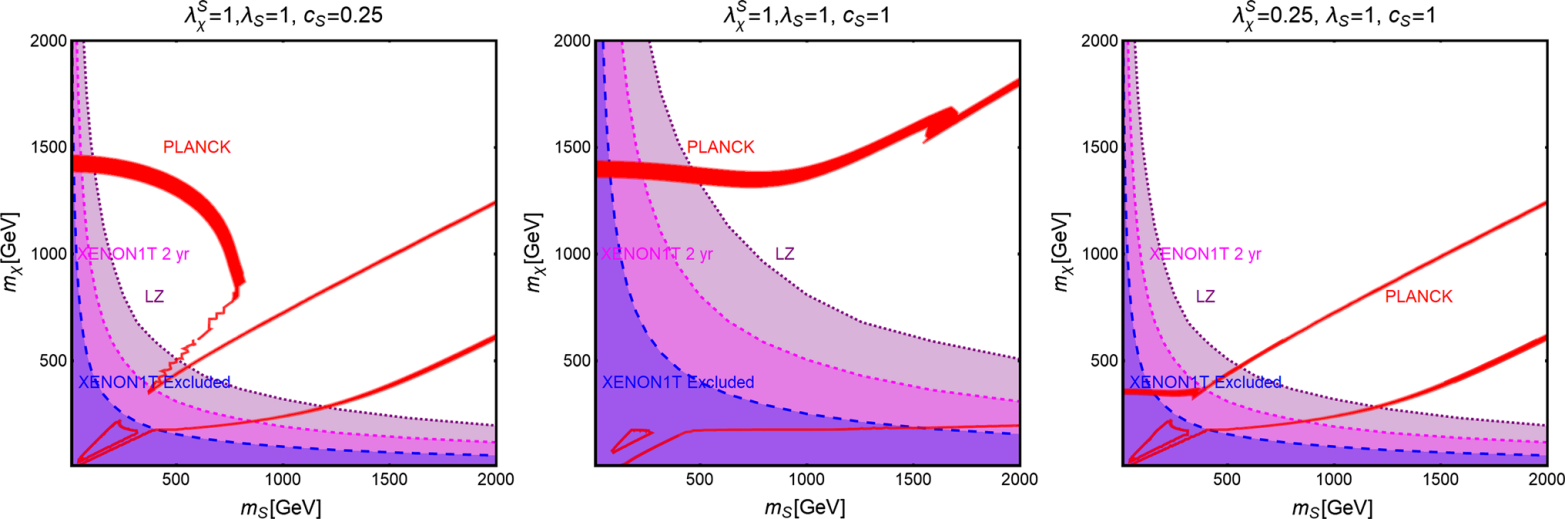
Combined constrains for a scalar DM with scalar mediator scenario in the bi-dimensional plane $(m_S,m_\chi )$ for three assignations of the relevant couplings, i.e., $(\lambda _\chi ^S,\,\lambda _S,\,c_S) =(1,\,1,\,0.25)$ (left), $(1,\,1,\,1)$ (middle) and $(0.25,\,1,\,1)$ (right). Here the iso-contours of the correct DM relic density are represented by red bands. The blue, magenta and purple regions represent the current exclusion and the projected sensitivity of XENON1T (assuming 2 years of exposure time in the latter case) and LZ, respectively

Contrary to the case of the SM portals, which have only the DM mass and its coupling as free parameters, we have expressed, as reported on Fig. 9, our main results in the bi-dimensional plane $(m_\chi ,m_S)$ for the three free coupling assignations $(\lambda _\chi ^S,\lambda _S,c_S)=(1,1,0.25)$, (1, 1, 1), (0.25, 1, 1). Figure 9 hence shows the comparison between current DD limits, as well as the projected sensitivities from XENON1T and LZ, and the requirement of the correct DM relic density.

The results reported in Fig. 9 can be explained as follows. A t-channel exchange of the scalar mediator induces SI interactions of the DM, which are written, in the case of the proton as:

34 $$\begin{aligned} \sigma _{\chi p}^\mathrm{SI}~= & {} \frac{\mu _{\chi p}^2}{4 \pi }\frac{{(\lambda _\chi ^S)}^2 c_S^2}{m_\chi ^2 m_S^2} \frac{m_{p}^2}{v_h^2}{\left[ f_p \frac{Z}{A}+f_n \left( 1-\frac{Z}{A}\right) \right] }^2,\nonumber \\\approx & {} 1.8 \times 10^{-45}{\text{ cm }^2}\nonumber \\&\times \, {(\lambda _\chi ^S)}^2 c_S^2 {\left( \frac{400~\text{ GeV }}{m_S}\right) }^2 {\left( \frac{400~\text{ GeV }}{m_\chi }\right) }^2. \end{aligned}$$

Here *A*, *Z* represent, respectively, the atomic and proton number of the material constituting the detector, $\mu _{\chi p}=m_\chi m_p/(m_\chi +m_p)$ denotes reduced mass of the WIMP-proton system with $m_p$ representing the mass of the latter while $f_p$ and $f_n$ represent the effective couplings of the DM with protons and neutrons. In the case of a scalar mediator we have:

35 $$\begin{aligned} f_N= & {} \sum _{q=u,d,s} f_q^N+\frac{6}{27}f^N_\mathrm{TG},\nonumber \\ f^N_\mathrm{TG}= & {} 1-\sum _{q=u,d,s} f_q^N,\quad N=p,n, \end{aligned}$$

with $f_N\,(f_q^N)$ being the form factor whose physical meaning is associated to the contribution from all the six quark flavours (up, down and strange quark flavours) to the mass of the proton and the neutron. The contribution of the heavy quarks in $f_N$ is described by a unique form factor $f_{c}^N=f_{b}^N=f^N_{t}=\frac{2}{27}f_{TG}^N$ (in Eq. (35) we have implicitly summed over the three heavy quark flavours). For their numerical values we have adopted the default assignations by micrOMEGAs. Notice that the factor ${\left[ f_p \frac{Z}{A} +f_n \left( 1-\frac{Z}{A}\right) \right] }$ is actually a rescaling factor which is introduced for a consistent comparison with the experimental limits which customarily assume $f_p=f_n$ [215]. This assumption is justified in the case of the spin-0 mediator since $f_p$ and $f_n$ differ only by the contributions of up and down quarks which are sub-dominant with respect to the contribution from the strange quark (and then from the heavy quarks, see Eq. (35)), which is the same for proton and neutron. In the following numerical estimates we will then automatically set $\left[ f_p \frac{Z}{A}+f_n \left( 1-\frac{Z}{A}\right) \right]$ $\rightarrow f_p \sim 0.3$. As will be shown in the next section, for spin-1 mediators one expects in general $f_p \ne f_n$. This often translates into an enhancement of the cross-section and, hence, stronger limits on the model parameters. This can be already noticed by comparing the limits in the case of the SM-Higgs and *Z*-boson portals.

Current limits exclude then low values for both the mass of the DM and the one of the mediator. These limits will become, of course, progressively stronger, in case of absence of signals at XENON1T and/or LZ.

Concerning the DM relic density for $m_\chi < m_S,m_t$ the DM annihilation cross-section is suppressed by Yukawa structure of the couplings so that the correct relic density is obtained only around the resonance region $m_\chi \sim m_S/2$. This corresponds to the wide region between the two lines in Fig. 9. This region is of course determined by $\lambda _\chi ^S \times c_{S}$ and is then the same as shown in the left and right plots of Fig. 9. The difference between these two figures is the disappearance of the region corresponding to the *SS* final state (see right plot) where, annihilation cross-section is proportional to $(\lambda _\chi ^S)^4$ (see Eq. (37)). This channel is then not sufficient to avoid an over-density of the Universe for $\lambda _\chi ^S=0.25$. In the middle plot, the pole region is enlarged, to the point of covering almost all the parameter space, even joining the *SS* final state at $m_S \simeq 1$ TeV. For higher DM masses, instead, the correct relic density is achieved also far from s-channel resonances through either the $\overline{t} t$ channel or the *SS* channel, whether kinematically open. In such a case, the following analytical estimates, through the conventional velocity expansion, of the DM annihilation cross-section, can be obtained:

36 $$\begin{aligned} \langle \sigma v \rangle (\chi \chi \rightarrow \overline{t} t)\approx & {} \frac{3}{16 \pi } {(\lambda _\chi ^S)}^2 c_S^2 \frac{m_t^2}{v_h^2}\frac{1}{m_S^2} \nonumber \\\approx & {} 3.4 \times 10^{-25}{\text{ cm }}^3\,{\text{ s }}^{-1}{(\lambda _\chi ^S)}^2 c_S^2 \nonumber \\&\times \, {\left( \frac{1~\text{ TeV }}{m_S}\right) }^2 \quad \mathrm{for}\ m_t< m_\chi < m_S,\nonumber \\ \end{aligned}$$

**Fig. 10:**
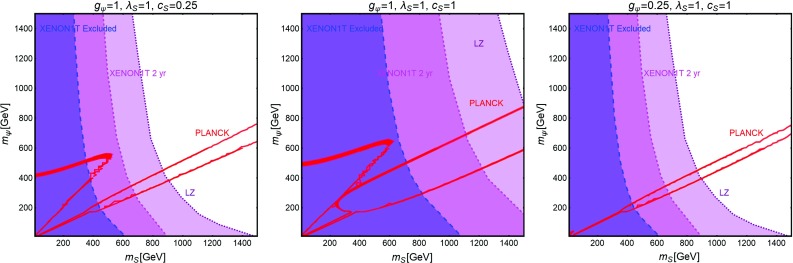
The same as Fig. 9 but for a Dirac fermion DM, i.e., replacing $\lambda ^S_\chi , \,m_\chi$ by $g_\psi ,\,m_\psi$, respectively

and:

37 $$\begin{aligned} \langle \sigma v \rangle (\chi \chi \rightarrow \overline{t} t)\approx & {} \frac{3}{64 \pi } {(\lambda _\chi ^S)}^2 c_S^2 \frac{m_t^2}{v_h^2}\frac{m_S^2}{m_\chi ^4} \nonumber \\\approx & {} 1.2 \times 10^{-26}{\text{ cm }}^3\, {\text{ s }}^{-1} {(\lambda _\chi ^S)}^2 c_S^2\nonumber \\&\times {\left( \frac{2~\text{ TeV }}{m_\chi }\right) }^4 {\left( \frac{m_S}{1.5~\text{ TeV }}\right) }^2,\nonumber \\ \langle \sigma v \rangle (\chi \chi \rightarrow S S)\approx & {} \frac{{(\lambda _\chi ^S)}^4}{64 \pi m_\chi ^2} \nonumber \\\approx & {} 5.8 \times 10^{-26}{\text{ cm }}^3\, {\text{ s }}^{-1}{(\lambda _\chi ^S)}^4\nonumber \\&\times {\left( \frac{1~\text{ TeV }}{m_\chi }\right) }^2 \quad \mathrm{for}\ m_t< m_S < m_\chi .\nonumber \\ \end{aligned}$$

As evident that both the $\overline{t} t$ and *SS* cross-sections are s-wave dominated and thus, velocity independent. As a consequence residual annihilation would occur at present times which can be probed by DM ID strategies. Similar to the case of the SM-Higgs portal, DD limits are much more competitive with respect to the ones from ID, hence the latter have not been explicitly exhibited on Fig. 9. We also notice that the dominant contribution of the annihilation cross-section into *SS* depends only on the $\lambda _\chi ^S$ coupling; as a consequence the scalar self-coupling $\lambda _S$ does not play a relevant role for DM phenomenology.^17^

#### Fermionic dark matter

The interaction of a fermionic DM and a scalar s-channel mediator can be described by the following phenomenological Lagrangian:

38 $$\begin{aligned} {\mathscr {L}}=-\xi g_\psi \overline{\psi }\psi S-\frac{c_S}{\sqrt{2}} \frac{m_f}{v_h}\overline{f} f S+{\mathscr {L}}_S, \end{aligned}$$

where ${\mathscr {L}}_S$ is defined in Eq. (33). Contrary to the case of a scalar DM, the operator $\overline{\psi }\psi S$ is of dimension 4, so that $g_\psi$ is already a dimensionless parameter. Note that similar to Eq. (32) we have parametrized $g_\psi$ to contain all the information of the $\overline{\psi }\psi S$ vertex including a normalization factor. One could think that an eventual VEV of the scalar mediator *S* can be the origin of the DM mass, so that $g_\psi \sim m_\psi /v_S$, with $v_S$ being the VEV of *S* [216–219]. We won’t make this assumption in this work and regard $g_\psi$ as a generic dimensionless constant.

The main results of our analysis have been summarized in Fig. 10. We have once again considered the DM and scalar masses as free parameters and an analogous assignation of the couplings as in the previous subsection.

The results shown in the figure can be described analytically as follows: the DD of the DM is again principally determined by SI interactions whose cross-section is given by:

39 $$\begin{aligned} \sigma ^\mathrm{SI}_{\psi p}&=\frac{\mu _{\psi p}^2}{\pi }g_\psi ^2 c_S^2 \frac{m_p^2}{v_h^2}f_N^2 \frac{1}{m_S^4}\approx \frac{1}{\pi }g_\psi ^2 c_S^2 \frac{m_p^{4}}{v_h^2}f_N^2\frac{1}{m_S^4} \nonumber \\&\approx 2.9 \times 10^{-45}\, {\text{ cm }}^{3} ~{\text{ s }}^{-1} g_\psi ^2 c_S^2 {\left( \frac{500~\text{ GeV }}{m_S}\right) }^4, \end{aligned}$$

where $\mu _{\psi p}=m_\psi m_p/(m_\psi +m_p)$ denotes reduced mass of the associated WIMP-proton system.

As evidenced from Fig. 10, DM masses even above the TeV scale, are excluded by current DD limits for $m_S \lesssim 400-500\,\text{ GeV }$. Values below the TeV scale for both the DM and mediator masses will be excluded in the absence of signals from the next generation experiments.

**Fig. 11:**
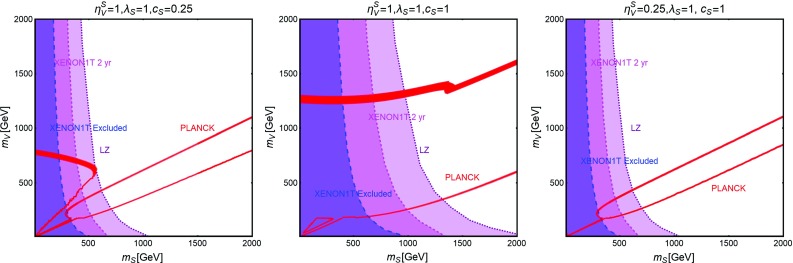
The same as Fig. 9 for a vector DM with scalar mediator, i.e., trading $\lambda ^S_\chi ,\,m_\chi$ with $\eta ^S_V,\,m_V$, respectively

The correct DM relic density can be achieved, without relying on s-channel resonances, only when at least one between the $\overline{t} t$ and *SS* final states is kinematically accessible. In such a case the DM pair annihilation cross-section can be approximated as:

40 $$\begin{aligned} \langle \sigma v \rangle (\overline{\psi }\psi \rightarrow \overline{t} t)~\approx & {} \frac{3}{4\pi }g_\psi ^2 c_S^2 \frac{m_t^2}{v_h^2}\frac{m_\psi ^2}{m_S^4}v^2, \nonumber \\\approx & {} 1.5 \times 10^{-26}\, {\text{ cm }^3}\,{\text{ s }}^{-1} g_\psi ^2 c_S^2 \nonumber \\&\times \, {\left( \frac{m_\psi }{300~\text{ GeV }}\right) }^2 {\left( \frac{1~\text{ TeV }}{m_S}\right) }^4\nonumber \\&\quad \mathrm{for}\ m_t< m_\psi < m_S, \end{aligned}$$

41 $$\begin{aligned} \langle \sigma v \rangle (\overline{\psi }\psi \rightarrow \overline{t} t)\approx & {} \frac{3}{64\pi }g_\psi ^2 c_S^2 \frac{m_t^2}{v_h^2}\frac{1}{m_\psi ^2}v^2, \nonumber \\\approx & {} 2.8 \times 10^{-26}\, {\text{ cm }^3}\,{\text{ s }}^{-1} g_\psi ^2 c_S^2\nonumber \\&\times \, {\left( \frac{600~\text{ GeV }}{m_\psi }\right) }^2 ,\nonumber \\ \langle \sigma v \rangle (\overline{\psi }\psi \rightarrow S S)\approx & {} \frac{3}{64\pi }g_\psi ^4 \frac{1}{m_\psi ^2}v^2, \nonumber \\\approx & {} 2.0 \times 10^{-26} \,{\text{ cm }^3}\,{\text{ s }}^{-1} g_\psi ^4 \nonumber \\&\times \,{\left( \frac{1~\text{ TeV }}{m_\psi }\right) }^2 \quad \mathrm{for}\ m_\psi > m_t,m_S.\nonumber \\ \end{aligned}$$

Here $v^2 \sim 0.23$. We notice again that in the limit $m_\psi \gg m_S$, the scalar self-coupling $\lambda _S$ does not influence the DM relic density. The dependence on the couplings between the three plots of Fig. 10 is the same as of the scalar case. However contrary to the case of scalar DM, all the annihilation channels are now velocity suppressed, hence cannot account for a sizable ID signals.

#### Vector dark matter

For the description of the vectorial DM case we consider the following Lagrangian:

42 $$\begin{aligned} {\mathscr {L}}= & {} \frac{1}{2} m_V \eta _V^S V^\mu V_\mu S+ \frac{1}{8}{(\eta _V^{S})}^2 V^\mu V_\mu S S \nonumber \\&-\, \frac{c_S}{\sqrt{2}} \frac{m_f}{v_h}S \overline{f} f+{\mathscr {L}}_S, \end{aligned}$$

which is inspired by the construction proposed in Refs. [220, 221] (see also [222]). Note that the first three terms of Eq. (42) appear after the spontaneous symmetry breaking, once the portal field is expanded as $(S+v_S)/\sqrt{2}$ with $v_S$ as the concerned VEV. The quantity $m_V$ is expressed as $\eta ^S_V v_S/2$.

A similar construction is also possible from a gauge invariant $D^\mu \mathbf{S} {(D_\mu \mathbf{S})}^*$ operator for a complex scalar field $\mathbf{S}$ with $D_\mu =\partial _\mu -i \frac{1}{2} \eta ^S_V V_\mu$. However, in this scenario the third term of Eq. (42) would require new BSM charges for the SM fermions.

This scenario has been analyzed with the same procedure as the scalar and fermionic DM cases. The results, reported in Fig. 11 appear not to be very different from what obtained in the case of a scalar DM in Fig. 9. This can be explained by the fact that a massive vectorial DM can be viewed as three scalar degrees of freedom. The DM scattering rate on protons and its most relevant annihilation channels are described by the following analytical expressions:

43 $$\begin{aligned} \sigma _{Vp}^\mathrm{SI}&=\frac{\mu _{Vp}^2}{4\pi } {(\eta _V^S)}^2 c_S^2 \frac{m_p^2}{v_h^2}f_p^2 \frac{1}{m_S^4},\nonumber \\&\approx 8.2 \times 10^{-45}\,{\text{ cm }}^2 {(\eta _V^S)}^2 c_S^2 {\left( \frac{1~\text{ TeV }}{m_S}\right) }^4. \end{aligned}$$

The parameter $\mu _{V p}$ $=m_V m_p$/$(m_V+m_p)$ as usual represents reduced mass of the relevant WIMP-proton system and

44 $$\begin{aligned} \langle \sigma v \rangle (V V \rightarrow \overline{t} t)\approx & {} \frac{1}{4\pi } {(\eta _{V}^S)}^2 c_S^2 \frac{m_t^2}{v_h^2}\frac{m_V^2}{m_S^4}, \nonumber \\\approx & {} 4.1 \times 10^{-26} \,{\text{ cm }^3}\, {\text{ s }}^{-1} {(\eta _V^S)}^2 c_S^2\nonumber \\&\times \, {\left( \frac{m_V}{300~\text{ GeV }}\right) }^2 {\left( \frac{1~\text{ TeV }}{m_S}\right) }^4\nonumber \\&\quad \mathrm{if}\ {m_S} < m_V,\nonumber \\&\quad \mathrm{or}\ \approx \frac{1}{64\pi }{(\eta _V^S)}^2 c_S^2 \frac{m_t^2}{v_h^2}\frac{1}{m_V^2}, \nonumber \\\approx & {} 2.8 \times 10^{-26}\, {\text{ cm }^3} \,{\text{ s }}^{-1} {(\eta _V^S)}^2 c_S^2 \nonumber \\&\times \, {\left( \frac{1~\text{ TeV }}{m_V}\right) }^2\quad \mathrm{if}\ {m_S} > m_V. \end{aligned}$$

45 $$\begin{aligned} \langle \sigma v \rangle (V V \rightarrow S S)\approx & {} \frac{11}{2304\pi }{(\eta _V^S)}^4 \frac{1}{m_V^2}\nonumber \\\approx & {} 1.7 \times 10^{-26} {\text{ cm }^3}\, {\text{ s }}^{-1}\nonumber \\&\times \, {(\eta _V^S)}^4 {\left( \frac{1~\text{ TeV }}{m_V}\right) }^2. \end{aligned}$$

**Table 1:** Table of couplings between the SM fermions and a $Z'$ (see Eq. (46)) for the three different realizations of a $Z'$ portal. Here $s^2_W\equiv \sin ^2\theta _W$

	$V_u^{Z'}$	$A_u^{Z'}$	$V_d^{Z'}$	$A_d^{Z'}$	$V_e^{Z'}$	$A_e^{Z'}$	$V_\nu ^{Z'}$	$A_\nu ^{Z'}$
SSM	$\frac{1}{4}-\frac{2}{3} s^2_W$	$\frac{1}{4}$	$-\frac{1}{4}+\frac{1}{3} s^2_W$	$\frac{1}{4}$	$-\frac{1}{4}+ s^2_W$	$-\frac{1}{4}$	$\frac{1}{4}$	$\frac{1}{4}$
$E_{6_\chi }$	0	$-\frac{1}{2\sqrt{10}}$	$-\frac{1}{\sqrt{10}}$	$\frac{1}{2\sqrt{10}}$	$\frac{1}{\sqrt{10}}$	$\frac{1}{2\sqrt{10}}$	$\frac{3}{\sqrt{10}}$	$-\frac{1}{2\sqrt{10}}$
$E_{6_\psi }$	0	$-\frac{1}{2\sqrt{6}}$	0	$\frac{1}{2\sqrt{6}}$	0	$\frac{1}{2\sqrt{6}}$	$\frac{1}{4\sqrt{6}}$	$-\frac{1}{2\sqrt{6}}$

### Spin-1 portals

In this subsection we will analyze, in analogous fashion as the previous subsection, the case of a s-channel spin-1 mediator. Unlike the scalar case, this kind of scenario offers a much richer collider phenomenology since one could assume gauge-like interactions (contrary to Yukawa-like interactions for spin-0 mediators) of the mediator with the SM light quarks and leptons, leading to visible signals, implying stronger constraints [223–241]. As a consequence we will consider a wider mass range, for both DM and the spin-1 mediator, in our analysis.

New spin-1 s-channel mediators can be straightforwardly associated to gauge bosons of extra *U*(1) groups. Extra *U*(1) symmetries are particularly common in extensions of the SM and, in particular, in Grand Unified Theories (GUT). The new $Z'$ particles, i.e., the gauge bosons of these *U*(1) groups, can be coupled to the SM fermions either indirectly, through kinetic mixing [242–249] with the *Z*-boson, or directly in case the SM fermions have non-trivial charges under the new symmetry group (see e.g., Refs. [250–256]). We will focus, in this section, on this last case while the case of kinetic mixing will be reviewed later in the text. Scalar or fermionic DM can be easily embedded in this kind of construction since they can be assumed to be new states charged under the new *U*(1) but singlet with respect to the SM group. In such a case portal interactions simply arise from their covariant derivatives.^18^

**Fig. 12:**
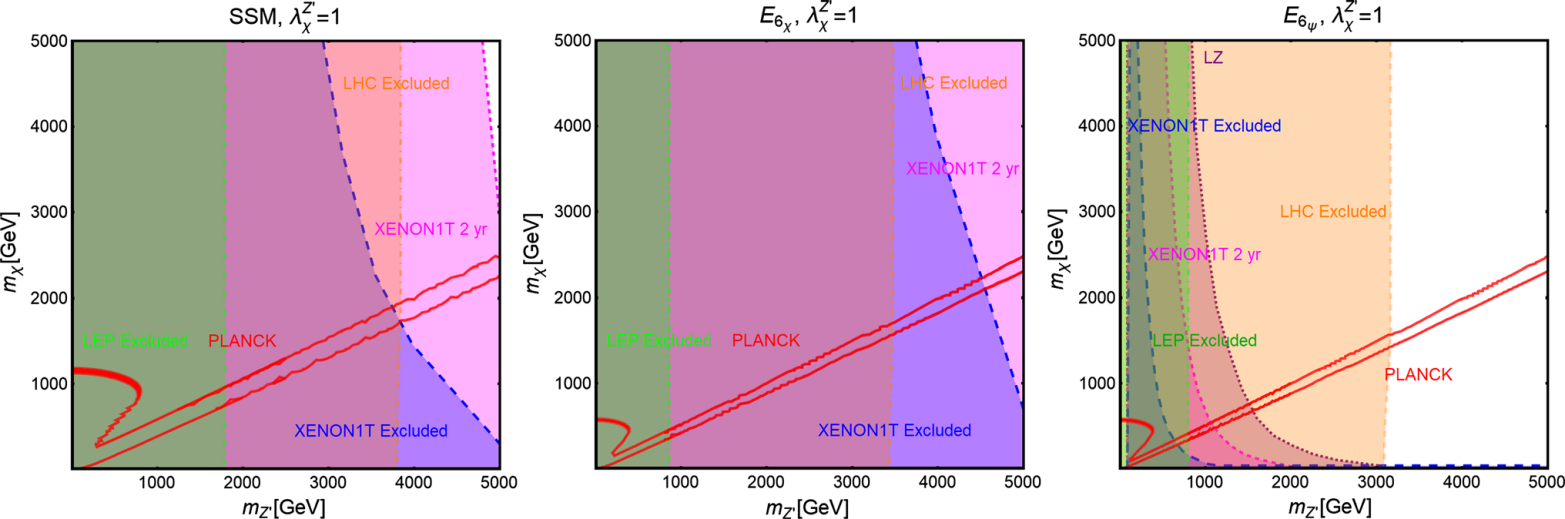
Summary of constraints for $Z'$ portal in the context of a scalar DM for the three different realizations (see Table 1), i.e., SSM (left), $E_{6_\chi }$ (middle) and $E_{6_\psi }$ (right) in the relevant bi-dimensional plane $m_{Z'},\,m_\chi$ for the choice of $\lambda ^{Z'}_\chi =1$. In these plots the red contours represent the correct DM relic density. The blue region is already excluded by DM DD while the magenta and purple regions are currently allowed but within the sensitivity of XENON1T (assuming 2 years of exposure) and LZ, respectively. Finally, the green and orange regions represent the exclusions from dilepton searches by the LEP/Tevatron and LHC experiments

In the case of spin-1 DM we re-propose the two constructions: the self-conjugate (Abelian) and not self-conjugate (non-Abelian) DM as already proposed in the SM Z-portal setup.

#### Scalar dark matter

Following the discussion above the interaction between a scalar DM and spin-1 ($Z'$) mediator, together with a piece connecting $Z'$ to the SM fermions, is described by the following Lagrangian:

46 $$\begin{aligned} {\mathscr {L}}= & {} i g' \lambda _\chi ^{Z'} \chi ^* \overset{\leftrightarrow }{\partial _\mu }\chi Z'^\mu + g{'^2} {(\lambda _\chi ^{Z'})}^2 {|\chi |}^2 Z'_\mu Z'^{\mu }\nonumber \\&+\,g' \sum _f \overline{f} \gamma ^\mu \left( V_f^{Z'}-A_f^{Z'} \gamma _5\right) f Z'_\mu . \end{aligned}$$

Notice that trilinear interaction between DM pairs and the $Z'$, of the form reported above, is possible only in the case of a complex scalar DM.

Similar to the case of scalar mediator, our main parameters will be represented by the DM and $Z'$ masses. For what regards the couplings of the $Z'$ with the SM fermions we will consider some definite assignations, as dictated by the Sequential Standard Model (SSM), i.e., same couplings as the *Z*-boson, and some GUT-inspired realizations. According to this the coupling $g'$ will be set to $g \approx 0.65$ in the case of SSM and to $g_\mathrm{GUT}=\sqrt{{5}/{3}}\,g \tan \theta _W \approx 0.46$ for the GUT realizations. Finally, unless differently stated, we will set $\lambda _\chi ^{Z'}=1$. The different assignations of the $V_f^{Z'}, A_f^{Z'}$ couplings (see Eq. (46)) considered in our analysis for the three cases of SSM, $E_{6_\chi }$ and $E_{6_\psi }$ realizations are exhibited in Table 1. For the same three realizations the effect of different constraints are summarized in Fig. 12.

As first thing we notice, in the case of SSM and $E_{6\chi }$ models, a much stronger impact of the limits from direct DM searches with respect to the case of scalar mediator. The reason lies on the fact that SI interactions, with cross-section given by (as usual for the case of SI we will refer to scattering on protons):

47 $$\begin{aligned} \sigma _{\chi p}^\mathrm{SI}= & {} \frac{\mu _{\chi p}^2}{\pi }\frac{g{'^4}}{m_{Z'}^4}\frac{{\left[ Z f_p+(A-Z) f_n \right] }^2}{A^2}, \nonumber \\ f_p= & {} 2 V_u^{Z'}+V_d^{Z'},f_n=V_u^{Z'}+2 V_d^{Z'}, \end{aligned}$$

are particularly efficient since, as evident from the fact that, for spin-1 mediators, the effective couplings of the DM with the proton and the neutron, $f_p$ and $f_n$, are just linear combination of couplings of the $Z'$ with up and down quarks.^19^ A further enhancement comes, in general, as already remarked, from the fact that $f_p \ne f_n$. As a consequence, an absence of signal from XENON1T would exclude values of the masses of the DM and of the $Z'$ even above 5 TeV.

Stringent limits from DD, although weaker with respect to the previous two cases, are remarkably present also for the $E_{6\psi }$ realization, despite the assignations of the charges of the quarks under the new *U*(1) imply a null vectorial combination. Indeed non-null vectorial couplings, at the typical energy scale of DM scattering with nucleons, are radiatively generated by the axial couplings of the $Z'$, in particular with the top quark [261–263]. An approximate expression, mostly valid for $m_{Z'}>m_Z$, for this Renormalization Group induced couplings are given by:

48 $$\begin{aligned} \widetilde{V}_u^{Z'}= & {} (3-8 s_W^2)\left[ \frac{\alpha _t}{2\pi } A_u^{Z'} \log \left( \frac{m_{Z'}}{m_Z}\right) \right. \nonumber \\&\left. -\, \left[ \frac{\alpha _b}{2 \pi } A_d^{Z'}+\frac{\alpha _\tau }{6\pi }A_e^{Z'}\right] \log \left( \frac{m_{Z'}}{\mu _N}\right) \right] , \nonumber \\ \widetilde{V}_d^{Z'}= & {} (3-4 s_W^2)\left[ -\frac{\alpha _t}{2\pi } A_u^{Z'} \log \left( \frac{m_{Z'}}{m_Z}\right) \right. \nonumber \\&\left. +\, \left[ \frac{\alpha _b}{2 \pi } A_d^{Z'}+\frac{\alpha _\tau }{6\pi }A_e^{Z'}\right] \log \left( \frac{m_{Z'}}{\mu _N}\right) \right] . \end{aligned}$$

Here $s_W\equiv \sin \theta _W$, $\alpha _{t,b,\tau }=y^2_{t,b,\tau }/4\pi$ with $y_f$ as the SM Yukawa couplings and $\mu _N$ is the characteristic energy scale of scattering interaction, here taken to be $1\,\text{ GeV }$.

The DM annihilation cross-section into SM fermions is instead velocity suppressed:

49 $$\begin{aligned} \langle \sigma v \rangle (\chi \chi ^{*} \rightarrow \overline{f} f) \approx \left\{ \begin{array}{l} g{'^4}{(\lambda _\chi ^{Z'})}^2 \frac{m_\chi ^2}{3 \pi m_{Z'}^4} v^2 \\ \quad \times \sum \limits _f n_c^f\left( {(V_f^{Z'})}^2 +{(A_f^{Z'})}^2 \right) \\ \qquad \mathrm{for}~ m_\chi < \frac{m_{Z'}}{2}, \\ g{'^4} {(\lambda _\chi ^{Z'})}^2 \frac{1}{48 \pi m_\chi ^2} v^2 \\ \quad \times \sum \limits _f n_c^f\left( {(V_f^{Z'})}^2 +({A_f^{Z'})}^2 \right) \\ \qquad \mathrm{for}~ m_\chi > \frac{m_{Z'}}{2}, \end{array} \right. \end{aligned}$$

**Fig. 13:**
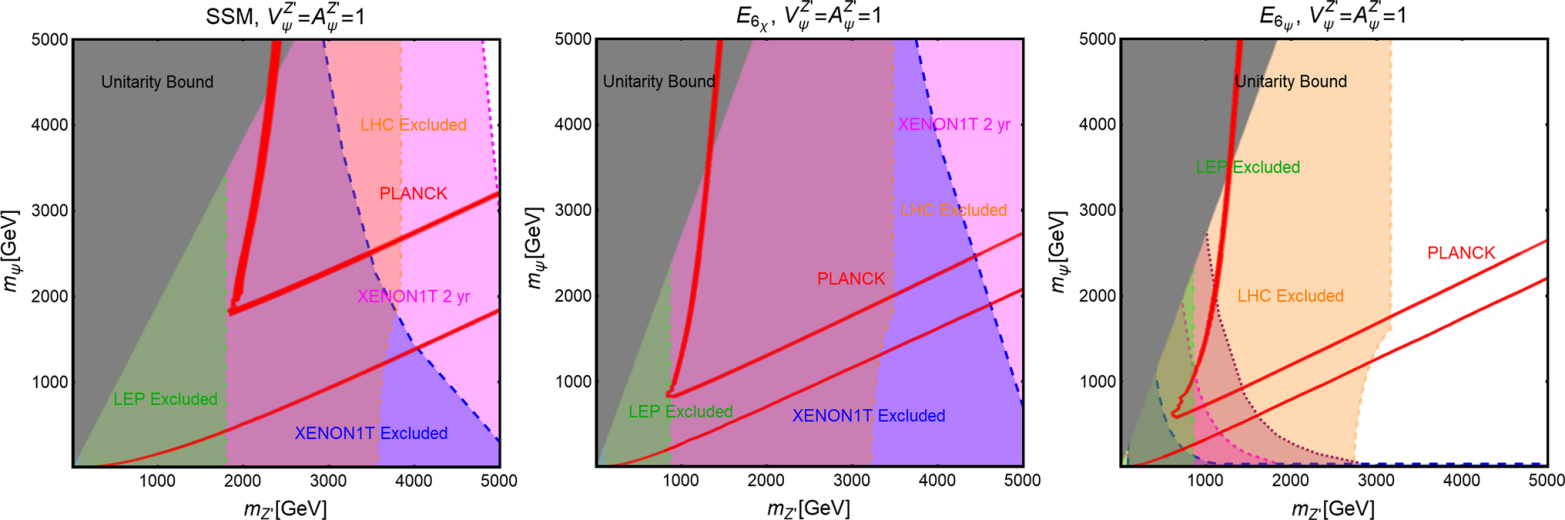
The same as Fig. 12 but for a Dirac fermion DM in the $m_{Z'},\,m_\psi$ bi-dimensional plane for the choice of $V^{Z'}_\psi =A^{Z'}_\psi =1$. The new grey region shows exclusion from the unitarity bound

**Fig. 14:**
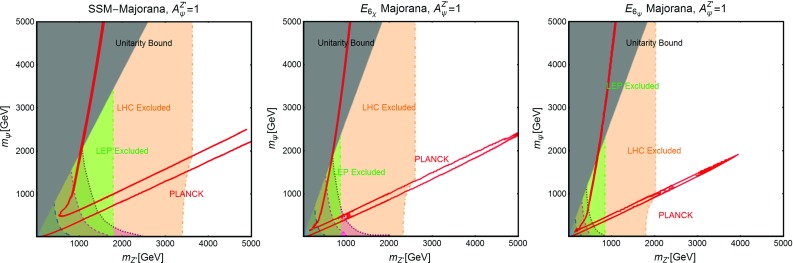
The same as Fig. 13 but for a Majorana fermion DM where $V^{Z'}_\psi =0$

where the sum runs over the kinematically accessible final states. The parameter $n^f_c$ represents color factor for the final state fermions. The correct relic density is thus achieved only around the pole region, namely $m_\chi \sim m_{Z'}/2$, unless the annihilation into $Z' Z'$ is kinematically accessible. This cross-section is s-wave dominated and can be simply approximated, for $m_\chi \gg m_{Z'}$ as:

50 $$\begin{aligned}&\langle \sigma v \rangle (\chi \chi ^{*} \rightarrow Z' Z') \approx \frac{g{'^4}{(\lambda _\chi ^{Z'})}^4}{8 \pi m_\chi ^2},\nonumber \\&\quad \approx 3.7 \times 10^{-26}\, {\text{ cm }^3}\,{\text{ s }}^{-1} \times {\left( \frac{1.5~\,\text{ TeV }}{m_\chi }\right) }^2, \end{aligned}$$

where, for definiteness, we have considered the SSM for the numerical estimates. As already pointed out that strong collider limits complement the ones from DD. The plots of Fig. 12, compared to the previous figures, report two new exclusion regions, green and orange. Both of these regions are related to limits associated to the couplings of the $Z'$ with SM leptons. The first ones, associated to the green regions, come from the LEP and Tevatron [264, 265] and are based on possible modifications of the dilepton production cross-section. Since they do not necessarily rely on on-shell production of the $Z'$, once its couplings with the SM fermions are fixed, like in our case, they are straightforwardly translated into lower bounds on $m_{Z'}$. More specifically, these lower bounds are $1789,\, 853$ and 804 GeV for the SSM, $E_{6_\chi }$ and $E_{6_\psi }$ configurations, respectively. These constraints are combined with the limits (orange regions in the plots) from LHC searches of dilepton resonances [138, 266, 267].

Contrary to the previous case, these limits are in principle sensitive to modification of the decay branching fraction of the $Z'$ as a consequence, for example, of couplings with the DM [139]. For the chosen assignation of the couplings, the decay branching fraction of the $Z'$ into DM pairs is small so that the limits substantially coincide with the ones reported by experimental collaborations. Other limits stemming from flavour and muon $(g-2)$ are weaker compared to the collider bounds [268–271].

#### Fermionic dark matter

As already mentioned we will describe the interactions of a fermionic DM $\psi$ and a $Z'$, mediating its interactions with the SM fermions, through a Lagrangian of the form:

51 $$\begin{aligned} {\mathscr {L}}= & {} g' \xi \overline{\psi } \gamma ^\mu \left( V_\psi ^{Z'}-A_\psi ^{Z'} \gamma _5\right) \psi Z'_\mu \nonumber \\&+\,g' \sum _f \overline{f} \gamma ^\mu \left( V_f^{Z'}-A_f^{Z'} \gamma _5\right) f Z'_\mu , \end{aligned}$$

where $\xi =1 (1/2)$ for Dirac (Majorana) fermions. We remind that in the case of Majorana fermions $V_\psi ^{Z'}=0$.

The combination of constraints is reported in Fig. 13 for a Dirac fermion DM, following the color coding of Fig. 12 (Fig. 14).

The SI cross-section, from t-channel exchange of a $Z'$, in the case of Dirac fermion DM, exactly coincides with the similar one for a complex scalar DM. As a consequence the excluded regions in the $(m_\psi ,m_{Z'})$ plane are the same as shown in the previous subsection.

In the case of Majorana DM, DD principally relies on SD interactions, to which Xenon based detectors are also sensitive. We have then reported, together with the most recent constraints [53, 272], an estimation of the XENON1T and LZ sensitivities. As evident, even in the case of LZ, we have much weaker limits, not competitive with bounds from dilepton searches.

On the contrary the regions corresponding to the correct DM relic density are sensitively different with respect to the case of scalar DM. Indeed the pair annihilation cross-section is not velocity suppressed and can be schematically expressed as:

52 $$\begin{aligned} \langle \sigma v \rangle (\overline{\psi }\psi \rightarrow \overline{f} f) \approx \left\{ \begin{array}{l} \frac{g'^4 m_\psi ^2}{\pi m_{Z'}^4} \times \sum \limits _{f} n_c^f \left( {(V_f^{Z'})}^2+{(A_f^{Z'})}^2\right) \\ \quad \times \left( {(V_\psi ^{Z'})}^2+{(A_\psi ^{Z'})}^2\right) \quad \mathrm{for}~ m_\psi < \frac{m_{Z'}}{2}, \\ \frac{g'^4}{16 \pi m_{\psi }^2} \sum \limits _f n_c^f \left( {(V_f^{Z'})}^2+{(A_f^{Z'})}^2\right) \\ \quad \times \left( ({V_\psi ^{Z'})}^2+{(A_\psi ^{Z'})}^2\right) \quad \mathrm{for}~ m_\psi > \frac{m_{Z'}}{2}. \end{array} \right. \end{aligned}$$

In addition, the t-channel mediated annihilation process $\overline{\psi }\psi \rightarrow Z' Z'$ is particularly efficient, where the corresponding cross-section is given by:

53 $$\begin{aligned}&\langle \sigma v \rangle (\overline{\psi }\psi \rightarrow Z' Z') \approx \frac{g'^4}{\pi m_{Z'}^2} \left( {(V_\psi ^{Z'})}^2 {(A_\psi ^{Z'})}^2\phantom {\frac{m_\psi ^2}{m_{Z'}^2}}\right. \nonumber \\&\quad \left. +\left( {(V_\psi ^{Z'})}^4+{(A_\psi ^{Z'})}^4\right) \frac{m_{Z'}^2}{2 m_\psi ^2}+\frac{v^2}{3}{(A_\psi ^{Z'})}^4 \frac{m_\psi ^2}{m_{Z'}^2}\right) . \end{aligned}$$

As evident that a strong enhancement is originated by the velocity dependent term being proportional to $\frac{m_\psi ^2}{m_{Z'}^2}$. It is well-known that this kind of behavior leads to a violation of perturbative unitarity unless new degrees of freedom, like a dark Higgs [168], are added to cure the pathological behavior of the theory. In absence of a UV completion, we have imposed, in our simplified framework, a unitarity constraint on the axial coupling $A_\psi ^{Z'}$ of the form:

54 $$\begin{aligned} A^{Z'}_\psi \le \frac{\pi m_{Z'}^2}{2 m_\psi ^2}. \end{aligned}$$

#### Vector dark matter

As stated already we will discuss separately the cases of Abelian (real vector) and non-Abelian (complex vector) DM, in order to exploit different scenarios regarding DD. Similar to the case of SM *Z*-portal, we will consider the following two constructions for non-Abelian and Abelian DM, respectively:

55 $$\begin{aligned} {\mathscr {L}}= & {} g' \eta ^{Z'}_V [[VVZ']]+g'\sum _f\overline{f}\gamma ^\mu \left( V^{Z'}_f-A^{Z'}_f \gamma ^5\right) f Z'_\mu , \end{aligned}$$

56 $$\begin{aligned} {\mathscr {L}}= & {} g' \eta ^{Z'}_V \epsilon ^{\mu \nu \rho \sigma } V_{\mu } Z'_{\nu } V_{\rho \sigma }\nonumber \\&+\,g'\sum _f\overline{f}\gamma ^\mu \left( V^{Z'}_f-A^{Z'}_f \gamma ^5\right) f Z'_\mu , \end{aligned}$$

where the second terms represent interactions among $Z'$ and the SM fermions. As a convention we have normalized, in both cases, the DM coupling to the new gauge coupling $g'$. As already discussed in the case of Abelian DM we have considered a Chern–Simons type interaction [210]. The interaction term of the complex vector DM can instead arise at the renormalizable level by considering the DM as vector boson of an additional non-Abelian group, the minimal option would be SU(2), and the exact mimic of the SM EW group $SU(2)_L \times U(1)_Y$ (this would require the presence of an additional $Z'$ which we assume to be heavy enough to have a negligible impact in the low-energy phenomenology). The parameter $\eta ^{Z'}_V$ contains the model specific information for $[[VVZ']]$ interaction.

The most important difference among the two scenarios relies in the DM-nucleon scattering cross-section. In the non-Abelian case, interaction with the vectorial current $\overline{q} \gamma ^\mu q$ are possible, thus leading to the SI cross-section:

57 $$\begin{aligned} \sigma ^\mathrm{SI}_{Vp}=\frac{g'^4 {(\eta _V^{Z'})}^2 \mu _{Vp}^{2} }{\pi m_{Z'}^4}{\left( V_u^{Z'} \left( 1+\frac{Z}{A}\right) + V_d^{Z'} \left( 2-\frac{Z}{A}\right) \right) }^2. \end{aligned}$$

In the Abelian case, on the contrary, the only (momentum) unsuppressed interaction, is with the axial-vector quark current $\overline{q} \gamma ^\mu \gamma _5 q$, so that the interaction with nucleons is SD with cross-section given by:

58 $$\begin{aligned} \sigma ^\mathrm{SD}_{Vn}= & {} \frac{3 g'^4 {(\eta _V^{Z'})}^2 \mu _{Vn}^2}{\pi m_{Z'}^4 (S_p^A+S_n^A)^2} \times \left( A_u^{Z'} \left( \varDelta _u^p S_p^A+\varDelta _d^p S_n^A\right) \right. \nonumber \\&\left. +\, A_d^{Z'} \left( \left( \varDelta _d^p+\varDelta _s^p\right) S_p^A +\left( \varDelta _u^p+\varDelta _s^p\right) S_n^A\right) \right) ^2. \end{aligned}$$

Here $\varDelta ^p_{i}$ denotes spin content of the ‘i’-th quark flavour inside proton and $S^A_p,\,S^A_n$ represent proton and neutron contribution to the spin of nucleus, respectively. For Xenon based detectors $S^A_p \ll S^A_n$ so that the reference cross-section is the one of DM on neutrons.

The Abelian and non-Abelian DM have very different properties also concerning annihilations. In the first case we see that the annihilation cross-section into SM fermions is strongly suppressed, being, in fact, given by:

59 $$\begin{aligned} \langle \sigma v \rangle (VV \rightarrow \overline{f} f)\approx & {} \frac{g'^4 {(\eta _V^{Z'})}^2 m_f^2}{9 \pi m_{Z'}^4}v^2 \sum _f n_c^f {(A_f^{Z'})}^2 \nonumber \\&+\,\frac{10}{81 \pi }g'^4 {(\eta _V^{Z'})}^2 \frac{m_V^2}{(m_{Z'}^2-4 m_V^2)^2}v^4 \nonumber \\&\times \, \sum _f n_c^f \left( {(V_f^{Z'})}^2+{(A_f^{Z'})}^2\right) . \end{aligned}$$

Its first non-zero contribution, the p-wave, is suppressed by the final state fermion mass. Ad exception of the case in which the mass of the ${Z'}$ is not too far from the one of the top quark, the DM annihilation cross-section is actually dominated by the *d*-wave contribution and hence, suppressed as $v^4$. On the contrary, the DM features a very efficient annihilation into $Z'$ pairs, when allowed kinematically:

60 $$\begin{aligned} \langle \sigma v \rangle (VV \rightarrow Z' Z') \approx \frac{1}{18 \pi } g'^4 {(\eta _V^{Z'})}^4 \frac{m_V^2}{m_{Z'}^4}. \end{aligned}$$

Its contribution to the DM relic density is nevertheless limited by the unitarity constraint [273, 274].

In the case of non-Abelian vector DM the annihilation cross-section into SM fermions is only p-wave suppressed:

61 $$\begin{aligned} \langle \sigma v \rangle (VV \rightarrow \overline{f} f)\approx & {} \frac{2 g'^4 {(\eta _V^{Z'})}^2 }{\pi }v^2 \frac{m_V^2}{(m_{Z'}^2-4 m_V^2)^2}\nonumber \\&\times \sum _f n_c^f \left( {(V_f^{Z'})}^2+{(A_f^{Z'})}^2\right) , \end{aligned}$$

while the annihilation cross-section into $Z' Z'$, instead, features a similar behavior with respect to the Abelian case:

62 $$\begin{aligned} \langle \sigma v \rangle (VV \rightarrow Z' Z') \approx \frac{1}{4 \pi } g'^4 {(\eta _V^{Z'})}^4 \frac{m_V^2}{m_{Z'}^4}. \end{aligned}$$

**Fig. 15:**
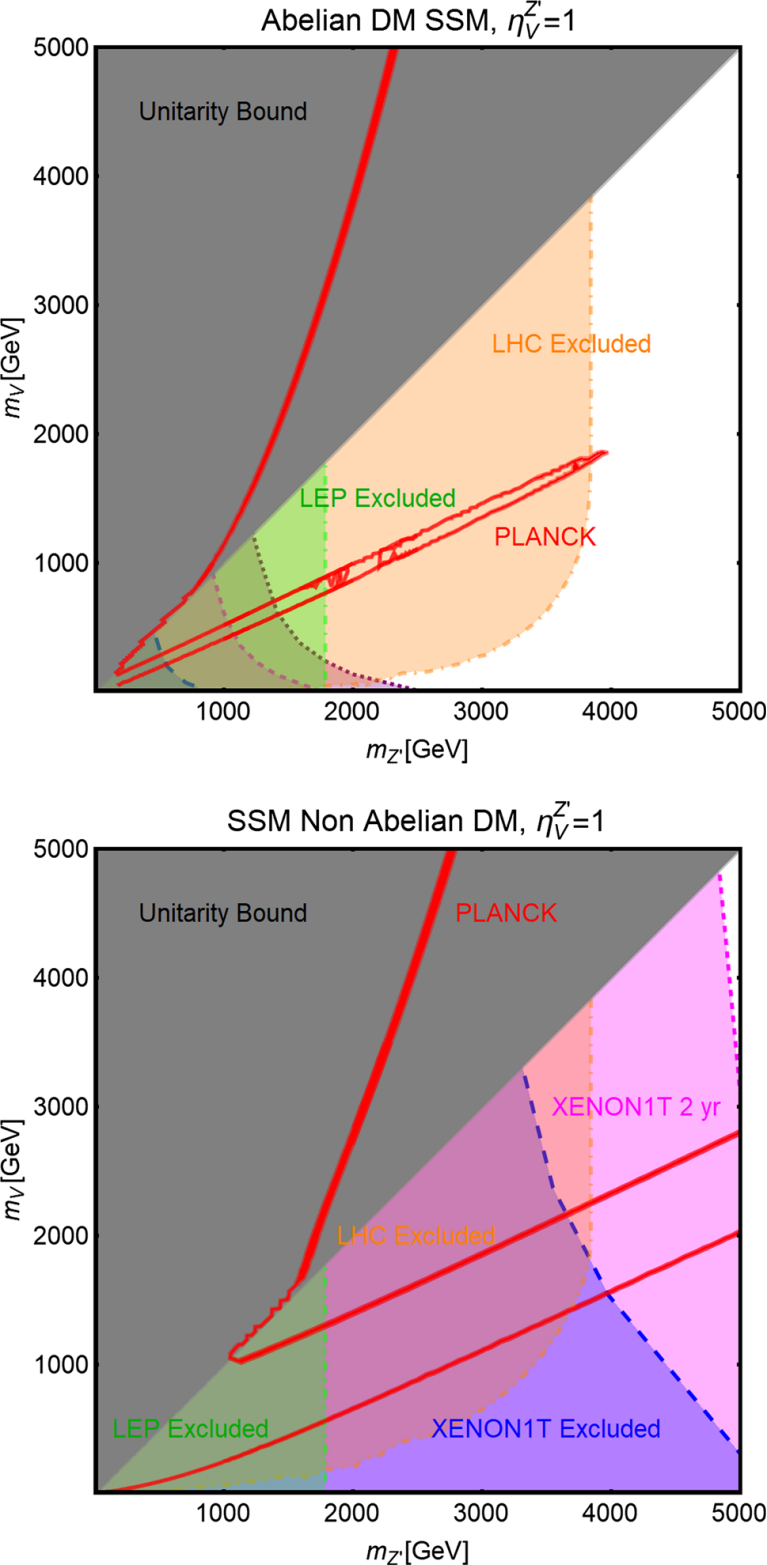
Combined constraints in the $m_{Z'},\,m_V$ bi-dimensional plane for Abelian (top panel) and non-Abelian (bottom panel) vectorial DM interacting with a $Z'$ mediator, for the choice of $\eta ^{Z'}_V=1$. In both cases we have chosen SSM couplings of the $Z'$ with the SM fermions (see Table 1). Colour scheme is the same as Fig. 13

The impact of different theoretical and experimental limits on the two scenarios are shown, as customary, in the plane $(m_{Z'},m_V)$, in Fig. 15. In the case of non-Abelian DM the weaker limits from DD do not coincide with a larger viable region for thermal DM since the contemporary suppression of the DM annihilation cross-section into fermions (the annihilation into $Z'Z'$ is strongly limited by unitarity) allows for the correct relic density only above the limit from LHC dilepton searches ad exception of a tiny region corresponding to the s-channel resonance. Much worse is the situation in the case of a non-Abelian vectorial DM. Indeed, already current limits from DD overpower the ones from LHC and exclude thermal DM for both $m_{Z'}$ and $m_V$ below 5 TeV.

## t-channel portals

We consider, in this section, the case in which the DM, instead of coupling in pairs, is coupled with one mediator state and a SM quark. Keeping the assumption that the DM is a SM singlet, the mediator field should carry at least non-trivial charges under the $SU(3)_C$ and $U(1)_Y$ of the SM.

We will then consider the following Lagrangians:^20^

63 $$\begin{aligned} {\mathscr {L}}= & {} -\lambda _{\varPsi _q} \overline{\varPsi }_q \chi q_R+h.c. ~~~~\mathrm{or}~~~~\nonumber \\ {\mathscr {L}}= & {} -\lambda _{\Sigma _q} \overline{\psi }\Sigma _q q_R+h.c.,\,\,\,\,q=u\, \text{ or } \,d \nonumber \\ {\mathscr {L}}= & {} -\lambda _{\varPsi _Q} \overline{\varPsi }_Q \chi q_L+h.c. ~~~~\mathrm{or}~~~~\nonumber \\ {\mathscr {L}}= & {} -\lambda _{\Sigma _Q} \overline{\psi }\Sigma _Q q_L+h.c. \end{aligned}$$

These Lagrangians describe the couplings of a fermionic (scalar) DM $\psi$ ($\chi$) with scalar (fermion) mediator fields $\Sigma _u$, $\Sigma _d$, $\Sigma _Q$, $(\varPsi _{u}$, $\varPsi _{d}$, $\varPsi _{Q})$ having quantum numbers:

64 $$\begin{aligned} (3,2,-1/6),\,\,\,\,\, (3,1,-1/3),\,\,\,\,\,(3,1,2/3), \end{aligned}$$

with respect to $SU(3)_C,\,SU(2)_L,\,U(1)_Y$. Hence, these mediators can interact with the left-handed quark doublet, up-type and down-type right-handed quarks,^21^ respectively.

To ensure the cosmological stability of the DM some suitable additional quantum numbers for both the DM and mediator should be assumed, so that couplings between any of these two states and only SM states are forbidden.^22^ It is also evident that the DM can be stable only if it is lighter than the t-channel mediator. In order to avoid possible occurrence of flavour violating effects, with consequent strong constraints on the couplings $\lambda _{\varPsi _{u,d,Q}} (\lambda _{\Sigma _{u,d,Q}})$, we assume that the mediator carries also a flavour quantum number (a “flavored DM” [278–280] would be equally feasible), for example $\Sigma _{u}\equiv$ $(\sigma _u,\sigma _c,\sigma _t)$ $(\varPsi _{u}\equiv (\psi _u,\psi _c,\psi _t))$, and that the interactions of Eq. (63) are flavour conserving. This is achieved by assuming the components of the mediator field to be degenerate in masses. We call these masses $m_{\Sigma _{u,d,Q}}$ and $m_{\varPsi _{u,d,Q}}$ and the couplings $\lambda _{\varPsi _{u,d,Q}},\,\lambda _{\Sigma _{u,d,Q}}$ (being actually matrices) and considered them to be diagonal in the flavour space. For further simplification, we will assume all of these couplings to be equal and thus, drop the flavour indices.

Concerning DM phenomenology the relic density is determined, for all the three types of candidates, by annihilation processes into fermion pairs induced by t-channel exchange of the mediator field. For close values of the DM and mediator masses co-annihilation processes, like $\psi \Sigma _{u,d,Q}$ $(\varPsi _{u,d,Q} \chi )$ $\rightarrow (u,d,Q) g$ and mediator pair annihilation processes like, $\Sigma _{u,d,Q} \Sigma _{u,d,Q}$ $(\varPsi _{u,d,Q} \varPsi _{u,d,Q})$ $\rightarrow \overline{u}u, \overline{d}d,\overline{Q}Q, gg$ induced by gauge interactions, might also become important. In analogous fashion, as the other models reviewed in this work, we have focused on the assignations $\lambda _{\varPsi _{u,d,Q}}$, $\lambda _{\Sigma _{u,d,Q}}$ $=1$. In such a case the dominant contribution to the DM relic density comes from the DM pair annihilations into SM fermions. We can then achieve an analytical description through some simple approximations of the corresponding cross-sections, based on the velocity expansion (more complete expressions are presented in the Appendix):

65 $$\begin{aligned} \langle \sigma v \rangle ^\mathrm{complex\, scalar}= & {} \frac{3{(\lambda _{\varPsi _u})}^4 m_t^2}{16 \pi {\left( m_\chi ^2+m_{\varPsi _u}^2-m_t^2\right) }^2}\nonumber \\&\times \, {\left( 1-\frac{m_t^2}{m_\chi ^2}\right) }^{3/2} +\frac{3{(\lambda _{\varPsi _\mu })}^4 m_\chi ^2 v^2}{8 \pi {(m_{\varPsi _u}^2+m_\chi ^2)}^2}, \nonumber \\ \langle \sigma v \rangle ^\mathrm{Majorana~fermion}= & {} \frac{3{(\lambda _{\Sigma _u})}^4 m_t^2}{32 \pi {\left( m_\psi ^2+m_{\Sigma _u}^2-m_t^2\right) }^2}\nonumber \\&\times \,\sqrt{\left( 1-\frac{m_t^2}{m_\psi ^2}\right) } +\frac{3{(\lambda _{\Sigma _\mu })}^4 m_\psi ^2 v^2}{8 \pi {(m_{\Sigma _u}^2+m_\psi ^2)}^2}, \nonumber \\ \langle \sigma v \rangle ^\mathrm{Dirac~fermion}= & {} \sum _{f=u,c,t}~~ \frac{3{(\lambda _{\Sigma _u})}^4 m_\psi ^2}{32 \pi {\left( m_\psi ^2+m_{\Sigma _u}^2-m_f^2\right) }^2}\nonumber \\&\times \,\sqrt{1-\frac{m_f^2}{m_\psi ^2}}. \end{aligned}$$

In the cases of complex scalar and Majorana fermion DM the s-wave term of the annihilation cross-section is helicity suppressed so that, ad exception of values of the DM mass close to the top-quark mass, the dominant contributions comes from the p-wave term, leading to the following estimate:

66 $$\begin{aligned} \langle \sigma v \rangle\approx & {} 1.7\times 10^{-26} {\text{ cm }}^3 {\text{ s }}^{-1} {(\lambda _{\Sigma _u})}^4\nonumber \\&\times \, {\left( \frac{m_\psi }{200\,\text{ GeV }}\right) }^2 {\left( \frac{1\,\text{ TeV }}{m_{\Sigma _u}}\right) }^4. \end{aligned}$$

On the contrary, the annihilation cross-section of a Dirac fermion DM is s-wave dominated and can be estimated as:

67 $$\begin{aligned} \langle \sigma v \rangle= & {} 4.2\times 10^{-26} {\text{ cm }}^3 {\text{ s }}^{-1} {(\lambda _{\Sigma _u})}^4 \nonumber \\&\times \,{\left( \frac{m_\psi }{200\,\text{ GeV }}\right) }^2 {\left( \frac{1\,\text{ TeV }}{m_{\Sigma _u}}\right) }^4. \end{aligned}$$

For simplicity we have referred, for analytical expression and numerical estimates, to the case of $\Sigma _u (\varPsi _u)$ mediator. The extension to the other cases is straightforward; as can be easily noticed that the main difference is for the case in which the DM and the mediator are coupled with right-handed down-type quarks and the terms dependent on the top-quark mass are absent.

Concerning DD, it relies on the scattering of the DM with up-quarks through s-channel exchange of the mediator. In the cases of complex scalar and Dirac fermion DM these interactions lead to SI cross-sections (in the case of a Dirac fermion DM also SD scattering is present but its impact is negligible given the much weaker experimental limits) whose expressions are [61, 69, 140]:

68 $$\begin{aligned} \sigma _{\chi p}^\mathrm{SI}= & {} \left\{ \begin{array}{l} \frac{{(\lambda _{\varPsi _u})}^2 m_p^2}{32 \pi {\left( m_{\varPsi _u}^2-m_\chi ^2\right) }^2}{\left[ 1+\frac{Z}{A}\right] }^2,\\ \\ \frac{{(\lambda _{\varPsi _d})}^2 m_p^2}{32 \pi {\left( m_{\varPsi _d}^2-m_\chi ^2\right) }^2}{\left[ 2-\frac{Z}{A}\right] }^2,\\ \\ \frac{{9(\lambda _{\varPsi _Q})}^2 m_p^2}{32 \pi {\left( m_{\varPsi _Q}^2-m_\chi ^2\right) }^2}, \end{array} \right. \quad \text{(complex } \text{ scalar) },\nonumber \\ \sigma _{\psi p}^\mathrm{SI}= & {} \left\{ \begin{array}{l} \frac{{(\lambda _{\Sigma _u})}^2 m^2_p}{64 \pi {\left( m_{\Sigma _u}^2-m_\psi ^2\right) }^2}{\left[ 1 +\frac{Z}{A}\right] }^2,\\ \\ \frac{{(\lambda _{\Sigma _d})}^2 m^2_p}{64 \pi {\left( m_{\Sigma _d}^2-m_\psi ^2\right) }^2}{\left[ 2 -\frac{Z}{A}\right] }^2,\\ \frac{{3(\lambda _{\Sigma _Q})}^2 m^2_p}{64 \pi {\left( m_{\Sigma _Q}^2-m_\psi ^2\right) }^2}. \end{array} \right. \quad \text{(Dirac } \text{ fermion) }, \end{aligned}$$

On the contrary, in the case of a Majorana fermion DM, one should consider SD interactions described by the following cross-section:

69 $$\begin{aligned} \sigma _{\psi n}^\mathrm{SD}= \left\{ \begin{array}{l} \frac{3 {(\lambda _{\Sigma _u})}^2 \mu _{\psi n}^2 {(\varDelta ^n_u)}^2}{16 \pi {\left( m_{\Sigma _u}^2-m_\psi ^2\right) }^2},\\ \\ \frac{3 {(\lambda _{\Sigma _d})}^2 \mu _{\psi n}^2 (\varDelta ^n_d+\varDelta ^n_s)^2}{16 \pi {\left( m_{\Sigma _d}^2-m_\psi ^2\right) }^2},\\ \\ \frac{3 {(\lambda _{\Sigma _Q})}^2 \mu _{\psi n}^2 (\varDelta ^n_u+\varDelta ^n_d+\varDelta ^n_s)^2}{16 \pi {\left( m_{\Sigma _Q}^2-m_\psi ^2\right) }^2}, \end{array} \right. \end{aligned}$$

here $\mu _{\psi n}$ represents reduced mass of the WIMP-neutron system and $\varDelta ^n_u,\,\varDelta ^n_d,\,\varDelta ^n_s$ denote spin content of the up, down and strange quark inside neutron.

**Fig. 16:**
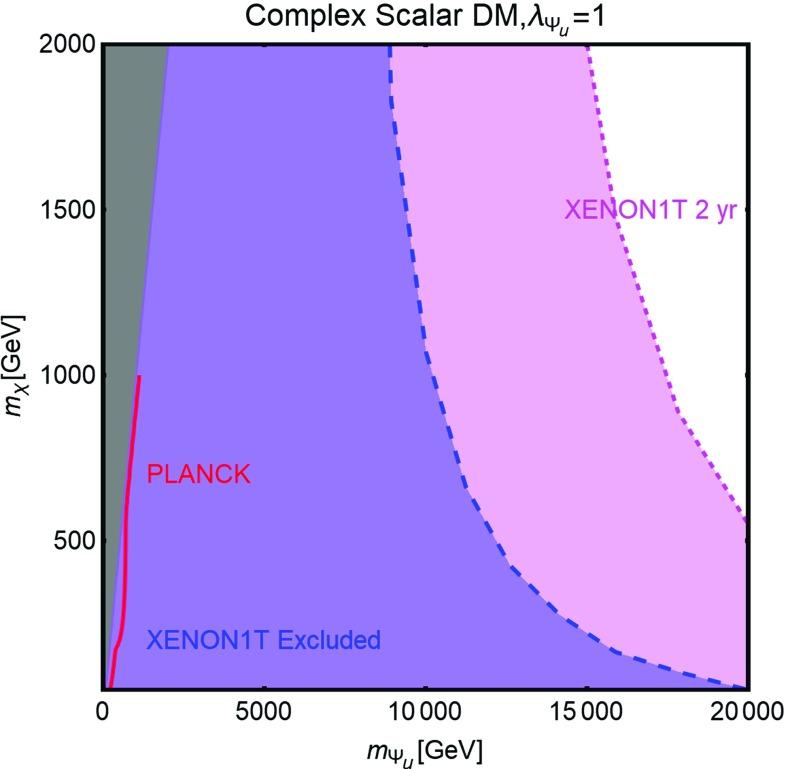
Combined constraints for a complex scalar DM $\chi$ coupled with the right-chiral up-type quarks through a Dirac fermionic t-channel mediator $\varPsi _u$. The results are in the bi-dimensional plane $(m_{\varPsi _u},m_\chi )$ and the coupling $\lambda _{\varPsi _u}$ has been set to 1. The red curve corresponds to the contour of correct DM relic density. The blue region is excluded by the current constraints while the magenta region will be excluded in the absence of signals from XENON1T after 2 years of exposure. The grey region corresponds to $m_\chi > m_{\varPsi _u}$ where the DM is not cosmologically stable

**Fig. 17:**
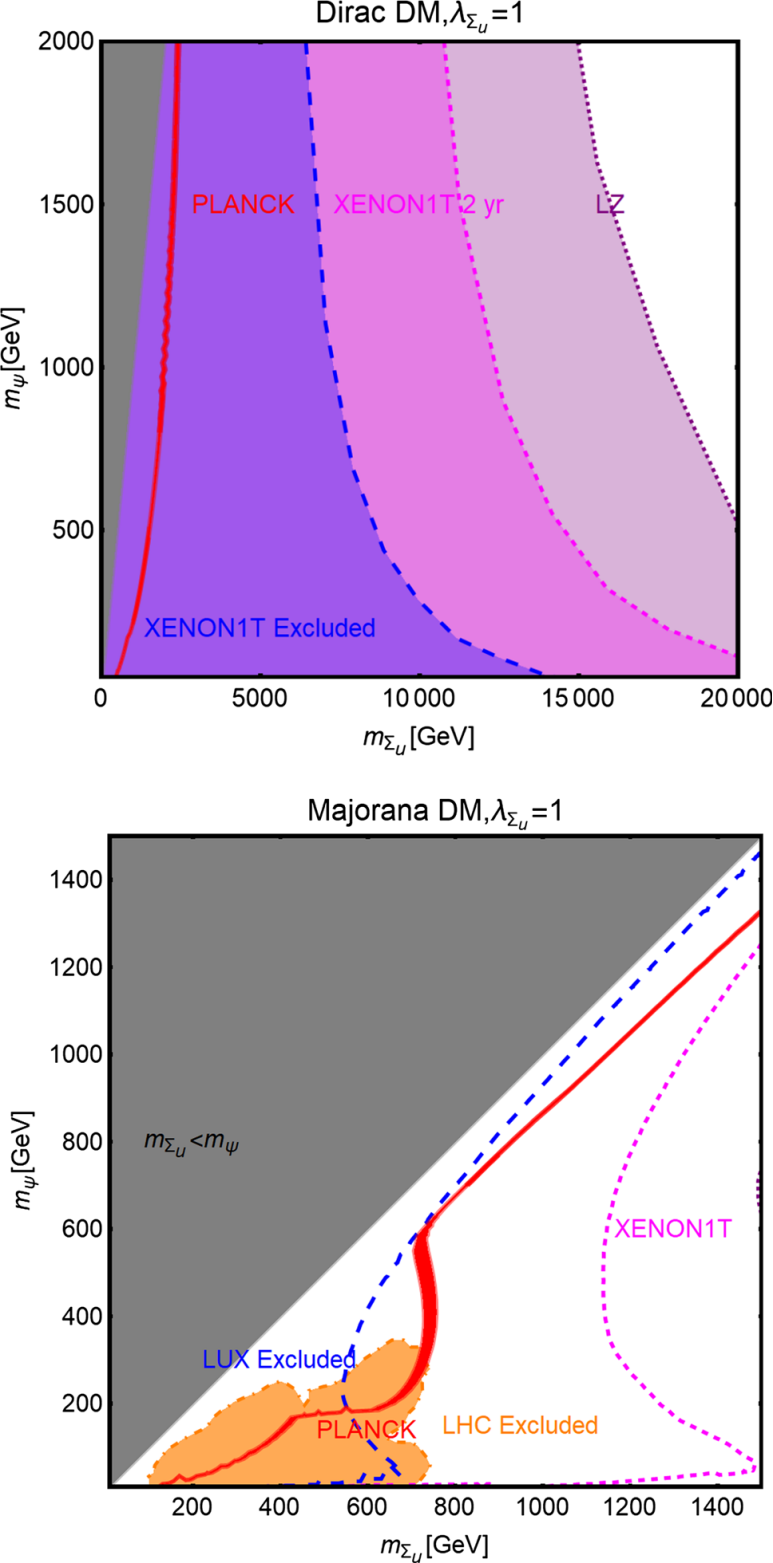
The same as Fig. 16 but in the $(m_{\Sigma _u},m_\psi )$ bi-dimensional plane for the case of a Dirac (top panel) or Majorana (bottom panel) fermion DM and scalar t-channel mediator $\Sigma _u$. The coupling $\lambda _{\Sigma _u}$ has been set to 1. For the Majorana fermion DM case, unlike the Dirac one, only boundaries of DD limits are shown. In the case of Majorana fermion DM we have also reported the excluded region (orange) by combined LHC limits from monojet and dijet+missing energy events [140, 284]. Similar limits for the cases of Dirac fermion and complex scalar DM have been omitted since they are irrelevant with respect to DD exclusions

The results of our analysis are presented, in the usual fashion, in Figs. 16 and 17 for scalar and fermionic (both Dirac and Majorana) DM, respectively.

For simplicity we will refer only to the case in which the DM interacts only with one kind of mediator, chosen to be $\Sigma _u (\varPsi _u)$. As evidenced by the expressions above, differences between the various cases mostly arise in the scattering cross-section because of the different interactions with the light-quarks, leading in turn to different interactions with proton and neutrons. This however does not lead to dramatic variations in the picture presented in Figs. 16 and 17. Similarly, there is not loss of generality in restricting to the hypothesis of a single mediator field. Adding different mediators would lead to simultaneous enhancement of the DM annihilation and scattering cross-section causing a global shift of the iso-contours in the bi-dimensional planes of the figures. There is a potential exception though. In the case when both $\Sigma _Q$ and at least one between $\Sigma _u$ and $\Sigma _d$ are considered and a mass mixing is allowed between these fields (this occurs for example in Supersymmetric theories), Majorana fermion DM would acquire a sizable SI scattering cross-section [281–283]. We will not explicitly discuss this kind of scenario.

Figures 16 and 17 clearly show that the cases of complex scalar and Dirac fermion DM, in which unsuppressed SI interactions are present, are already substantially ruled out.^23^

More interesting is the case of a Majorana DM. A sizable portion of the parameter space corresponding to the correct DM relic density survives from the present constraints but is within the reach, for masses of both the DM and the mediator up to a few TeV, of the near future DD experiments. Possible complementarity with LHC searches is also worth of being explored.

Collider searches can probe the DM pair production through mono-X events and pair production of the scalar or fermionic mediator with subsequent decay into a DM particle and a SM quark through dijet + missing energy events. The latter signature is also considered in supersymmetry (SUSY) searches since it would originate from squark pair production, with subsequent decay into a neutralino and a quark, under the assumption of decoupled gluino. Limits from SUSY searches cannot be straightforwardly applied to the scenario under consideration since the pair production cross-section of the scalar mediator field is enhanced by the presence of diagrams with t/u channel exchange of the DM (the corresponding diagrams in SUSY are suppressed since the concerned couplings of the neutralino with quarks and squarks cannot be of $\mathscr {O}(1)$). We have then applied the limits from the dedicated studies [140, 284] (see also [285, 286]), in which searches of both monojet and dijet + missing energy events have been considered, and reported them in the bottom panel of Fig. 17 (orange region with dashed orange border). Similar constraints have been also obtained in the case of Dirac and complex scalar DM. These are, however, overwhelmed by the constraints from DM DD and thus, omitted in the figures.

Our discussion can be extended further by considering mediators charged only under EW interactions while being colour singlet. Even if no interactions between the DM and the quarks are present at the tree-level, DD might still represent a more effective probe with respect to other DM searches.

A compelling case is represented by Dirac fermion DM where SI interaction can be induced at the loop level. The DM indeed interacts with the quarks through an effective vertex generated by a triangle loop formed by one scalar field and two SM leptons. The mediator of the interaction is mostly the photon. The corresponding scattering rate is given by [278, 287, 288]:

70 $$\begin{aligned} \frac{d\sigma _{\psi p}^\mathrm{SI}}{dE_R}= & {} \frac{2 m_p Z^2}{4\pi v_E^2}\frac{\lambda _{\Sigma _e}^4 \alpha ^2}{(16 \pi ^2)^2 m_{\Sigma _e}^4}\nonumber \\&\times \,{\left[ \sum _{l=e,\mu ,\tau } 1+\frac{2}{3}\log \left( \frac{m_l^2}{m_{\Sigma _e}^2}\right) \right] }^2 |F_\mathrm{em}(E_R)|^2,\nonumber \\ \end{aligned}$$

where $v_E$ is the DM velocity in the Earth frame, $E_R$ is the recoil energy and $F_\mathrm{em}$ is the electromagnetic form factor [289]. For definiteness we have considered a scalar field $\Sigma _e$ with the same quantum numbers as of the right-handed charged leptons (the assumptions on the flavor structure of the couplings with the DM are the same as stated at the beginning of the section).

**Fig. 18:**
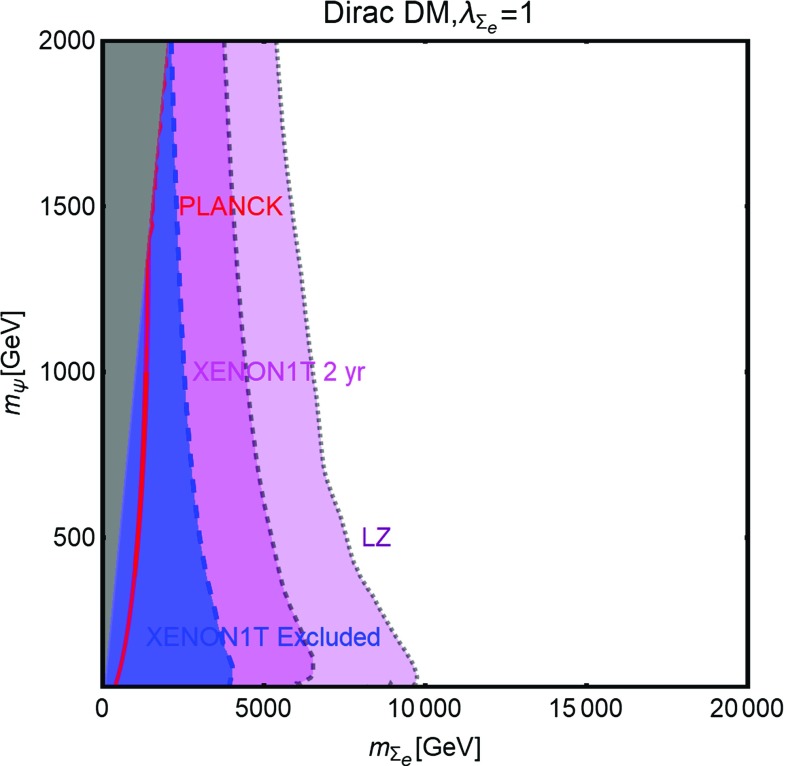
The same as the top panel of Fig. 17 but for a scalar mediator with the same quantum numbers as of the right-handed charged leptons

The impact of the corresponding current/projected constraints is shown in the usual fashion in Fig. 18. Although sensitively weaker with respect to the case of charged scalar mediator, DD constraints are still capable of ruling out the region corresponding to the correct DM relic density, for order one assignation of the coupling. As further test of this scenario one could apply the limits from searches of direct production of sleptons in SUSY models [290]. These limits, however, are weaker with respect to the ones from DD and hence, for simplicity, have not been reported in Fig. 18.

## Dark portals (partially) evading direct detection

### Pseudoscalar portal

We will investigate in this subsection the phenomenology of a pseudoscalar s-channel portal. Under the assumption of CP-invariant interactions, as performed throughout this paper, only a fermionic DM can be considered in this case. For simplicity we will describe only a Dirac fermion DM since the Majorana case features no substantial differences. We will then consider the following Lagrangian:

71 $$\begin{aligned} {\mathscr {L}}=-i \lambda _\psi ^a \overline{\psi }\gamma _5 \psi a-i \sum _f \frac{c_a}{\sqrt{2}} \frac{m_f}{v_h}\overline{f} \gamma _5 f a, \end{aligned}$$

where we have assumed, similar to the case of a scalar mediator, Yukawa-like couplings of the mediator with the SM fermions, ensuring a $SU(2)_L$ invariant construction (more complete realizations of this scenario have been considered e.g. in [165, 291–293]).

The DM relic density is determined by annihilation into the SM fermions pairs and, when kinematically accessible, into *aa* pairs. At the leading order in the velocity expansion the corresponding cross-section can be analytically approximated as follows:

72 $$\begin{aligned} \langle \sigma v \rangle {(\overline{\psi }\psi \rightarrow \overline{f}f)}\approx & {} \sum _f \frac{n_c^f c_a^2 {(\lambda _\psi ^a)}^2}{2 \pi }\frac{m_f^2}{v_h^2}\nonumber \\&\times \, \left\{ \begin{array}{ll} \frac{m_\psi ^2}{m_a^4}\quad \mathrm{for}\ m_\psi < m_a, \\ \frac{1}{16 m_\psi ^2}\quad \mathrm{for}\ m_\psi > m_a. \end{array} \right. \end{aligned}$$

This cross-section is s-wave dominated (hence capable of indirect DM signals). Given the Yukawa structure of the couplings with the SM fermions this cross-section is sizable, away from s-channel resonances, only for $m_\psi > m_t$. In such a case a numerical estimate is given by:

73 $$\begin{aligned} \langle \sigma v \rangle (\overline{\psi }\psi \rightarrow \overline{t} t)\approx \left\{ \begin{array}{l} 2.5 \times 10^{-25}\, {\text{ cm }}^3 \,{\text{ s }}^{-1} {(\lambda _\psi ^a)}^2 c_a^2 \\ \quad \times {\left( \frac{m_\psi }{300\,\text{ GeV }}\right) }^2 {\left( \frac{1\,\text{ TeV }}{m_a}\right) }^4 \quad \mathrm{for}\ m_\psi < m_a, \\ 1.9 \times 10^{-24}\, {\text{ cm }}^3 \,{\text{ s }}^{-1} {(\lambda _\psi ^a)}^2 c_a^2\\ \quad \times {\left( \frac{m_\psi }{300\,\text{ GeV }}\right) }^2 {\left( \frac{1\,\text{ TeV }}{m_a}\right) }^4\quad \mathrm{for}\ m_\psi > m_a. \end{array} \right. \end{aligned}$$

The annihilation cross-section into *aa* pairs is, instead, p-wave suppressed:

74 $$\begin{aligned}&\langle \sigma v \rangle {(\overline{\psi }\psi \rightarrow aa)} \approx \frac{{(\lambda _\psi ^a)}^2}{192 \pi m_\psi ^2}v^2, \nonumber \\&\quad \approx 2.3 \times 10^{-26}\, {\text{ cm }}^3 \,{\text{ s }}^{-1} times {(\lambda _\psi ^a)}^2 {\left( \frac{500\,\text{ GeV }}{m_\psi }\right) }^2. \end{aligned}$$

The most peculiar feature of the pseudoscalar portal scenario is, nevertheless, the weakening of the interactions possibly responsible of DD.^24^ Indeed, tree-level interactions between the DM and the SM quarks (and gluons), mediated by a pseudoscalar, are momentum suppressed. This can be described by the following differential interaction cross-section for a target nucleus of mass $m_T$ [295]:

75 $$\begin{aligned} \frac{d \sigma _T}{dE_R}= & {} \frac{{(\lambda _\psi ^a)}^2 c_a^2}{128 \pi }\frac{q^4}{m_a^4} \frac{m_T^2}{m_\chi m_N}\frac{1}{v_E^2}\sum _{N,N'=p,n} g_N g_{N'} F_{\Sigma ^{''}}^{NN'}(q^2), \nonumber \\ g_N= & {} \sum _{q=u,d,s} \frac{m_N}{v}\left[ 1-\frac{\overline{m}}{m_q}\right] \varDelta _q^{N},\nonumber \\&\quad \mathrm{with}\ \overline{m}={\left( 1/m_u+1/m_d+1/m_s\right) }^{-1}, \end{aligned}$$

**Fig. 19:**
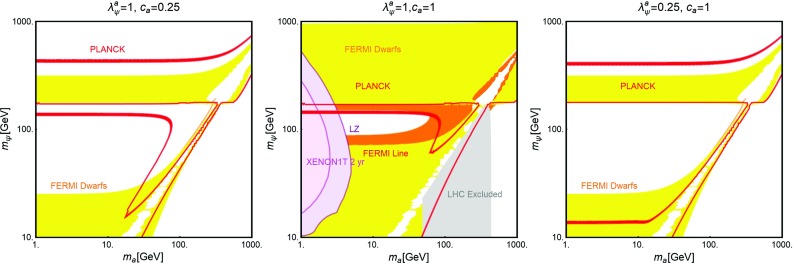
Summary of constraints from the phenomenology of a fermionic DM in the case of s-channel pseudoscalar mediator. These are reported in the bi-dimensional $(m_a,\,m_\psi )$ plane for the three assignations of $(\lambda _\psi ^a,\,c_a)$ parameters, namely, $(1,\,0.25)$ (left), $(1,\,1)$ (middle) and $(0.25,\,1)$ (right). The red lines are the iso-contours of the correct DM relic density. In the yellow regions the DM annihilation cross-section into SM fermions, computed at the present time, exceeds the limit given by FERMI from searches of signals in dSphs [105]. In the orange regions the loop-induced annihilation cross-section into photon lines exceeds limits set by searches of gamma-ray lines [102]. For $(\lambda _\psi ^a,\,c_a)=(1,\,1)$ an exclusion region (grey) from the LHC searches of monojet events [133], as well as projected exclusion regions from XENON1T (magenta) and LZ (purple) are also reported

where $v_E$ represents the DM speed in the Earth frame, $\varDelta ^N_q$ denotes quark spin content of the nucleon, $E_R$ is the nuclear recoil energy with recoil velocity *q*, and $F^{NN'}_{\Sigma ^{''}}$ are (squared) form factors whose (approximate) analytical expressions are given in Ref. [296]. The cross-section of Eq. (75) corresponds neither to SI nor to SD (although the latter is a good approximation) interactions; for this reason we have expressed it directly in terms of a differential cross-section on a nucleus. As can be easily argued that the $q^4$ dependence of Eq. (75) implies a very suppressed scattering rate so that a potentially detectable signal is produced only for $m_a \sim \mathscr {O}(10\,\text{ MeV })$ [295]. These low values are, however, subject to bounds from low energy observables and rare flavor processes (see e.g., Ref. [297] for an extensive analysis). For these reason we will consider here (and also in this section) values of $m_a$ above 1 GeV.^25^ We also remark that Xenon based detectors, like the ones considered in our study, would be in any case not suited for probing the interaction cross-section of Eq. (75) since it originates from a coupling of the DM mostly with unpaired protons, not present in Xenon, which instead has isotopes with an odd number of neutrons.

In the considered setup the most relevant interactions originate at the loop level, from a box diagram in which a pair of pseudoscalars, one DM and one quark state are exchanged [291], and are of SI-type. The corresponding cross-section is given by:

76 $$\begin{aligned}&\sigma _{\psi p}^\mathrm{SI}=\frac{\mu _{\psi p}^2}{\pi }{\left| \sum _q \alpha _q f^p_q\right| }^2, \nonumber \\&\quad \mathrm{with}\ \alpha _q=\frac{m_q^2 {(\lambda _\psi ^a)}^2 c_a^2}{128 \pi ^2 m_a^2 \left( m_\psi ^2-m_q^2\right) } \frac{m_\psi m_q}{v^2}\nonumber \\&\quad \times \left[ F\left( \frac{m_\psi ^2}{m_a^2}\right) -F\left( \frac{m_q^2}{m_a^2}\right) \right] ,\nonumber \\&F(x)=\frac{2}{3x}\left[ 4+f_{+}(x)+f_{-}(x)\right] ,\nonumber \\&f_{\pm }(x)=\frac{1}{x}\left( 1 \pm \frac{3}{\sqrt{1-4x}}\right) {\left( \frac{1+\pm \sqrt{1-4x}}{2}\right) }^3 \nonumber \\&\quad \times \log \left( \frac{1 \pm \sqrt{1-4x}}{2}\right) , \end{aligned}$$

where the sum in the first row involves all quarks. The coefficients $f_q^p$ are the same as the ones defined in the case of real scalar portals (we remind $f^p_{c,b,t}=\frac{2}{27}f_{TG}^p$, as mentioned in the context of Eq. (35)).

As evidenced in Fig. 19, this cross-section is very suppressed so that no constraints come from present experiments. On the contrary, for $\mathscr {O}(1)$ values of both the $\lambda _\psi ^a$ and $c_a$ couplings, next generation detectors can partially probe the parameter space corresponding to the thermal DM.

More stringent constraints come from ID of the DM. Contrary to the scalar mediator case the DM annihilation cross-section into SM fermions is now s-wave dominated. These processes lead to potential signals in the gamma-ray continuum which can be probed by the FERMI satellite [105]. In addition, the DM can also pair annihilate into two photons, through an effective coupling between the pseudoscalar mediator and photons, generated by triangle loops of the SM fermions [105]. This process, responsible for the generation of gamma-ray lines, is strongly constrained by the negative results in present searches [102].

The summary of our analysis is presented, in the usual fashion, in Fig. 19. The figure features three panels corresponding to the assignations $(\lambda _\psi ^a,\,c_a)=(1,\,0.25)$, $(1,\,1)$, $(0.25,\,1)$.

As already anticipated that the most stringent constraints comes from indirect DM searches in dSphs.^26^ Indeed, the absence of signals excludes thermal DM for mass below approximately 50 GeV. The most disfavored scenario turns to be the one corresponding to the assignation $\lambda _\psi ^a=c_a=1$ (middle plot of Fig. 19). Indeed a complementary constraint comes from searches of gamma-ray lines so that the excluded ranges of the DM masses reaches the order of 100 GeV. This specific assignation of the couplings has also been investigated at the LHC through searches of events with monojet and missing transverse momentum. Due to the absence of any signal, the regions of the parameter space corresponding to $100 \lesssim m_a \lesssim 500\,\text{ GeV }$ as well as $m_a > 2 m_\psi$ are currently excluded.

### Scalar + light pseudoscalar portal

In this subsection we will consider the case in which the pseudoscalar mediator is actually a component of a complex scalar field $\varPhi \rightarrow (S+ia)/\sqrt{2}$, described by the following Lagrangian:

77 $$\begin{aligned} {\mathscr {L}}_\varPhi= & {} \partial _\mu \varPhi \partial ^\mu \varPhi ^{*}+\mu _\varPhi ^2 |\varPhi |^2-\lambda |\varPhi |^4 \nonumber \\&+\,\frac{\epsilon _\varPhi ^2}{2}\left( \varPhi ^2 + \text{ h.c. }\right) . \end{aligned}$$

After the EWSB, the scalar component of $\varPhi$ gets non-zero VEV $(v_\varPhi )$, generating a mass term of its scalar component, $m_S=\sqrt{2\lambda }v_\varPhi$ while leaving the pseudoscalar component massless. A mass, $m_a=\sqrt{2} \epsilon _\varPhi$, for this second field is originated by an explicit mass term $\epsilon _\varPhi ^2\varPhi ^2/2$ in Eq. (77). In this kind of setup it is rather natural to identify the pseudoscalar component with a pseudo-Goldstone boson associated to a *U*(1) global symmetry carried by the complex scalar $\varPhi$ and then assumes that $m_a \ll m_S$ [298–300]. We further assume that after the EWSB it is possible to write interaction terms of the fields *S* and *a* both with the SM fermions and a fermionic DM candidate as follows:

78 $$\begin{aligned} -{\mathscr {L}}= & {} \frac{m_S^2}{2}S^2+\frac{m_a^2}{2} a^2+\sqrt{\frac{\lambda }{2}}m_S Sa^2 \nonumber \\&+\,\sqrt{\frac{\lambda }{2}}m_S S^3+\frac{\lambda }{4}{\left( S^2+a^2\right) }^2 \nonumber \\&+\,m_\psi \overline{\psi }\psi +g_\psi \left( S \overline{\psi }\psi +i a \overline{\psi }\gamma ^5 \psi \right) \nonumber \\&+\,\sum _f c_S \frac{m_f}{v_h}\left( S \overline{f} f+i a \overline{f} \gamma ^5 f\right) . \end{aligned}$$

Here we have again assumed Yukawa-like interactions among $S,\,a$ and the SM fermions where the concerned couplings, including a normalization factor of 1/$\sqrt{2}$, are parametrized as $c_S$. The other free parameters are the DM mass and coupling, $m_\psi$ and $g_\psi$.^27^

The main feature of this double s-channel portal scenario is the lower amount of correlation between the direct DM detection and the relic density, due to the presence of a light mediator state (see also Ref. [301] for a similar idea).

Indeed, DD of the DM relies only on the coupling of the DM with the scalar component of the $\varPhi$ field. The DM relic density, instead, is determined by the different annihilation channels, including $\overline{f} f$, *aa*, *Sa* and *SS* involving interactions of both *S* and *a* fields. The expressions for the annihilation cross-sections into fermion and *S* pairs can be derived straightforwardly from the cases of the scalar and pseudoscalar portals and thus, won’t be re-discussed in detail. The annihilation cross-section into *a* pairs, despite the presence of an additional contribution from s-channel exchange of *S* is only moderately altered with respect to the case of the pseudoscalar portal (see Sect. 10.1) and we just then refer to its detailed expression presented in the Appendix. The most prominent feature, relevant for the DM relic density, is the presence of *Sa* as final state for annihilation processes, when allowed kinematically. To this corresponds, in fact, an efficient s-wave annihilation cross-section which can be analytically approximated as:

79 $$\begin{aligned} \langle \sigma v \rangle {(\overline{\psi }\psi \rightarrow Sa)}=\left\{ \begin{array}{l} \frac{g_\psi ^2 \lambda m_S^4}{512 \pi m_\psi ^6}\approx 1.6 \times 10^{-25}\, {\text{ cm }}^3\, {\text{ s }}^{-1}\\ \quad \times \, g_\psi ^2 \lambda {\left( \frac{m_S}{1\,\mathrm{TeV}}\right) }^4 {\left( \frac{600\,\mathrm{GeV}}{m_\psi }\right) }^6 \\ \quad \mathrm{for}\ m_\psi < m_S, \\ \frac{g_\psi ^4}{16 \pi m_\psi ^2} \approx 2.3 \times 10^{-25}\, {\text{ cm }}^3\, {\text{ s }}^{-1} \\ \quad \times \, g_\psi ^4 {\left( \frac{1\,\mathrm{TeV}}{m_\psi }\right) }^2 \quad \mathrm{for}\ m_\psi > m_S. \end{array} \right. \end{aligned}$$

**Fig. 20:**
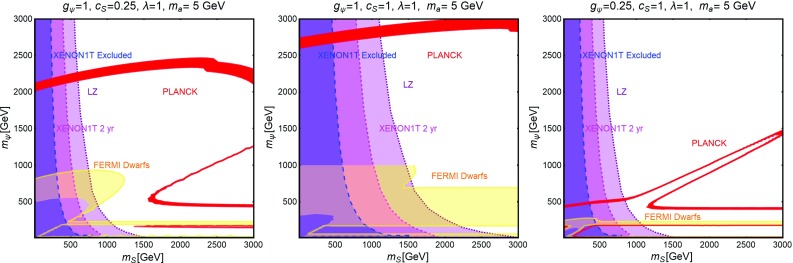
Summary of constraints for the scalar $+$ light pseudoscalar portal with a fermionic DM in the bi-dimensional plane $(m_S,\,m_\psi )$ with $m_a,\,\lambda$ set to 5 GeV, 1, respectively. The three plots refer to the three assignations of $(g_\psi ,\,c_S)$ couplings as $(1,\,0.25)$ (left), $(1,\,1)$ (middle) and $(0.25,\,1)$ (right). In each plot the red lines are the contours of the correct DM relic density. The blue, magenta and purple regions represent the current exclusion by XENON1T and projected exclusions from XENON1T and LZ, respectively. In the yellow regions the DM annihilation cross-section at the present time exceeds the limits determined by FERMI from searches of DM annihilations in dSphs

The comparison between the DM relic density and the experimental (present and future) constraints, is performed, in the usual fashion, in Fig. 20. Here we have chosen the DM mass $m_\psi$ and the scalar mass $m_S$ as the two free parameters while $m_a$ has been set to 5 GeV, in order to avoid dangerous constraints from low energy physics. We have then considered three assignations for $(g_\psi , c_S)$ couplings, i.e., (1, 0.25), (1, 1) and (0.25, 1) while the value of coupling $\lambda$ of the scalar potential has been set to 1.

As evident that the presence of annihilation channels, like *aa* and *Sa*, involving non-SM light states allows to achieve the correct relic density, compatibly with constraints from DM DD, for relatively low values of the DM mass without necessarily relying on s-channel resonances. The presence of s-wave unsuppressed annihilation channels like $\overline{f}f$ (contribution from s-channel exchange of the pseudoscalar) and *Sa*^28^ makes mandatory to consider, besides DD, also limits from ID. However, for the chosen parameter assignation, these last constraints have no impact in the region corresponding to the correct DM relic density.

## Portals to secluded sectors

In this section we will consider cases in which the mediator of the DM interactions has no direct coupling with the SM fermions. Dark portals can nevertheless be realized, at the renormalizable level. Indeed the SM features two Lorentz and gauge invariant bilinears, i.e., $H^\dagger H$ and $B^{\mu \nu },$ which together with the similar appropriate structures can connect the SM and BSM sectors. The first one, $H^\dagger H$, can be coupled to another scalar bilinear. In case this second scalar field also has non-zero VEV, a mass mixing with the SM-Higgs is generated so that the portal interactions with the SM fermions, as well as the gauge and the SM-Higgs bosons itself are produced. The field strength $B^{\mu \nu }$ can be coupled with the field strength of another *U*(1) gauge bosons. Such kinetic mixing term after the EWSB, is the origin of a mixing between the *Z* and the new $Z'$ boson.

In both these two scenarios, the DM interacts with the SM fields (both fermions and bosons) through a double s-channel mediator. The relevant phenomenological processes are substantially the same as already investigated in the cases of the SM and BSM s-channel dark portals. Contrary to these scenarios, we cannot consider $\mathscr {O}(1)$ couplings between the SM states and the BSM mediators, as well as between the DM and the SM ones, since the mixing between the SM-Higgs and an additional scalar or the Z and the Z’ are required to be small by several experimental and theoretical constraints.

### SM-Higgs + spin-0 portal

In this subsection we will revisit the phenomenology of a real spin-0 mediator $\varPhi$ considering the more realistic case in which it also features interaction with the SM-Higgs doublet *H*. We will thus assume the following scalar potential:

80 $$\begin{aligned} V=V_{H,\mathrm SM}+V(H,\varPhi ), \end{aligned}$$

where $V_{H,\mathrm SM}$ is the SM scalar potential (${\lambda _h}{(H^\dagger H)}^2+\mu ^2_h H^\dagger H$) while:

81 $$\begin{aligned} V(H,\varPhi )={\lambda _{hS}} H^\dagger H {\varPhi }^2+{\lambda _\varPhi }{\varPhi }^4+\mu ^2_\varPhi {\varPhi }^2, \end{aligned}$$

here $\mu _h,\,\mu _\varPhi$ are parameters with the dimension of mass and $\mu ^2_h,\,\mu ^2_\varPhi <0$ for spontaneous symmetry breaking.

One should note that in $V_{H,\mathrm SM}+V(H,\varPhi )$, the condition for getting a positive definite mass spectrum requires $4\lambda _h \lambda _\varPhi > \lambda ^2_{hS}$ while $\lambda _h,\,\lambda _\varPhi >0$ are necessary to get $V_{H,\mathrm SM}+V(H,\varPhi )$ bounded from below. Combining these two conditions we see that $\lambda _{hS}$ can take both the positive and negative values.

We denote non-zero VEV of the scalar field $\varPhi$ as $v_\varPhi$, so that it can be expanded as $\varPhi =\left( v_\varPhi +\phi \right) /\sqrt{2}$. The coupling of $\varPhi$ with the SM-Higgs doublet *H* induces mass mixing such that, after the EWSB (assuming unitary gauge), one can define the following two mass eigenstates [304]:

82 $$\begin{aligned} \left( \begin{array}{c} h \\ S \end{array} \right) = \left( \begin{array}{cc} \cos \theta &{} \sin \theta \\ -\sin \theta &{} \cos \theta \end{array} \right) \left( \begin{array}{c} \mathfrak {R}{H(0)} \\ \phi \end{array} \right) , \end{aligned}$$

where $\mathfrak {R}{H(0)}$ represents the electrically neutral scalar part of the SM Higgs doublet *H* and the mixing angle $\theta$ is defined by:

83 $$\begin{aligned} \tan 2\theta =\frac{\lambda _{hS}v_h v_\varPhi }{\lambda _h v_h^2-\lambda _\varPhi v_\varPhi ^2}. \end{aligned}$$

The phenomenology of the mediator sector, thus, can be expressed as functions of the five free parameters $\lambda _h$, $\lambda _\varPhi$, $v_h$, $v_\varPhi$, $\lambda _{hS}$ or equivalently in terms of $m^2_h$, $m^2_S,v_h$, $\sin \theta ,\,\lambda _{hS}$ using the following relations [304–306]:

84 $$\begin{aligned}&\lambda _h = \frac{m^2_h}{2 v^2_h} + \frac{(m^2_S-m^2_h)\sin ^2\theta }{2v^2_h}, \nonumber \\&\lambda _\varPhi = \frac{2\lambda ^2_{hS} v^2_h}{\sin ^22\theta (m^2_S-m^2_h)} \left( \frac{m^2_S}{(m^2_S-m^2_h)}-\sin ^2\theta \right) ,\nonumber \\&\quad {\mathrm{and}}\quad v_\varPhi = \frac{(m^2_h-m^2_S)\sin 2\theta }{2\lambda _{hS}v_h}. \end{aligned}$$

Note that in reality we have only three free parameters, i.e., $\lambda _{hS},\,\sin \theta$ and $m_S$ as $m_h,\,v_h$ have known measured values [137].

The mixing between $\mathfrak {R}{H(0)}$ and $\phi$ (see Eq. (82)) indicates that both the mass eigenstates $(h,\,S)$ will couple to the SM states as well as to the DM and thus, it represents a two-portal scenario.

The couplings of the two s-channel mediators with the $W^\pm ,\,Z$-bosons and the SM fermions are described by:

85 $$\begin{aligned} {\mathscr {L}}^{hS}_\mathrm{SM}= & {} \frac{h \cos \theta - S \sin \theta }{v_h}\nonumber \\&\times \left[ 2 m_W^2 W^{+}_\mu W^{\mu -} + m_Z^2 Z^\mu Z_\mu -\sum _f m_f \overline{f} f \right] ,\nonumber \\ \end{aligned}$$

while their cubic self-couplings are given by:

86 $$\begin{aligned} {\mathscr {L}}_{hS}= & {} -\frac{\kappa _{hhh}v_h}{2}~h^3-\frac{\kappa _{hhS}v_h}{2}\sin \theta ~ h^2 S\nonumber \\&- \frac{\kappa _{hSS}v_h}{2}\cos \theta ~h S^2-\frac{\kappa _{SSS}v_h}{2}~ S^3, \end{aligned}$$

with:

87 $$\begin{aligned} \kappa _{hhh}= & {} \frac{m^2_h}{v^2_h\cos \theta } \left( \cos ^4\theta + \sin ^2\theta \frac{\lambda _{hS}v^2_h}{(m^2_h-m^2_S)}\right) ,\nonumber \\ \kappa _{SSS}= & {} \frac{m^2_S}{v^2_h\sin \theta } \left( \sin ^4\theta + \cos ^2\theta \frac{\lambda _{hS}v^2_h}{(m^2_S-m^2_h)}\right) ,\nonumber \\ \kappa _{hhS}= & {} \frac{2 m_h^2+m_S^2}{v_h^2}\left( \cos ^2 \theta +\frac{\lambda _{hS}v_h^2}{(m_S^2-m_h^2)}\right) ,\nonumber \\ \kappa _{hSS}= & {} \frac{2 m_S^2+m_h^2}{v_h^2}\left( \sin ^2 \theta +\frac{\lambda _{hS} v_h^2}{(m_h^2-m_S^2)}\right) . \end{aligned}$$

The parameters $\lambda _{hS}$ and $\sin \theta$ are subject to several experimental and theoretical constraints (see e.g., Refs. [304, 307–309] for an extensive discussion). For example, a non-zero value of $\theta$ modifies the couplings of the SM-Higgs with SM particles and thus, is constrained by the measurement of the Higgs signal strengths. The coupling $\lambda _{hS}$ is instead constrained by the stability of the scalar potential. Most of these constraints become increasingly stringent as $m_S$ decreases; for this reason we will focus our analysis on the case $m_S > m_h$.

Similar to the other spin-0 mediator scenarios, this extended Higgs sector will be coupled to a scalar DM $\chi$, a fermionic (we will restrict to the Dirac case) DM $\psi$ and a spin-1 DM $V_\mu$. We will consider the following Lagrangians for the corresponding interactions.

**Fig. 21:**
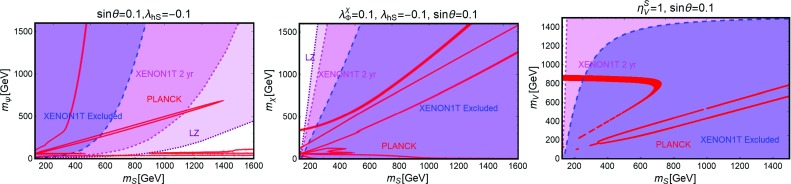
Summary of constraints for the SM-Higgs $+$ spin-0 portal for a scalar (left), Dirac fermion (middle) and a vectorial (right) DM in the relevant bi-dimensional planes, $(m_S,\,m_\chi ),\,(m_S,\,m_\psi )$ and $(m_S,\,m_V)$, respectively. The red contours represent the correct DM relic density while the blue, magenta and purple regions represent the current exclusion by XENON1T and the projected sensitivities of the XENON1T and LZ, respectively. The details concerning the assignations of the model parameters are discussed in the main text

In the case of a scalar DM^29^ we consider the following simplified (i.e., assuming vanishing coupling for ${|\chi |}^4$ term) interaction:

88 $$\begin{aligned} - {\mathscr {L}}_{\chi }=\lambda _H^\chi {|\chi |}^2 H^\dagger H +\lambda _\varPhi ^\chi {|\chi |}^2 {\varPhi }^2 +\mu ^2_\chi {|\chi |}^2, \end{aligned}$$

which, after the EWSB, leads to the following effective Lagrangian:

89 $$\begin{aligned} - {\mathscr {L}}_\chi= & {} g_{\chi \chi h}|\chi |^2 h+g_{\chi \chi S}|\chi |^2 S+g_{\chi \chi hh}|\chi |^2 h^2 \nonumber \\&+\,g_{\chi \chi hS}|\chi |^2 h S+g_{\chi \chi SS}|\chi |^2 S^2 +m^2_\chi {|\chi |}^2, \end{aligned}$$

where:

90 $$\begin{aligned}&g_{\chi \chi h}=\left( \lambda _H^\chi v_h \cos \theta + \lambda _\varPhi ^\chi \sin ^2\theta \cos \theta \frac{(m^2_h-m^2_S)}{\lambda _{hS} v_h}\right) ,\nonumber \\&g_{\chi \chi S}=\left( -\lambda _H^\chi v_h \sin \theta + \lambda _\varPhi ^\chi \cos ^2\theta \sin \theta \frac{(m^2_h-m^2_S)}{\lambda _{hS}v_h} \right) ,\nonumber \\&g_{\chi \chi h h}=\frac{1}{2}\left( \lambda _H^\chi \cos ^2\theta + \lambda _\varPhi ^\chi \sin ^2\theta \right) ,\nonumber \\&g_{\chi \chi h S}=\left( -\lambda _H^\chi \sin \theta \cos \theta + \lambda _\varPhi ^\chi \sin \theta \cos \theta \right) ,\nonumber \\&g_{\chi \chi S S}=\frac{1}{2}\left( \lambda _H^\chi \sin ^2\theta + \lambda _\varPhi ^\chi \cos ^2\theta \right) ,\nonumber \\&\quad \mathrm{and}\quad m^2_\chi =\mu ^2_\chi + \frac{1}{2} \lambda ^\chi _H v^2_h + \frac{1}{2} \lambda ^\chi _\varPhi v^2_\varPhi , \end{aligned}$$

using Eq. (84). From Eq. (90) it can be seen that without the loss of generality one of the couplings $\lambda _H^\chi$ and $\lambda _\varPhi ^\chi$ can be set to zero. We will thus, pursue this minimal choice setting $\lambda _H^\chi =0$. With this choice one can consider the following five parameters $\lambda ^\chi _\varPhi ,\,\lambda _{hS},\,\sin \theta ,\,m_S$ and $m_\chi$ as the free inputs.

In the case of a fermionic DM it is natural, in this setup, to assume a Yukawa-type coupling with the field $\varPhi$ of the form:

91 $$\begin{aligned} {\mathscr {L}}_\psi =-y_\psi \overline{\psi }\psi \varPhi ; \end{aligned}$$

here the DM mass is dynamically generated by the VEV of $\varPhi$ so that the DM mass and its coupling with $\varPhi$ are not independent but are related as $y_\psi \propto {m_\psi }/{v_\varPhi }$. This choice allows to reduce the number of free parameters compared to the scalar DM scenario and hence, $\lambda _{hS},\,m_S,\,\sin \theta$ and $m_\psi$ remain the four free input parameters. The effective couplings of the DM with the *S* and *h* fields can be straightforwardly derived by using Eqs. (82) and (84).

A dynamical generation of the DM mass has also been considered in the case of a vectorial DM. We indeed identify the DM as the stable gauge boson of a *U*(1) dark gauge group,^30^ spontaneously broken by the VEV of a complex scalar field $\varPhi$. The interactions between the latter and the DM are then embedded in the covariant derivative $(D_\mu \varPhi )^{*}$ $D^\mu \varPhi$ with $D_\mu =\partial _\mu -i \frac{\eta _V^S}{2}V_\mu$.^31^ After spontaneous symmetry breaking the DM Lagrangian reads:

92 $$\begin{aligned} {\mathscr {L}}_{V}=\frac{1}{2}{\eta _V^S}m_V V^\mu V_\mu \phi + \frac{(\eta _V^S)^2}{8} \phi ^2 V^\mu V_\mu + \frac{1}{2}m^2_V V^\mu V_\mu , \end{aligned}$$

where we have used the expression of $\varPhi$ in the unitary gauge as $\varPhi =\frac{1}{\sqrt{2}} \left( v_\varPhi +\phi \right)$ and $m_V=\frac{1}{2} \eta ^S_V v_\varPhi$. The coupling $\eta ^S_V$ here thus represents a gauge coupling.

Note that for the vectorial DM scenario we have the following parameters, namely $m^2_h$, $m^2_S$, $v_h$, $\sin \theta$, $\lambda _{hS}$ from the mediator sector (starting with $\lambda _h$, $\lambda _\varPhi$, $\lambda _{hS}$, $v_\varPhi$, $v_h$, see Eq. (84)) and $\eta ^S_V,\,m_V$ from the DM interaction Lagrangian (see Eq. (92)). However, one can now trade $v_\varPhi = 2 m_V/ \eta ^S_V$ with the last relation of Eq. (84) such that

93 $$\begin{aligned} \lambda _{hS}=\frac{(m^2_h-m^2_S)\sin 2\theta }{2v_h}\times \frac{\eta ^S_V}{2m_V}. \end{aligned}$$

It is now apparent that one can consider the following four parameters, i.e., $m_S$, $\sin \theta$, $\eta ^S_V$ and $m_V$ as the free inputs for a vectorial DM in the SM-Higgs + spin-0 portal scenario. Hence, one should replace the *new derived* parameter $\lambda _{hS}$ judiciously in Eqs. (83), (84), (87) and (90) for numerical analysis.

The processes responsible for the DM relic density and DD have been already discussed in detail for the cases of the SM-Higgs and scalar portal individually; we thus just illustrate the results of our analysis, as reported in Fig. 21. We just remind here that all the considered types of DM candidates feature SI interactions with nuclei.

The results are reported in the usual bi-dimensional planes, i.e., $(m_S,\,m_\chi )$, $(m_S,\,m_\psi )$ and $(m_S,\,m_V)$, respectively. The angle $\theta$ has been conservatively set to $\sin \theta =0.1$ in order to comply with the constraints from the SM-Higgs signal strengths. Similarly, the coupling $\lambda _{hS}$ has been set to a value of $-\,0.1$ in order to satisfy various constraints on the SM-Higgs sector (see e.g., Ref. [304] for a detailed discussion). The couplings $\lambda ^\chi _\varPhi$, $\eta ^S_V$ have been set to 0.1 and 1, respectively while for the fermionic DM the associated Yukawa coupling $y_\psi$ is not a free parameter in our construction.

It is evident that the outcome of our analysis presents some sensitive differences with respect to the case of the spin-0 mediator discussed in the previous sections. In the case of a fermionic and a vectorial DM the limits from DD are rather effective, due to the presence of an additional light mediator. In the case of a vectorial DM the only viable region, for masses of the DM and the mediator below the TeV scale, corresponds to the case $m_V > m_S$, thanks to the enhancement of the DM annihilation cross-section due to $VV \rightarrow SS$ process. In the case of a fermionic DM the region surviving the current constraints corresponds to the “pole” $m_\psi \sim m_S/2$. In both cases, the next generation of DD detectors will probe the WIMP paradigm for masses of the DM and the mediator up to a few TeV. More particular is the case of a scalar DM where the shape of the DD contours is rather different compared to the other spin-0 mediators. This is due to the different choice of the energy scale, the VEV $v_\varPhi$ rather than the mass of the mediator, which implies a larger cross-section at higher values of $m_S$. On the contrary, as can be noticed from Eq. (90), for $\lambda _H^\chi =0$ the DM couplings become smaller as $m_h$ and $m_S$ get close in their values.^32^ For the chosen parameter assignations, a large region of the viable thermal DM is present in the regime $m_\chi > m_S$ for a rather light $m_S$. A sizable part of this region will be excluded in the absence of signals at XENON1T and LZ.

### Kinetic mixing

We will reconsider in this subsection the scenario in which the DM is coupled to the gauge boson of a new *U*(1) group. In this case, however, SM fermions won’t be charged under the new gauge group so that no direct couplings with the new gauge boson are induced. The “dark” and visible sectors can nevertheless be connected by a kinetic mixing operator $B_{\mu \nu } X^{\mu \nu }$ which can already exist at the tree level, being both Lorentz and gauge invariant, or generated radiatively (for example, if the new gauge sector features new fermions having non-trivial quantum numbers also under the SM gauge group [315]).

We will then consider the following Lagrangian with $\delta$ as the kinetic mixing parameter:

94 $$\begin{aligned} {\mathscr {L}}= & {} -\frac{1}{4}B^{\mu \nu }B_{\mu \nu }-\frac{1}{4}X^{\mu \nu }X_{\mu \nu }-\frac{1}{2} \sin \delta B^{\mu \nu }X_{\mu \nu } \nonumber \\&+\,\frac{1}{2}m^2_{X} X^\mu X_\mu +\mathscr {L'}_\mathrm{SM}+{\mathscr {L}}_\mathrm{DM}, \end{aligned}$$

where $B^{\mu \nu }$ and $X^{\mu \nu }$ are the fields strength of the $U(1)_Y$ and the new *U*(1) group (associated with $X_\mu$), respectively. $\mathscr {L'}_\mathrm{SM}$ is the SM Lagrangian besides the already written kinetic term of $B_{\mu }$, while ${\mathscr {L}}_\mathrm{DM}$ is the DM Lagrangian including the kinetic and mass terms as well as a coupling with the $X_\mu$ boson. These terms will depend on the spin assignation of the DM. We will consider the following cases:

95 $$\begin{aligned} {\mathscr {L}}_\mathrm{DM}= \left\{ \begin{array}{cc} {\mathscr {L}}_\mathrm{DM}=(D^\mu \chi )^*D_\mu \chi -m_\chi ^2 \chi ^{*}\chi \\ \text{(complex } \text{ scalar) },\\ \\ {\mathscr {L}}_\mathrm{DM}=\overline{\psi }\gamma ^\mu D_\mu \psi -m_\psi \overline{\psi }\psi \\ \text{(Dirac } \text{ fermion) },\\ \\ {\mathscr {L}}_\mathrm{DM}=\eta _V^X [[VVX]]+m_V^2 V_\mu ^{\dagger }V^{\mu } \\ \text{(non-Abelian } \text{ vector) }.\\ \end{array} \right. \end{aligned}$$

As already discussed, a natural option to couple a scalar or a fermionic DM to a new gauge boson is to assume it charged under the new symmetry group so that its interactions originate from the covariant derivative $D_\mu =\partial _\mu -i g_X X_\mu$.^33^ Slightly more complicated is the case of a vectorial DM. Here we have assumed an analogous coupling, as the one of the SM, between the Z and two W bosons.^34^ The coupling $\eta _V^X$ encodes the gauge coupling $g_X$ and other eventual extra factors arising from a specific model construction. $m_{\chi ,\, \psi ,\, V}$ denote the respective DM masses of different spin assignments.

The kinetic term of Eq. (94) should be diagonalized and canonically normalized. After the EWSB, it is possible to define three mass eigenstates, i.e., $A_\mu$, $Z_\mu$ and $Z'_\mu$, for the electrically neutral gauge bosons through the following two transformations [246–248, 316]:

96 $$\begin{aligned} \left( \begin{array}{c} B_\mu \\ W^3_\mu \\ X_\mu \end{array} \right) = \left( \begin{array}{ccc} 1 &{} 0 &{} -t_\delta \\ 0 &{} 1 &{} 0 \\ 0 &{} 0 &{} 1/c_\delta \end{array} \right) \left( \begin{array}{ccc} c_{\hat{W}} &{} -s_{\hat{W}} c_\xi &{} s_{\hat{W}} s_\xi \\ s_{\hat{W}} &{} c_{\hat{W}} c_\xi &{} -c_{\hat{W}} s_\xi \\ 0 &{} s_\xi &{} c_\xi \end{array} \right) \left( \begin{array}{c} A_\mu \\ Z_\mu \\ Z'_\mu \end{array} \right) , \end{aligned}$$

where $t_\delta ,\,c_\delta = \tan \delta ,\, \cos \delta$, $c_{\hat{W}},\,s_{\hat{W}}=\cos \theta _{\hat{W}},\,\sin \theta _{\hat{W}}$, $c_{\xi },\,s_{\xi }=\cos \xi ,\,\sin \xi$ and the angle $\xi$ is defined by:

97 $$\begin{aligned} \tan 2\xi =\frac{-2 m_{\hat{Z}}^2 s_{\hat{W}} \cos \delta \sin \delta }{m_{X}^2-m_{\hat{Z}}^2 \cos \delta ^2 +m_{\hat{Z}}^2 s_{\hat{W}}^2 \sin \delta ^2}. \end{aligned}$$

Note that the transformation given in Eq. (96) leads to physical solutions only if one of these two conditions is met [247]:

98 $$\begin{aligned} r_X\ge & {} 1+2 s_{\hat{W}} \tan ^2 \delta +2 \sqrt{s_{\hat{W}}^2 \tan ^2 \delta \left( 1+s_{\hat{W}}^2 \tan ^2 \delta \right) }\quad \mathrm{or} \nonumber \\ r_X\le & {} 1+2 s_{\hat{W}} \tan ^2 \delta -2 \sqrt{s_{\hat{W}}^2 \tan ^2 \delta \left( 1+s_{\hat{W}}^2 \tan ^2 \delta \right) }, \nonumber \\&\mathrm{where}\ r_X=\frac{m_X^2}{m_{\hat{Z}}^2}. \end{aligned}$$

In the expressions above $s_{\hat{W}},\,m_{\hat{Z}}$ do not represent the experimental measures of Weinberg angle and the *Z*-boson mass but are related to the latter, i.e., $s_W\equiv \sin \theta _W,\,m_Z$, as function of $\delta$. Indeed, since the photon coupling does not change once passing to the “physical/mass” basis, one can write:

99 $$\begin{aligned} c_W^2 s_W^2=\frac{c_{\hat{W}}^2 s_{\hat{W}}^2}{1+s_{\hat{W}}\tan \xi \tan \delta }, \quad \mathrm{with}\ c_W\equiv \cos \theta _W. \end{aligned}$$

In an analogous fashion, the invariance of the *W*-boson mass under the transformations of Eq. (96) allows to relate the kinetic mixing parameter to the $\rho$ parameter as:

100 $$\begin{aligned} \rho =\frac{c_{\hat{W}}^2}{\left( 1+s_{\hat{W}}\tan \delta \tan \xi \right) c_W^2}, \end{aligned}$$

which can be reformulated as:

101 $$\begin{aligned} \omega =s_W \tan \delta \tan \xi \simeq -(1-t_W^2) \varDelta , \end{aligned}$$

where $\varDelta =\rho -1~~\mathrm{and~~} t^2_W=\tan ^2\theta _W$. Hence, $\rho -1=4^{+8}_{-4} \times 10^{-4}$ measurement [209] can be used to constrain the parameter $\delta$.

The kinetic mixing parameter is further constrained by EW Precision Tests (EWPTs) [317, 318] which reads as:

102 $$\begin{aligned} \tan \delta \le \frac{m_{Z'}}{2.5\,\text{ TeV }}. \end{aligned}$$

The spectrum of the neutral gauge bosons features one massless eigenstate, coinciding with the SM photon, and two massive states. Their masses, in the experimentally favoured limit, i.e., $\sin \delta ,\,\sin \xi \sim \delta ,\,\xi \ll 1$, are given as:

103 $$\begin{aligned} m_Z^2\simeq & {} m_{\hat{Z}}^2+\left( m_{\hat{Z}}^2-m_X^2\right) \xi ^2 ,\nonumber \\ m_{Z'}^2\simeq & {} m_X^2+m_X^2 \xi \left( \xi -s_{\hat{W}} \delta \right) -m_{\hat{Z}}^2\left( \xi -s_{\hat{W}}\delta \right) ^2, \end{aligned}$$

where $m_Z$ must coincide with the experimentally measured value of the *Z* boson mass.

The interactions (relevant for the DM phenomenology) of the $Z,Z'$ with the SM states are described by [247]:

104 $$\begin{aligned} {\mathscr {L}}_{Z/Z',SM}= & {} \overline{f} \gamma ^\mu \left( g_{f_L}^{Z}P_L+g_{f_R}^{Z}P_R\right) f Z_\mu \nonumber \\&+\,\overline{f} \gamma ^\mu \left( g_{f_L}^{Z'}P_L+g_{f_R}^{Z'}P_R\right) f Z'_\mu \nonumber \\&+\,g_W^Z [[W^+ W^-Z]] \nonumber \\&+\,g_W^{Z'} [[W^+ W^-Z']] +g_{hZZ} Z^\mu Z_\mu h \nonumber \\&+\, g_{hZZ'} Z'_\mu Z^\mu h +g_{hZ'Z'} Z'_\mu Z'^{\mu } h, \end{aligned}$$

where:

105 $$\begin{aligned} g_{f_L}^Z= & {} -\frac{g}{c_W}\cos \xi \left\{ T_3 \left( 1+\frac{\omega }{2}\right) \right. \nonumber \\&\left. -\,Q \left[ s_W^2 +\omega \left( \frac{2-t_W^2}{2(1-t_W^2)}\right) \right] \right\} , \nonumber \\ g_{f_R}^Z= & {} \frac{g}{c_W}\cos \xi \left\{ Q \left[ s_W^2+\omega \left( \frac{2-t_W^2}{2(1-t_W^2)}\right) \right] \right\} . \end{aligned}$$

106 $$\begin{aligned} g_{f_L}^{Z'}= & {} -\frac{g}{c_W}\cos \xi \left( T_3 \left[ s_W \tan \delta -\tan \xi \right. \right. \nonumber \\&\left. \left. +\,0.5 {\omega }\left( \tan \xi +{s_W t_W^2 \tan \delta (1-t_W^2)^{-1}}\right) \right] \right. \nonumber \\&\left. + \,Q \left[ s_W^2 \tan \xi -s_W \tan \delta \right. \right. \nonumber \\&\left. \left. +\,0.5 \, t_W^2 \omega (1-t_W^2)^{-1} \left( {\tan \xi -s_W \tan \delta }\right) \right] \right) , \nonumber \\ g_{f_R}^{Z'}= & {} -\frac{g}{c_W}\cos \xi \left\{ Q \left[ s_W^2 \tan \xi -s_W \tan \delta \right. \right. \nonumber \\&\left. \left. +\,0.5\,t_W^2 \omega (1-t_W^2)^{-1}\left( {\tan \xi -s_W \tan \delta }\right) \right] \right\} . \end{aligned}$$

107 $$\begin{aligned} g_W^Z= & {} g c_W \cos \xi \left( 1-\frac{\omega }{2 (c_W^2-s_W^2)}\right) ,\nonumber \\ g_W^{Z'}= & {} -g c_W \sin \xi \left( 1-\frac{\omega }{2 (c_W^2-s_W^2)}\right) . \end{aligned}$$

108 $$\begin{aligned} g_{hZZ}= & {} \frac{m_Z^2}{v_h}\cos \xi ^2 (1+\omega ),\nonumber \\ g_{hZZ'}= & {} 2\frac{m_Z^2}{v_h}\cos \xi ^2\left[ 2 s_W \tan \delta -\tan \xi \right. \nonumber \\&\left. +\,\omega \left( \tan \xi +{s_W t_W^2 \tan \delta (1-t^2_W)^{-1}}\right) \right] ,\nonumber \\ g_{hZ'Z'}= & {} \frac{m_Z^2}{v_h}\cos \xi ^2\left[ \tan ^2\xi +s_W^2 \tan \xi \right. \nonumber \\&\left. - \,\omega \left( 2+\tan ^2 \xi -{s_W^2 t_W^2 \tan ^2 \delta (1-t^2_W)^{-1}}\right) \right] .\nonumber \\ \end{aligned}$$

Here $T_3,\,Q$ are the isospin quantum number and electric charge of the associated SM fermions. The coupling of the DM of various spin allocations, i.e., scalar, fermion and vector, with the two mass eigenstates are respectively given by:

109 $$\begin{aligned} {\mathscr {L}}_\chi= & {} g_X \left( \chi ^{*}\partial _\mu \chi -\chi \partial _\mu \chi ^{*}\right) \left( g_\mathrm{DM}^X Z^\mu +g_\mathrm{DM}^{Z'} Z'^\mu \right) ,\nonumber \\ {\mathscr {L}}_\psi= & {} g_X \overline{\psi }\gamma _\mu \psi \left( g_\mathrm{DM}^X Z^\mu +g_\mathrm{DM}^{Z'} Z'^\mu \right) ,\nonumber \\ {\mathscr {L}}_V= & {} \eta _V^X \left( g_\mathrm{DM}^{Z'} [[VVZ']]+g_\mathrm{DM}^{Z} [[VVZ]]\right) , \end{aligned}$$

with $g_\mathrm{DM}^{Z'}=\frac{\cos \xi }{\cos \delta }$ and $g_\mathrm{DM}^{Z}=-\frac{\sin \xi }{\cos \delta }$. As evident, in the physical basis, the DM is connected to the SM sector by two s-channel mediators, the *Z* and the $Z'$.

The DM relic density is determined by annihilation processes into the SM fermion pair final states, *WW* and $Z(Z')h$, induced by s-channel exchange of the mediators, and *ZZ*, $Z'Z$ and $Z'Z'$, induced by t-channel exchange of a DM state. The corresponding rates can be straightforwardly derived from the cases of $Z/Z'$ portals so won’t be re-discussed in detail here. Similar to the scenarios already described, the DM DD relies on the SI interactions, which induces a scattering cross-section written, for the case of proton, as:

110 $$\begin{aligned}&\sigma _{\chi p/\psi p/V p}^\mathrm{SI}=\frac{\mu _{\chi p/\psi p/V p}^2 c_X^2}{\pi }{\left[ b_p \frac{Z}{A}+b_n \left( 1-\frac{Z}{A}\right) \right] }^2,\nonumber \\&\quad \mathrm{where}\ b_p=2 b_u+b_d,\quad b_n=b_u+2 b_d,\nonumber \\&\quad \mathrm{and}\ b_f=\frac{g_{DM}^Z\left( g_{f_L}^Z+g_{f_R}^{Z}\right) }{2 m_Z^2} +\frac{g_{DM}^{Z'}\left( g_{f_L}^{Z'}+g_{f_R}^{Z'}\right) }{2 m_{Z'}^2}.\nonumber \\ \end{aligned}$$

Here $\mu _{\chi p/\psi p/V p}$, as already defined, denotes reduced mass of the concerned WIMP-proton system. $c_X$ encodes the DM couplings $g_X^2$ (for complex scalar and fermionic DM) or $\eta _V^X$ (for vectorial DM) and other eventual overall factors, depending on the kind of DM candidate.

**Fig. 22:**
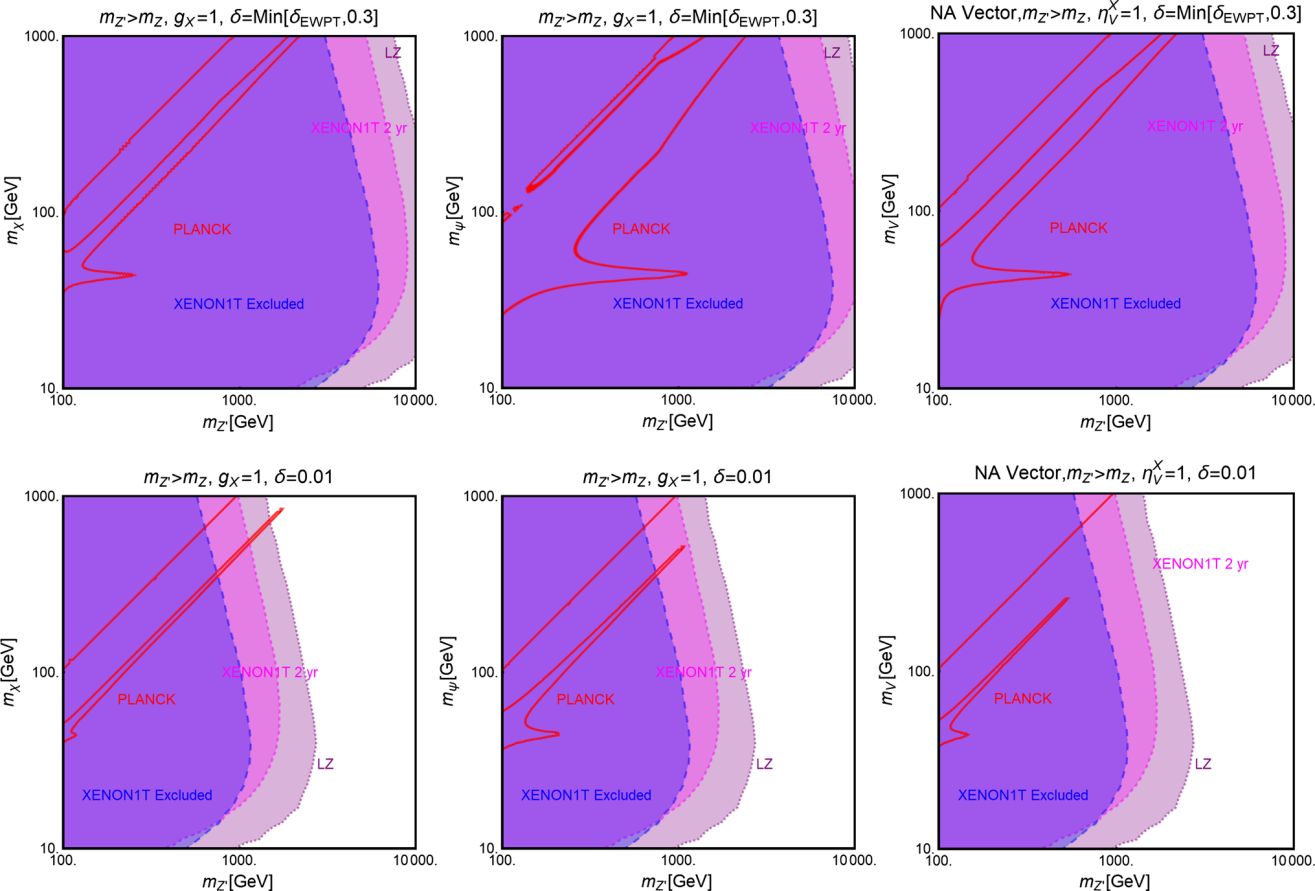
Combined constraints in the relevant bi-dimensional planes $(m_{Z'},\,m_\chi )$, $(m_{Z'},\,m_\psi )$ and $(m_{Z'},\,m_V)$ for the scalar (left column), fermionic (middle column) and non-Abelian vectorial (right column) DM, respectively, interacting with a $Z'$, kinetically coupled with the SM *Z* boson. In the top-row plots the kinetic mixing parameter $\delta$ has been set to the maximal value, as a function of $m_{Z'}$, consistent with the EWPT constraints while for the bottom-row plots $\delta$ has been set to a constant value of 0.01. We set $g_X=1$ for all these plots. In this figure the red curve represents the contour of correct DM relic density. The blue region is excluded by the current constraints from XENON1T while the magenta and purple regions would appear excluded in the absence of signals from XENON1T (after 2 years of exposure) and LZ, respectively

The interplay between the DM relic density and DD is shown, for the various spin assignations of the DM, in Fig. 22. We have considered two assignations of the kinetic mixing parameter, the first one is derived from the present limit of the EWPT (i.e., taking the equal sign in Eq. (102)) while the second one corresponds to a constant value $\delta =0.01$. This last choice is inspired by models in which the kinetic mixing parameter is radiatively generated upon integrating out heavy degrees of freedom charged under both the new *U*(1) gauge group and the $U(1)_Y$ of the SM [315, 319].

By comparing the outcome of Fig. 22 with the scenarios where the $Z'$ is directly coupled to the SM fermions, we notice that DD probes a more limited region of the parameter space. This is because the scattering cross-section depends on coupling suppressed by the smallness of the parameter $\delta$. However, this kind of suppression affects also the DM annihilation processes, ad exception of the $Z'Z'$ final state so that a strong tension with the experimental constraints, for example similar to the SSM (see Sect. 8.2), still persists. We, indeed notice that the thermal DM is excluded for masses below the TeV scale unless small values, $\mathscr {O}\sim (0.01)$, of the kinetic mixing parameter are taken. Even in such a case, the correct relic density is achieved only at the pole $m_{Z'}/2$ or for $m_{\chi ,\psi ,V} > m_{Z'}$, where annihilation into $Z'Z'$ is accounted without relying on the kinetic mixing parameter. These setups will nevertheless be excluded in the absence of signals in the next generation multi-TON experiments.

As explicitly indicated in the plots of Fig. 22 that we have considered only the case $m_{Z'}>m_Z$. The opposite regime (often dubbed as dark photon) would also be feasible, although the constraints on $\delta$ would be even stronger because of Eq. (102) and additional constraints from muon $(g-2)$ and parity violating effects in the atomic physics [247] as well as from low-energy colliders [320]. We have checked that the low mass $Z'$ regime is substantially excluded by the current DD limits, unless DM masses below the sensitivity reach of DD experiments are considered. Thus, we have not reported it explicitly.

## DM (partially) charged under $SU(2) \times U(1)$

All the models considered until now have assumed the DM to be a SM singlet. In this section we will, instead, consider the case in which the DM particle has non-trivial quantum numbers under the EW component of the SM gauge group. A rather straightforward realization consists into considering the DM as the lightest neutral component of a *SU*(2) multiplet. Extensive discussions can be found in Ref. [321] and Refs. [169, 170] for scalar and fermionic DM, respectively. These models cannot strictly be classified as Dark portals since the DM relic density is mostly set by annihilations processes into *Z* and *W* boson which are determined by the gauge couplings between the latter and the components of the DM multiplet. These are, nevertheless, very interesting and predictive models, having the DM mass (for this reason they are dubbed as “Minimal DM”) as the only free parameter. For these models the DM relic density is typically strongly suppressed by efficient annihilation processes into $W^{+}W^{-}$ and *ZZ* final states unless the DM is sufficiently heavy, possibly above the TeV scale. For these high values of the DM masses the treatment presented in Sect. 2 should be refined since, besides co-annihilation processes with the other states belonging to the DM multiplet, one should also account for Sommerfeld enhancement effects [170, 171, 321] and even effects from bound states formation [322]. These effects are typically associated with an enhancement, with respect to the conventional treatment, of the DM annihilation cross-section so that the correct DM relic density is reached, for some realizations of the Minimal DM, for masses even of the order of 10 TeV. Concerning detection strategies the best probe, at the moment, is represented by ID [323]. On the contrary, scattering cross-section on nucleons are typically rather suppressed [324–326] and hence, DD probes are not very efficient. Further, given the high values of the DM masses corresponding to the correct DM relic density, the collider searches could, similarly, hardly probe these scenarios in the near future [326].

As already mentioned that the minimal DM scenarios, despite being phenomenologically very interesting, do not strictly belong the category of Dark Portals. Moreover, they are not the best target for direct DM searches. For these reasons they will not be discussed here in further detail.

Models more similar to Dark portals, achieving the correct DM relic density at lower values of the DM mass, consist of the case in which the DM is the mixture of the neutral component of a *SU*(2) multiplet and a SM *SU*(2) singlet. In such a case the coupling of the DM with the gauge bosons is suppressed through the mixing between the *SU*(2) singlet and the multiplet(s). Similar to Dark portals, annihilation into the SM fermions, through s-channel mediation of the SM Higgs (or the *Z* boson), can play a relevant role for the DM relic density. Viable relic density is potentially achieved also for the DM masses of the order of a few hundreds GeV. The most popular example of these kind of scenarios is represented by Supersymmetric theories where the DM candidate, the lightest neutralino, is indeed a mixed state comprised of the bino (SM singlet), the Wino (electrically neutral component of a *SU*(2) triplet) and two higgsinos (electrically neutral components of *SU*(2) doublets).

We also remark that these kind of constructions represent renormalizable completions of the SM Higgs and Z portal models discussed in the Sect. 7. As will be shown subsequently, the phenomenology of these more realistic constructions differs from the simple SM Higgs and Z-portals in particular because of the presence of additional states belonging to the DM sector which can affect the DM relic density through co-annihilations.

In the following subsection we will review some representative cases of the general scenarios discussed above. We will first review the Inert Doublet Model (IDM) [327–335] in which the DM belongs to a scalar *SU*(2) doublet coupled with the SM Higgs sector. For what concerns the case of mixing between *SU*(2) singlets and multiplet we will consider the simplest possibility, the so called Singlet-Doublet models [173], specializing to the cases of Dirac [177] and Majorana [176, 336–338] fermionic DM. For the discussion of cases of mixing with higher multiplets we refer, for example, to Refs. [339–342].

### Inert doublet model

The IDM enlarges the SM Higgs sector with an additional doublet, giving rise to the following scalar potential:

111 $$\begin{aligned} V= & {} \mu _1^2 |H_1|^2+\mu _2^2 |H_2|^2+\lambda _1 |H_1|^4+\lambda _2 |H_2|^4 \nonumber \\&+\,\lambda _3 |H_1|^2 |H_2|^2+\lambda _4 |H_1^{\dagger }H_2|^2+\frac{\lambda _5}{2}\left[ (H_1^{\dagger }H_2)^2+\text{ h.c. } \right] ,\nonumber \\ \end{aligned}$$

with $H_1$ and $H_2$ being, respectively, the SM and the new scalar doublet. $\mu _i$ and $\lambda _i$ are real parameters. The additional Higgs doublet is odd under a discrete ${\mathbb {Z}}_2$ symmetry which forbids its direct couplings with the SM fermions. In addition, the new doublet does not acquire VEV when the $SU(2)_L \times U(1)_Y$ symmetry is spontaneously broken. After the EWSB, four stable mass eigenstates are obtained: two electrically charged, labeled as $H^{\pm }$, and two electrically neutral, labeled as $H^0$ and $A^0$. Their masses are given by:

112 $$\begin{aligned} m_{H^{\pm }}^2= & {} \mu _2^2+\frac{\lambda _3 v_h^2}{2},\nonumber \\ m_{H^0}^2= & {} \mu _2^2+\frac{1}{2}(\lambda _3+\lambda _4+\lambda _5)v_h^2,\nonumber \\ m_{A^0}^2= & {} \mu _2^2+\frac{1}{2}(\lambda _3+\lambda _4-\lambda _5)v_h^2. \end{aligned}$$

A viable DM candidate, thus, is achieved as one of the states between $H_0$ and $A_0$ by setting the quartic couplings $\lambda _3,\lambda _4$ and $\lambda _5$ appropriately. For simplicity, we will restrict to the case when $H^0$ is the DM candidate. The quartic couplings are also constrained by the vacuum stability, which imposes (at tree level):

113 $$\begin{aligned} \lambda _{1,2}> & {} 0, \nonumber \\ \lambda _3, \lambda _3+\lambda _4-|\lambda _5|> & {} -2 \sqrt{\lambda _1 \lambda _2}, \end{aligned}$$

and, $\lambda _i < 4 \pi$ from perturbativity.

As will be shown subsequently that the most relevant DM observables depend on the combination of couplings $\lambda _L=\frac{1}{2}(\lambda _3+\lambda _4+\lambda _5)$. We will thus adopt, as free parameters for our analysis, $\lambda _L$ as well as the masses of the new Higgs states. The constraints from vacuum stability and perturbativity are then translated into bounds on $m_{H^0},m_{A^0},m_{H^{\pm }}$ through Eq. (112). The latter are also constrained by the EWPTs. Finally, a lower bound of $79.3\,\text{ GeV }$ on the mass of the charged Higgs is applied from LEP [137].

The direct DM detection relies on a SI interaction whose cross-section is substantially analogous to the one used for the Higgs/spin-0 portal:^35^ (according to the usual convention we quote the DM scattering cross-section on protons):

114 $$\begin{aligned} \sigma _{H^0p}^\mathrm{SI}=\frac{\mu _{H^0}^2}{4\pi }\frac{m_p^2}{m_{H^0}^2 m_h^4}\lambda _L^2 \left[ f_p \frac{Z}{A}+f_n \left( 1-\frac{Z}{A}\right) \right] ^2, \end{aligned}$$

where $m_p$ is the mass of the proton, $\mu _{H^0}$ is the reduced mass of the DM-proton system and the coefficients $f_p$ and $f_n$ are the ones defined in Eq. (35), and $A\,(Z)$, is the atomic mass (number) of the target nucleus.

Concerning the DM relic density, there are similarities with the SM Higgs portal only at the low DM masses, namely below the mass $m_W$ of the SM *W* boson. Here the DM relic density is mostly determined by annihilation into SM fermions mediated by the *h* boson. As the mass of the DM approaches towards $m_W$, the annihilation into *WW* finals states enhances, with respect to the SM Higgs portal model discussed in Sect. 7, by t-channel diagrams involving the exchange of $H^\pm$ and contact four field interactions between a $H^0$ and a $W^{\pm }$ pairs. The contributions of these diagrams to the cross-section depend on the $SU(2)_L$ gauge coupling *g* rather than the quartic couplings of the scalar potential. A strong enhancement of the cross-section is already produced for $m_{H^{0}} \lesssim m_{W}$ by the process $H^0 H^0 \rightarrow W W^{*} \rightarrow Wf \overline{f}^{'}$ (this process is illustrated in detail, including an analytical expression of its amplitude, in Ref. [328]).

For positive values of $\lambda _L$ the correct DM relic density is achieved for DM masses around 70 GeV while for higher values of the DM masses, the annihilation rate into *WW* is so strong that the DM turns out to be underabundant.^36^ For $\lambda _L<0$, destructive interference among the different diagrams contributing to the annihilation rate into *WW* final state might occur so that the region of viable DM can be extended, according to the values of $\lambda _L$, $m_{A^0}$ and $m_{H^{\pm }}$, towards the DM masses slightly above 100 GeV [329].

**Fig. 23:**
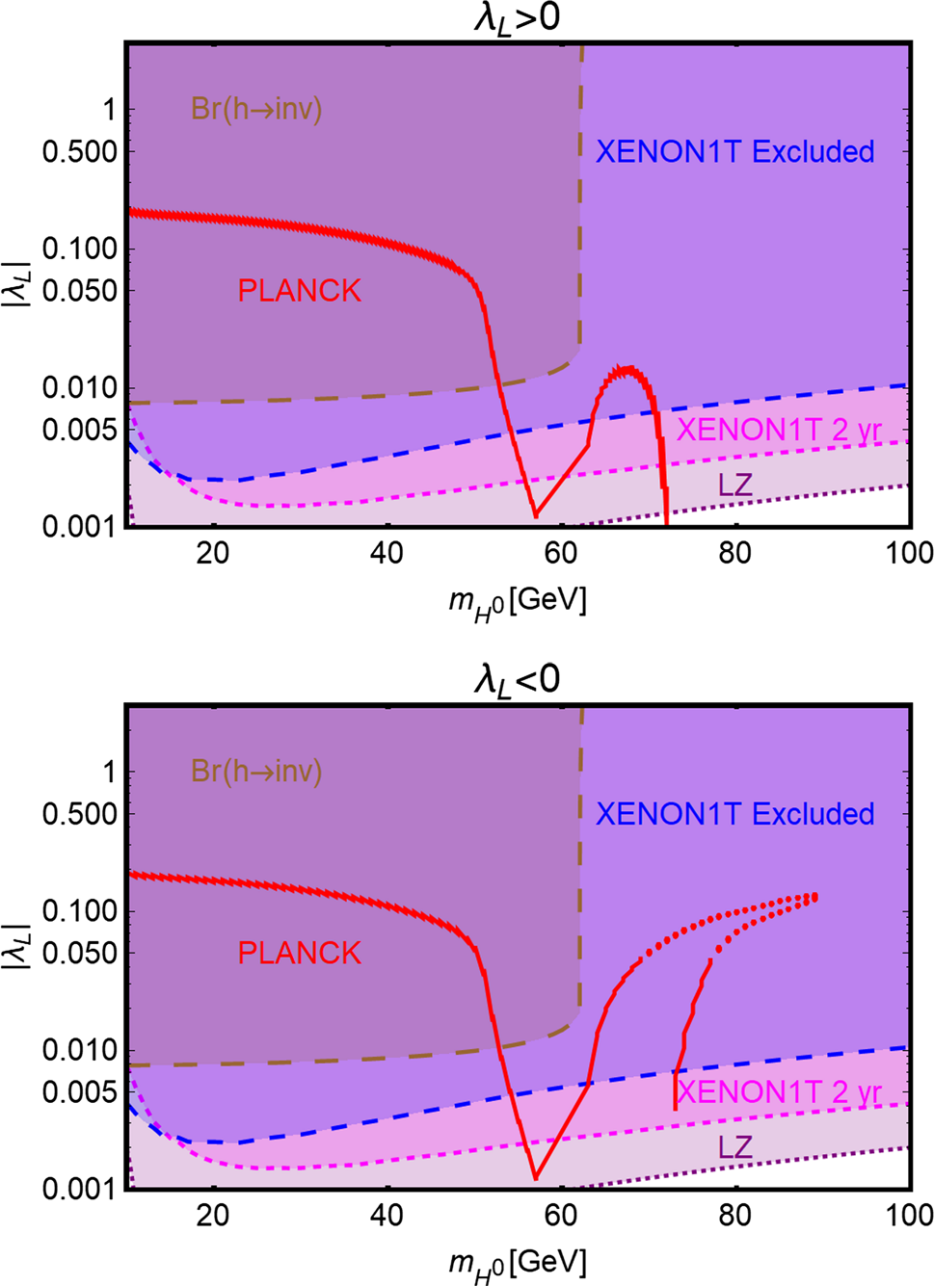
Summary of constraints for the IDM model in the light DM mass regime, in the bi-dimensional plane $(m_{H^0},|\lambda _L|)$ for $\lambda _L$>0 (upper panel) and $\lambda _L<0$ (lower panel). The color coding is the same as used for the SM Higgs portal model in Sect. 7: the red curve represents the iso-contour of the correct DM relic density. The blue, magenta and purple regions are, respectively, the XENON1T excluded region and the projected exclusions by XENON1T (assuming 2 years of exposure time) and LZ. The brown region is excluded by the current experimental determination of the SM Higgs invisible branching fraction

An overview of the DM phenomenology of the IDM in the low DM mass regime is presented in Fig. 23. The two panels of the figure, corresponding to the cases of $\lambda _L>0$ and $\lambda _L<0$ report, similar to the case of SM Higgs portal model, the combined constraints from the DM relic density, DD including the projected sensitivities of XENON1T and LZ and, invisible branching fraction of Higgs boson, in the bi-dimensional plane $m_{H^0},|\lambda _L|$. Moreover, we assumed a sizable mass splitting between the DM and the extra scalar states in order to forbid co-annihilation effects. As already anticipated, for $m_{H^0} \lesssim m_h/2$ the outcome is substantially analogous to the SM Higgs portal model; the viable DM region is excluded by the combination of DD and Higgs invisible branching fraction constraints, except for the pole $m_{H^0} \sim m_h/2$ which will be fully probed by the next generation DD experiments. For $m_{H^0}>m_h/2$, an additional viable region is present corresponding to a scenario where the DM relic density is mostly determined by the annihilation process $H^0 H^0 \rightarrow Wf \overline{f}^{'}$. Given the dependence of the corresponding rate on the gauge coupling, the correct DM relic density is achieved for very low values of $\lambda _L$, passing the current DD constraints.

As the DM mass increases further the annihilation into two on-shell *W* (as well as into *Z* pair) bosons becomes accessible. An efficient annihilation channel is represented by $H^0 H^0 \rightarrow hh$ process as well. These rates are in general very efficient to account for the correct DM relic density. It can nevertheless be achieved for relatively high DM masses provided that some specific requirements on the model parameters are met. The annihilation cross-section into *hh* is proportional to the coupling $\lambda _L$, a sufficient suppression is then achieved by going to the limit $\lambda _L \rightarrow 0$. Interestingly, this would also imply a suppression of the DM scattering rate on nucleons which is also proportional to the same coupling. More contrived is the case of the annihilation into $W^+ W^-$ and *ZZ* final states. The corresponding cross-sections normally receive contributions depending on the coupling $\lambda _4+\lambda _5$ (in the case of $W^+ W^-$) and $\lambda _5$ (for *ZZ*) which are responsible of enhancement factors of

115 $$\begin{aligned}&\frac{\alpha _W^2}{m_Z^2}{\left( m_{A^0}^2-m_{H^0}^2\right) }^2\quad \mathrm{and} \nonumber \\&\frac{\alpha _W^2}{m_Z^2} \left[ {\left( m_{A^0}^2-m_{H^0}^2\right) }^2+{\left( m_{H^{\pm }}^2-m_{H^0}^2\right) }^2\right] , \end{aligned}$$

respectively.

It is thus possible to suppress the annihilation cross-sections into gauge bosons by taking almost degenerate $m_{H^0}$, $m_{A^0}$ and $m_{H^{\pm }}$ states. In the limit of $m_{H^0}$ $\sim m_{A^0}$ $\sim m_{H^{\pm }}$, the DM annihilation cross-section into gauge bosons scales as $\alpha _W^2/m_{H^0}^2$ and the thermally favored values are matched for masses of the order of 500 GeV [327]. In this nearly degenerate mass scenario an interesting additional phenomenon occurs. If only self annihilations of the DM are taken into account then the expected value of its abundance is typically small (i.e., much below the thermally favored value). However, since the pseudoscalar and the charged Higgs belong to the same doublet and share similar cross-sections, they get decoupled after the DM freeze-out. Therefore, despite of a small DM abundance shortly after the freeze-out, an eventual enhancement of the DM abundance appears when both the pseudoscalar and charged Higgs fields decay back to the DM pair. This is the mechanism for getting the correct DM relic density as explained in detail in Ref. [344].

**Fig. 24:**
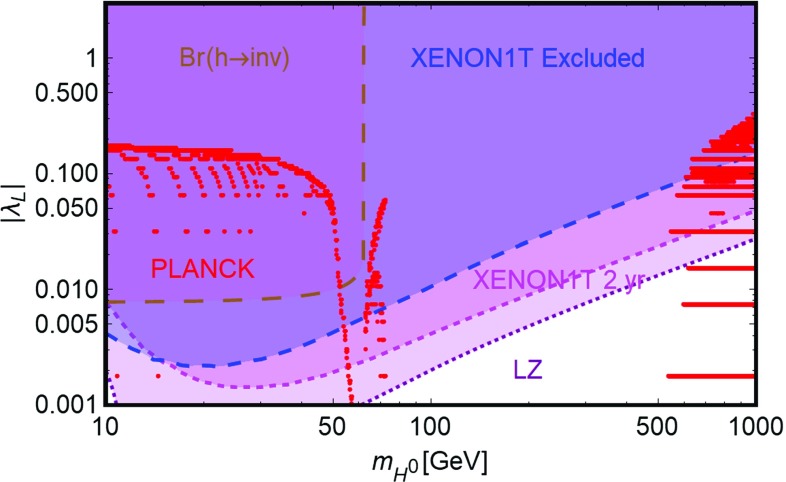
Model points with the correct DM relic density (red points) obtained from a scan over the parameters of the IDM (see main text for details) in the bi-dimensional $m_{H^0},|\lambda _L|$ plane. The blue, magenta and purple regions are excluded by the current bounds from XENON1T and, projected sensitivities by XENON1T and LZ, respectively. The brown region is excluded by the constraint on the invisible branching fraction of the SM Higgs boson

In order to properly investigate the effects described above, we have conducted a scan on the parameters $m_{H^0}$, $m_{A^0}$, $m_{H^{\pm }}$ and $\lambda _L$ over the following ranges:

116 $$\begin{aligned} m_{H^0}\in & {} \left[ 10,1000\right] \,\text{ GeV }, \nonumber \\ (m_{A^0}- m_{H^0})\in & {} \left[ 1,100\right] \,\text{ GeV }, \nonumber \\ (m_{H^{\pm }}- m_{H^0})\in & {} \left[ 1,100\right] \,\text{ GeV }, \quad \text{ only } \text{ for }\ m_{H^0}>80\,\text{ GeV },\nonumber \\ |\lambda _L|\in & {} [10^{-6},1]. \end{aligned}$$

Notice that now we have extended the scan also for the low DM mass regime including the possibility, neglected in the case represented by Fig. 24, of mass degeneracy between $m_{H^0}$, $m_{A^0}$, $m_{H^{\pm }}$, so that co-annihilation processes can take place.

The model points featuring the correct DM relic density have been reported in Fig. 24 in the bi-dimensional plane $(m_{H^0},|\lambda _L|)$ together with the existing limits and projected sensitivities from direct DM detection and Higgs invisible branching fraction. As evident that the presence of co-annihilations does not allow to evade the strong constraints in the low DM mass regime. On the contrary, a new viable DM region is present for $m_{H^0}\gtrsim 500\,\text{ GeV }$ where although the present and expected future limits are effective but still there will be a sizable amount of model points that would survive even in case of absence of signals at LZ.

We emphasize that in this high DM mass regime, co-annihilations are responsible for producing the correct DM relic density. The DM annihilations into gauge bosons are rather strong to get the correct DM relic density. That said, today these co-annihilations are absent since the co-annihilating particles first got decoupled from the thermal bath and then decayed back into DM. Therefore, we are left with the DM self annihilations into gauge bosons which are of the order of $10^{-25} - 5\times 10^{-26}$ $\mathrm{cm}^3\, \mathrm{s}^{-1}$. This falls perfectly within the sensitivity reach of Cherenkov Telescope Array (CTA). Indeed, it has been shown that such telescope array provides the most effective probe for this model, being possibly able to rule out the model till DM masses of 2.8 TeV [344, 345] (see also [346–348] for possible indirect signals in the Inert Doublet Model).

### Singlet-doublet DM

We will discuss in the following texts two scenarios of fermionic DM originating from the mixing between a SM singlet and the electrically neutral component of a $SU(2)_L$ doublet. As already pointed out that this kind of setup allows for renormalizable couplings between the DM and Higgs and gauge bosons, in particular the *Z*, and thus, represents a theoretical framework where the SM portal models discussed in Sect. 7 can be embedded. We also remark that the new fermions which couple with the Higgs should belong to a real representation (i.e., Majorana fermions) or form vector-like pairs, since chiral fermions, with masses originating from the EWSB, are experimentally strongly disfavored [349].

#### Dirac DM

The Dirac fermionic Singlet-Doublet model is realized by adding to the SM particle spectrum vector like fermions $L_{L,R}$ and $N^{'}_{L,R}$, transforming as $SU(2)_L$ doublets (with hypercharge 1 / 2) and singlets (with hypercharge 0), respectively. The relevant phenomenology is described by the following Lagrangian [177]:

117 $$\begin{aligned} {\mathscr {L}}= & {} -M_L \overline{L}_L L_R-M_N \overline{N}^{'}_L N_R \nonumber \\&-\,y_1 \overline{L}_L \widetilde{H} N^{'}_R-y_2 \overline{L}_R \widetilde{H} N_L^{'}+\text{ h.c. }, \end{aligned}$$

where, as usual for simplicity we have omitted the kinetic terms. $\widetilde{H}$ is the conjugated Higgs field. A discrete ${\mathbb {Z}}_2$ symmetry, under which the new fermions are odd, is assumed to avoid couplings of the latter (through the Higgs) with the SM fermions which in turn guarantees the cosmological stability of the DM. The DM candidate is the lightest of the two neutral Dirac fermionic states arising from the bi-diagonalization procedure,

118 $$\begin{aligned} \left( \begin{array}{cc} m_{\psi _1} &{} 0 \\ 0 &{} m_{\psi _2} \end{array} \right) =U^{\dagger }_L M U_R, \end{aligned}$$

of the mass matrix:

119 $$\begin{aligned} M=\left( \begin{array}{cc} M_N &{} \frac{y_2 v_h}{\sqrt{2}} \\ \frac{y_1 v_h}{\sqrt{2}} &{} M_L \end{array} \right) , \end{aligned}$$

where:

120 $$\begin{aligned} U_{L,R}=\left( \begin{array}{cc} \cos \theta _{L,R} &{} \sin \theta _{L,R} \\ -\sin \theta _{L,R} &{} \cos \theta _{L,R} \end{array} \right) , \end{aligned}$$

with:

121 $$\begin{aligned} \tan 2 \theta _L= & {} \frac{\sqrt{2}v_h \left( M_N y_1+M_L y_2\right) }{M_L^2-M_N^2+\frac{v_h^2}{2}\left( y_1^2-y_2^2\right) },\nonumber \\ \tan 2 \theta _R= & {} \frac{\sqrt{2}v_h \left( M_N y_2+M_L y_1\right) }{M_L^2-M_N^2+\frac{v_h^2}{2}\left( y_2^2-y_1^2\right) }, \end{aligned}$$

so that the two physical masses, with $c{\theta _L},\,s{\theta _L}$ $\equiv \cos \theta _L,\sin \theta _L$ are given by:

122 $$\begin{aligned} m_{\psi _1}^2= & {} \frac{c^2\theta _L\left( M_N^2+y_2^2 v_h^2/2\right) -s^2\theta _L \left( M_L^2+y_1^2 v_h^2/2\right) }{c^2 \theta _L-s^2 \theta _L},\nonumber \\ m_{\psi _2}^2= & {} \frac{s^2\theta _L\left( M_N^2+y_2^2 v_h^2/2\right) -c^2\theta _L \left( M_L^2+y_1^2 v_h^2/2\right) }{s^2 \theta _L-c^2 \theta _L}.\nonumber \\ \end{aligned}$$

The physical spectrum of the theory features also a charged state $\psi ^{\pm }$ with mass $m_{\psi ^{\pm }}\simeq M_L$.

The model features four free parameters, the two masses $M_{N},M_{L}$ and the couplings $y_1,y_2$. As can be easily realized that the couplings of the DM with the SM fields essentially depends on the angles $\theta _L$ and $\theta _R$ which determine the amount of its component charged under $SU(2)_L \times U(1)_Y$.

The DM relic density is determined by a large variety of annihilation processes, including fermion pair final states (from s-channel exchange of the *Z* and Higgs boson), $W^+ W^-$ final state (from s-channel exchange of the *Z* / *h* boson and t- channel exchange of $\psi ^{\pm }$), *ZZ* final state (from s-channel exchange of Higgs boson and t-channel exchange of $\psi _{1,2}$) and finally *Zh* final state (from s-channel exchange of the *Z* boson and t-channel exchange of $\psi _{1,2}$). To these, one should possibly add co-annihilation processes in analogous combination of the final states in case the additional neutral and charged fermions have masses close enough to the same of the DM.

The most relevant contribution to the DM scattering processes on nucleons comes from SI interaction mediated by the *Z* boson. The corresponding cross-section is very similar to the one written for the SM *Z*-portal model^37^

123 $$\begin{aligned} \sigma _{\psi _1 p}^\mathrm{SI}= & {} \frac{g^2}{16 \cos ^2 \theta _W}\left( \sin ^2 \theta _L +\sin ^2 \theta _R\right) ^2\frac{\mu _{\psi _1 p}^2}{\pi m_Z^4}\nonumber \\&\times \, {\left[ V_u^Z \left( 1+\frac{Z}{A}\right) +V_d^Z \left( 2-\frac{Z}{A}\right) \right] }^2, \end{aligned}$$

where $V_u^Z$ and $V_d^Z$ are the vectorial couplings of the SM *Z*-boson with up and down-type quarks.

**Fig. 25:**
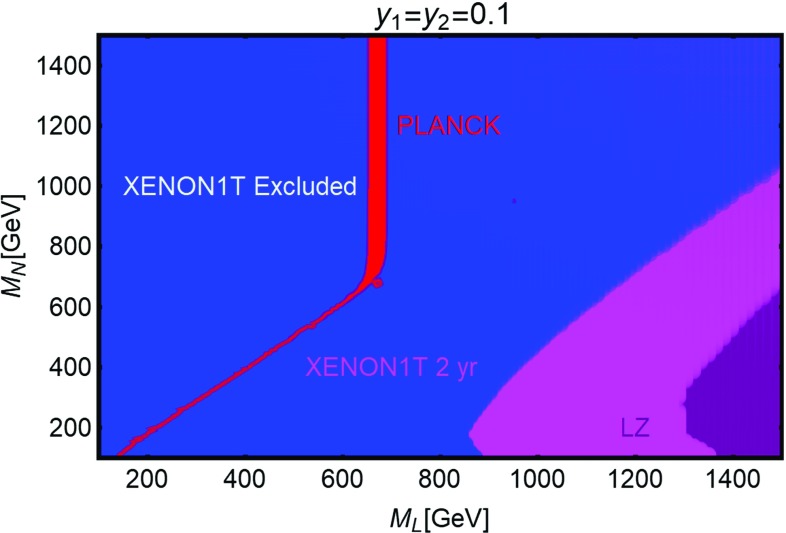
Combined relic density and DD constraints for the Singlet-Doublet Dirac fermion DM model in the bi-dimensional plane $(M_L,M_N)$ by fixing $y_1=y_2=0.1$. The correct DM relic density is achieved, through thermal freeze-out, along the red contour. The blue region is excluded by limits on SI interactions from the XENON1T experiment while the magenta and purple regions will be excluded in the case of absence of signals at XENON1T, assuming 2 years of exposure time, and LZ, respectively

Similar to all the models discussed in this work, we will provide an overview of the interplay between the requirement of the correct DM relic density and the experimental constraints, mostly from DD, by considering a bi-dimensional plane in the mass parameters $(M_L,M_N)$ in this case and, a fixed assignation of the couplings. This kind of study is presented in Fig. 25. Contrary to most of the previously studied cases we have not chosen $\mathscr {O}(1)$ values of the couplings here but a sensitively lower value, i.e., $y_1=y_2=0.1$. This is because the possibility of vector-like fermions having $\mathscr {O}(1)$ couplings with Higgs is theoretically rather contrived since it would result potentially dangerous effects on the stability of the scalar potential [179, 350, 351].

The behavior of the contour of the correct DM relic density in the bi-dimensional plane $(M_L,M_N)$ is explained as follows. In order to match the DM annihilation cross-section with the thermally favored value, a sizable mixing between the singlet and the doublet components is needed, implying $M_N \sim M_L$ (in this regime co-annihilations are also important). The correct DM relic density can be obtained for a maximal value of the DM mass of approximately 733 GeV [177]. At this value the correct relic density can be achieved when the DM mostly coincides with the neutral component of a *SU*(2) doublet (hence co-annihilating with its almost degenerate charged partner) and, is guided by gauge interactions of the DM with the *Z* and the *W* bosons. Note that a similar effect concerning the issue of mass degeneracy of the DM multiplet, as the one discussed in the previous subsection, occurs also in this case. Here, the presence of a state nearly mass degenerate with the DM is associated with an increase of the DM relic density, rather than a decrease as happens in the conventional co-annihilation paradigm.

Because of the Dirac nature of the DM, the associated sizable interactions with the *Z* boson lead to an extremely high SI cross-section, already excluded by far by the present experiments. This is confirmed by the outcome of Fig. 25 which shows that the region of viable DM relic density is completely excluded by the XENON1T constraint alone.

**Fig. 26:**
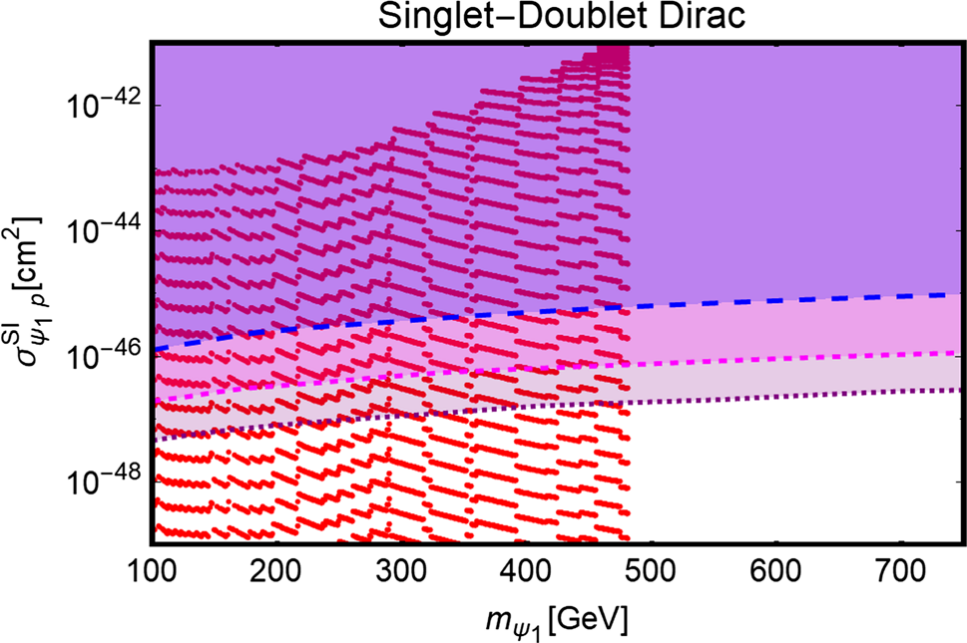
Model points (red) for the Singlet-Doublet Dirac fermion DM model, generated through a scan over the $M_N,M_L$, $y_1,y_2$ parameters (see main text), having the correct DM relic density in the bi-dimensional plane $(m_{\psi _1},\sigma _{\psi _1 p}^\mathrm{SI})$. The blue, magenta and purple regions correspond to the present as well as projected exclusions by XENON1T and LZ, respectively

In order to evade DD constraints one should instead approach the pure singlet limit, i.e., $\left( \sin ^2 \theta _L+\sin ^2 \theta _R\right) \ll 1$. This would, however, imply a very suppressed pair annihilation cross-section for the DM. The correct DM relic density can nevertheless be achieved through the pair annihilations of the $\psi _2$ and $\psi ^{\pm }$ states, provided that they are almost degenerate in masses with the DM. This would require $M_N \sim M_L$ and to avoid an enhancement of $\left( \sin ^2 \theta _L+\sin ^2 \theta _R\right)$, $y_{1,2} \ll 1$.

The aforesaid features cannot be represented through a simple bi-dimensional plot. Hence, to investigate those characteristics, similar to the previous subsection, we have conducted a parameter scan of the four free parameters $M_N,\,M_L$, $y_1,\,y_2$ over the following ranges:

124 $$\begin{aligned} M_N,\,M_L\in & {} \left[ 100,800\right] \,\text{ GeV },\nonumber \\ y_{1},\,y_2\in & {} [10^{-6},10^{-1}]. \end{aligned}$$

The results of our analysis are reported in Fig. 26 in the bi-dimensional plane $(m_{\psi _1},\sigma _{\psi _1}^\mathrm{SI})$. We have reported there the model points reproducing the correct DM relic density and compared them with the current and anticipated exclusions by XENON1T and LZ, respectively.

As can be seen from the figure that values of the DM scattering cross-section span over a wide range of values. The highest ones, which can be even above $10^{-42}\,{\text{ GeV }}^2$, correspond to the case of a highly mixed DM. In such cases the interactions of the DM with the *Z* boson are of similar size as expected when they are coupled via ordinary gauge couplings and are, by far, excluded by DD. At the same time, model points with the correct DM relic density are obtained also for the case of a almost pure singlet-like DM with $y_1,y_2 \sim 10^{-6}$, implying negligible *Z*-mediated scattering rate, even surviving upon the absence of signals at LZ. For these points the DM relic density is entirely determined by the (co)annihilation processes of the quasi mass degenerate doublet partner. Notice, that the viable points are present only for $m_{\psi _1} \lesssim 500\,\text{ GeV }$. Above this value, all the points with the correct DM relic density are characterized by $M_L < M_N$, corresponding to a doublet-like DM. These points cluster at very high values of the DM scattering cross-section, beyond the range reported in the *y*-axis, and thus, are largely ruled-out by DD.

The presence of extra fermions besides the DM would in principle offer collider tests for the scenario under consideration. The heavier neutral fermion $\psi _2$ and $\psi ^{\pm }$ could indeed be produced through EW processes at colliders, followed by subsequent decay into the DM and an on- or off-shell gauge boson. However, the strong bounds form direct DM detection can be evaded only through a very compressed spectrum for the new fermions. Furthermore, the decay rate of the heavier fermions into the DM is suppressed when the DM is mostly singlet-like. This, as already discussed, is a way to evade DD constrains. The only possible detection prospects, compatible with constraints from the DM phenomenology, would be represented by the detection of the charged fermion $\psi ^{\pm }$, stable at the collider scales, giving large missing transverse momentum with charge tracks.

#### Majorana DM

The Majorana version of the Singlet-Doublet DM model is described by the following Lagrangian:

125 $$\begin{aligned} {\mathscr {L}}= & {} -\frac{1}{2}M_N N^{'\,2}-M_L L_L L_R \nonumber \\&-\,y_1 L_L H N^{'}-y_2 L_R \widetilde{H} N^{'}+\text{ h.c. }, \end{aligned}$$

where we have assumed the definition:

126 $$\begin{aligned} L_L=\left( \begin{array}{c} N_L \\ E_L \end{array} \right) ,\quad L_R=\left( \begin{array}{c} -E_R \\ N_R \end{array} \right) , \end{aligned}$$

As will be pointed out subsequently that the Singlet-Doublet model with a Majorana fermion DM can be interpreted as simplified limit of some specific SUSY realizations. To make this comparison easier, we have assigned to $L_L$ and $L_R$ fields the same set of quantum numbers as the higgsinos $\widetilde{H}_d$ and $\widetilde{H}_u$ (notice that this choice of quantum assignments is different from the case studied in the previous subsection). Further note that, given the transformation properties of the fields $N^{'},\,L_L,\,L_R$ under the discrete ${\mathbb {Z}}_2$ symmetry, only one between the signs of the mass term $M_L$ and of the couplings $y_1$ and $y_2$ is physical [340]. We will assume, in our analysis, positive sign for $M_L$ and assign freely the signs of $y_{1,\,2}$.

Contrary to the previous case, the mass matrix of the electrically neutral new sector is now a $3 \times 3$ matrix,

127 $$\begin{aligned} M=\left( \begin{array}{ccc} M_N &{} \frac{y_1 v_h}{\sqrt{2}} &{} \frac{y_2 v_h}{\sqrt{2}} \\ \frac{y_1 v_h}{\sqrt{2}} &{} 0 &{} M_L \\ \frac{y_2 v_h}{\sqrt{2}} &{} M_L &{} 0 \end{array} \right) , \end{aligned}$$

which is diagonalized through a unitary transformation leading to three (Majorana) mass eigenstates:

128 $$\begin{aligned} \psi _i=N^{'} U_{i1}+N_L U_{i2}+N_R U_{i3}, \end{aligned}$$

the lightest of which, $\psi _1$, is the DM candidate. The mass spectrum of the new states is again completed by a electrically charged Dirac fermion $\psi _{\pm }$ with mass $m_{\psi _{\pm }}\approx M_L$. The DM and the other neutral states interact with the SM states through s-channel mediation of the *Z* and Higgs bosons. The associated couplings are given by:

129 $$\begin{aligned} g^V_{Z\psi _i \psi _j}= & {} c_{Z\psi _i \psi _j}-c^{*}_{Z\psi _i \psi _j} ,\quad g^A_{Z\psi _i \psi _j}=c_{Z\psi _i \psi _j}+c^{*}_{Z\psi _i \psi _j},\nonumber \\ c_{Z\psi _i \psi _j}= & {} \frac{g}{4 \cos \theta _W}\left( U_{i3}U_{j3}^{*}-U_{i2}U_{j2}^{*}\right) , \end{aligned}$$

where the labels *V* and *A* refer to vectorial and axial couplings, respectively. As expected, for Majorana fermions the coupling of the DM with the *Z* is only axial. The couplings with the Higgs boson instead, is given by:

130 $$\begin{aligned} g_{h\psi _i \psi _j}=\frac{1}{\sqrt{2}}\left( y_1 U_{i2}^{*}U_{j1}^{*}+y_2 U_{j3}^{*}U_{i1}^{*}\right) . \end{aligned}$$

For the DM phenomenology, in particular for the DM annihilation, the couplings between the neutral states $\psi _i$, their charged partner and the $W^\pm$-boson are relevant. These can be written as:

131 $$\begin{aligned} g^{V}_{W^\mp \psi ^{\pm }\psi _i}= & {} \frac{g}{2\sqrt{2}}\left( U_{i3}-U_{i2}^{*}\right) , \nonumber \\ g^{A}_{W^\mp \psi ^{\pm }\psi _i}= & {} \frac{g}{2\sqrt{2}}\left( U_{i3}+U_{i2}^{*}\right) . \end{aligned}$$

We will trade in our numerical study the parameters $y_1,y_2$ with $y,\theta$, similar to Ref. [176], so that:

132 $$\begin{aligned} y_1=y\cos \theta ,\quad y_2=y \sin \theta . \end{aligned}$$

These relations allow us to identify our setup as the limiting case of a SUSY theory in which the DM candidate neutralino is a bino-higgsino mixture once these substitutions are considered:

133 $$\begin{aligned}&y\rightarrow \frac{g \tan \theta _W}{\sqrt{2}},\quad M_N \rightarrow M_1,\quad M_L \rightarrow -\mu ,\nonumber \\&\cos \theta \rightarrow -\cos \beta ,\quad \sin \theta \rightarrow \sin \beta , \end{aligned}$$

with $M_1$, $\mu$ and $\tan \beta$ being, respectively, the bino mass parameter, the mass parameter associated with a term bilinear in the up and down-type Higgs superfields and the ratio of the up and down-type Higgs VEVs.

Interestingly, the couplings of the DM with the *Z* and Higgs bosons can manifest “blind spots”, i.e., vanish for specific assignations of the parameters of the theory. The blind spot in the coupling of the DM with the Higgs can be found by rewriting the coupling as [173, 340]:

134 $$\begin{aligned} g_{h\psi _1 \psi _1}=-\frac{y^2 v_h \left( m_{\psi _1} +M_L \sin 2\theta \right) }{M_L^2 + 2 M_L m_{\psi _1}-3 m_{\psi _1}^2 +y^2 v_h^2/2}, \end{aligned}$$

which, as evident, vanishes when:

135 $$\begin{aligned} M_L\,\sin 2\theta +m_{\psi _1 }=0. \end{aligned}$$

Given the chosen sign convention, the blind spot appears for $\sin 2\theta <0$. A blind spot in the coupling of the DM with the *Z* boson is, instead, achieved, when $|U_{12}|^2=|U_{13}|^2$ which corresponds to:

136 $$\begin{aligned} \tan \theta =\pm 1,\quad \text{ and/or }\quad M_L=m_{\psi _1}. \end{aligned}$$

The presence of these blind spots is particularly relevant for direct DM detection. Indeed, the two components, SI and SD, of the DM scattering cross-section on nuclei are sensitive to the couplings of the DM with Higgs boson and the *Z* boson, respectively. The corresponding cross-sections are given by:

137 $$\begin{aligned} \sigma _{\psi _1 p}^\mathrm{SI}=\frac{\mu _{\psi _1 p}^2}{\pi m_h^4}|g_{h\psi _1 \psi _1}|^2\frac{m_p^2}{v_h^2} {\left[ f_p \frac{Z}{A}+f_n \left( 1-\frac{Z}{A}\right) \right] }^2, \end{aligned}$$

in the case of SI interactions, while SD cross-section, originated by the (axial) interactions with the *Z* boson, reads:

138 $$\begin{aligned} \sigma _{\psi _1 p}^\mathrm{SD}=\frac{\mu _{\psi _1 p}^2}{\pi m_Z^4}|g_{Z\psi _1 \psi _1}^A|^2 {\left[ A_u^{Z} \varDelta _u^p+ A_d^Z \left( \varDelta _d^p+\varDelta _s^p\right) \right] }^2. \end{aligned}$$

As already discussed in the case of the Singlet-Doublet model with a Dirac fermion DM, the phenomenology related to the DM relic density features many similarities with the SM Z and Higgs portal models studied in Sect. 7. The DM annihilation rates into $W^{+}W^{-}$ and *ZZ*/*Zh* final states are enhanced by t-channel exchange of the charged particle $\psi ^{\pm }$, for the first, and by the additional neutral fermions, for the latter. A further enhancement can be provided by co-annihilation processes. The annihilation cross-sections into *WW* and *ZZ* final states have sizable s-wave contributions, making them sensitive also to indirect signals (this would occur also in the case of a Dirac fermion DM. This possibility has not been explicitly discussed in the previous subsection because of the overwhelming constraints from DD). The annihilation cross-section into SM fermion pairs features also a s-wave contribution which is, however, helicity suppressed. Consequently, the latter is relevant only for the DM masses not very far from the one of the top-quark.

**Fig. 27:**
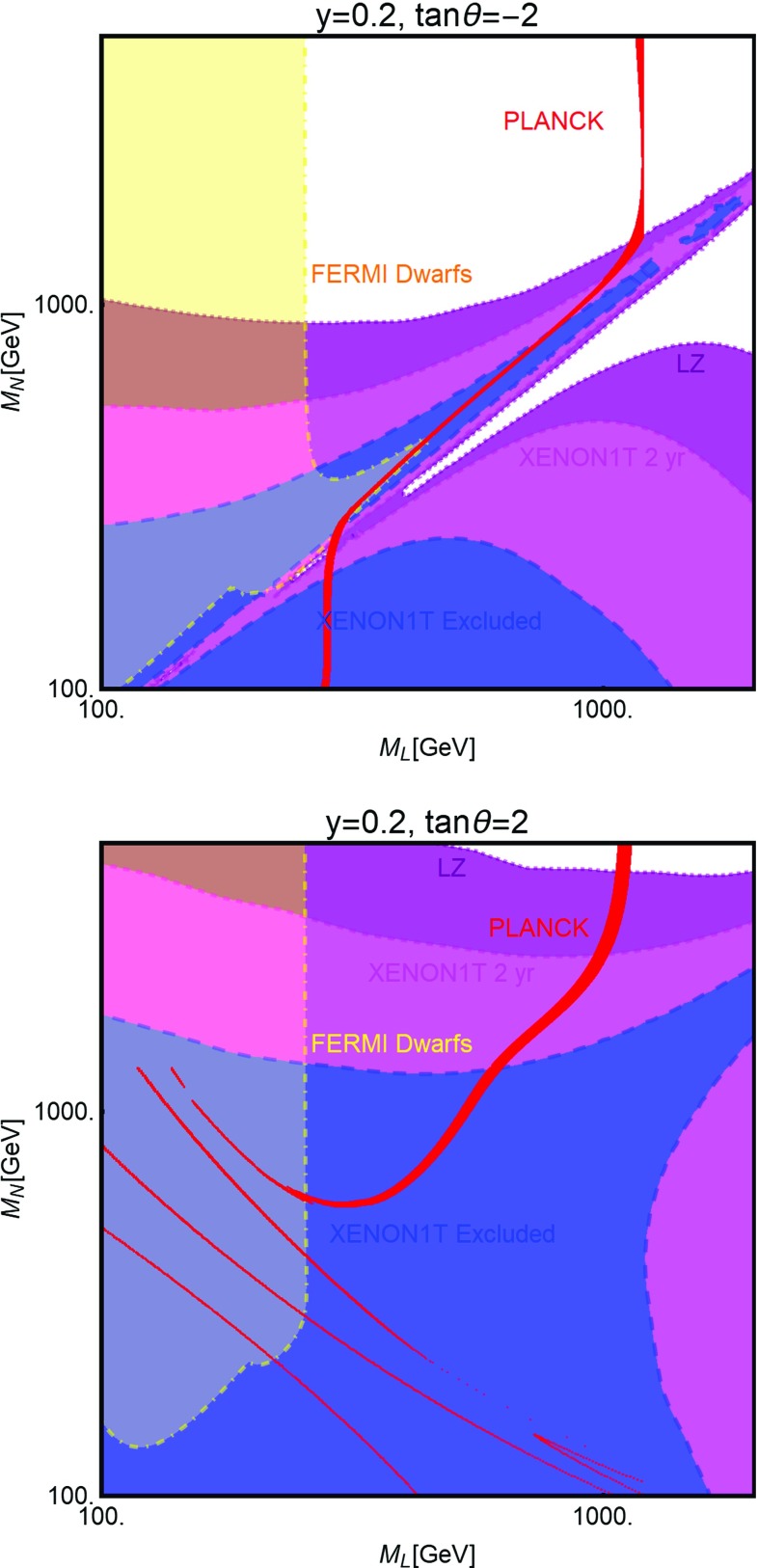
Summary of the constrains for the Singlet-Doublet model with a Majorana fermion DM in the bi-dimensional plane $(M_L,M_N)$ by taking $y=0.2$ and $\tan \theta =-2$ (top panel) $\tan \theta =2$ (bottom panel). The red lines are the iso-contours corresponding to the correct DM relic density. The blue, magenta and purple regions correspond to, respectively, the excluded region by XENON1T and the projected sensitivity regions of XENON1T itself, accounting for 2 years of exposure, and LZ. The yellow regions with yellow dashed-line boundary is excluded by DM ID from Fermi-LAT satellite

A first illustration of the DM constraints, for Singlet-Doublet model with a Majorana fermion DM, is provided in Fig. 27. We have reported there the constraints from the DM relic density and detection strategies in the bi-dimensional plane $(M_L,M_N)$. The coupling *y* has been set to 0.2, i.e., the value corresponding to the SUSY limit, while we have chosen two assignations for $\tan \theta$, namely $\pm 2$. Similar to the case of a Dirac fermion DM the correct DM relic density can be achieved only up to a maximal value of the DM mass, of approximately 1.1 TeV. This corresponds to the case of pure higgsino-like neutralino DM in supersymmetric models with the DM relic density mostly determined by annihilations into *WW* final state controlled by the gauge interactions.^38^ The current limits from DD mostly affect the region $M_L \sim M_N$ which would correspond to maximal mixing between the singlet and the doublet components of the DM and then to maximal couplings with Higgs boson.^39^ The anticipated sensitivities of XENON1T and LZ will, instead, allow to sensibly increase the coverage of the parameter space. Limits from indirect DM detection by Fermi show potential complementarity in the low DM mass region (see also [355]). We notice in particular the different shapes of DD contours according to the different signs of $\tan \theta$; in the case of $\tan \theta =-2$, the presence of the blind spot corresponding to Eq. (135) is evident.

**Fig. 28:**
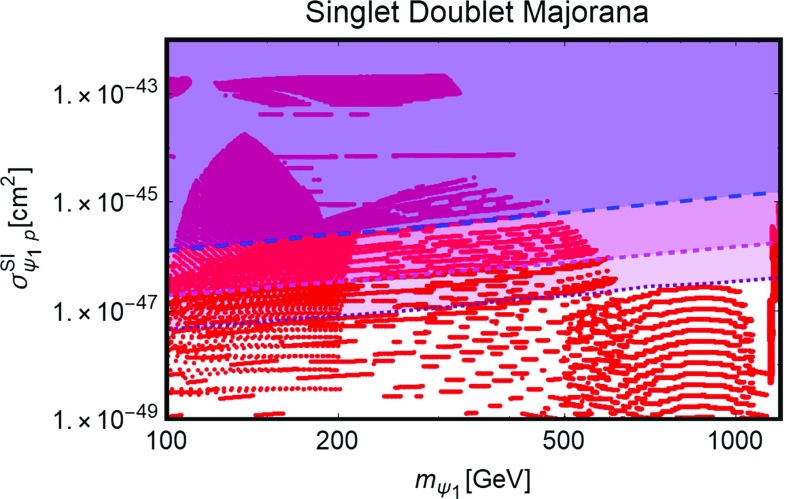
Model points (red) with the correct DM relic density, emerging from a scan on the $M_N$, $M_L$, $y,\,\theta$ parameters (see main text for details), in the bi-dimensional plane $(m_{\psi _1},\sigma _{\psi _1 p}^\mathrm{SI})$. The blue region is currently excluded by DD limits while the magenta and purple regions correspond to the expected future sensitivity reaches of XENON1T and LZ, respectively

Similar to the case of a Dirac fermion DM, we have conducted a more extensive analysis, focused on the relation between the DM relic density and DD, by performing a scan over the four free parameters $M_N$, $M_L$, $y,\,\theta$. The ranges chosen this time, are the following:

139 $$\begin{aligned} M_N,M_L\in & {} \left[ 100,1100\right] \,\text{ GeV },\nonumber \\ y\in & {} [10^{-6},0.2],\nonumber \\ |\tan \theta |\in & {} \left[ 1,20\right] . \end{aligned}$$

The points featuring the correct DM relic density have been reported in Fig. 28 in the bi-dimensional plane $(m_{\psi _1},\sigma _{\psi _1 p}^\mathrm{SI})$. The plot shows also the current and expected limits by the “Xenon based” experiments XENON1T and LZ. Despite of the fact that DD searches can efficiently probe the scenario under consideration, because of the presence of blind spots in the couplings relevant for the DM scattering on nuclei as well as the eventual effect of co-annihilations, a sizable number of points with viable DM relic density would survive even in the absence of signals at LZ.

We conclude by commenting briefly on the possible collider tests of the model under consideration. As already pointed out in the previous subsection, because of the sizable couplings with the SM gauge bosons, the charged fermion $\psi ^{\pm }$ and the neutral fermions $\psi _{2,3}$ can be produced through Drell-Yann processes, followed by successive decay into the DM and a (either on-shell or off-shell) $W^\pm /Z$ boson, respectively, leading to events with 2 - 3 leptons and missing energy. Given the similarity with SUSY setups, one could recast the results from the corresponding searches of chargino/neutralino production [290, 356] in this framework. The present relevant collider constraints are, however, not competitive with the ones from other DM searches [176, 337] and thus, for simplicity, have not been reported in Fig. 27.

## Summary and discussion

We have discussed impact of the current and possible future DD limits, possibly complemented by the ones from ID and/or collider searches, in several simplified realizations of the WIMP DM.

The first and simplest classes of models considered are the ones in which the interactions of a pair of SM singlet scalar, fermionic and vectorial DMs and a pair of SM fermions, are mediated by electrically neutral s-channel (portal) mediators. In the minimal most case the particle spectrum of the SM should be complemented by just a new state, i.e., the DM candidate, since portal interactions can be mediated either by the SM-Higgs or by the Z-boson, although in the last case a theoretically consistent construction is more contrived. In the case of SM-Higgs portal, for all the DM spin assignations, SI interactions with nucleons are induced. The consequent very strong limits, due to the light mediator, are incompatible with the thermal relic density ad exception of DM masses above the TeV scale or the “pole”, i.e., $m_\mathrm{DM} \simeq m_h/2$, region. This last scenario would nevertheless be ruled out in the absence of signals at XENON1T (assuming a 2 years of exposure time) and LZ. In the *Z*-portal scenario current limits on the SI cross-section already exclude the pole region. These strong limits can nevertheless be partially overcome in two setups: (i) a fermionic DM with only axial couplings with the *Z*, as naturally realized in the case of a Majorana fermion DM and, (ii) a vectorial DM coupled through Chern–Simons term. In these two cases the DM features SD interactions with nuclei, whose constraints are sensitively weaker. In particular, in the case of a Majorana fermion DM, the thermal DM with mass of a few hundreds GeV would remain viable even in the absence of signals at the next generation detectors.

The SM-Higgs and Z-portal setups are easily extended to the cases of BSM spin-0 and spin-1 mediators, respectively. In the case of scalar mediators we have imposed, in order to preserve $SU(2)_L$ invariance, a Yukawa structure for the couplings of the mediator with the SM fermions. This, on one side, implies a suppression of the DM annihilation cross-section for masses below the one of the top-quark (unless the *SS* final state is kinematically accessible). At the same time, possible collider signals are also strongly suppressed so that the corresponding limits are not competitive with respect to the ones from DD and have been neglected for simplicity. Despite of the different velocity dependencies of the annihilation cross-sections, the regions of the correct DM relic density are then mostly determined by Yukawa structure of the couplings between the mediator and the SM fermions. The correct DM relic density is indeed obtained, far from the resonance regions, only when the $\overline{t} t$ and/or *SS* annihilation channels are kinematically open. Regarding DD, the limits are associated to SI component of the DM scattering cross-section for all the different assignations of the DM spin. The shape of the DD iso-contours are, however, different for the various DM scenarios. This is due to the different assignations of the couplings with the dimension of mass for the scalar and vectorial DM. Theoretical considerations suggest, indeed, to parametrized these couplings in terms of a fundamental mass scale, the mass of the mediator and the DM mass in the cases of scalar and vectorial DM, respectively, and an unknown dimensionless coupling. The current limits still allow masses of a few hundreds GeV for both the DM and the mediator while XENON1T, in the absence of signals after 2 years of exposure, will exclude mediator masses up to approximately 1 TeV and DM masses up to a few TeV. Given the several free parameters, for clarity of the picture, we have focused our investigation on the masses of the new particle states and fixed the couplings to be close to $\mathscr {O}\,(1)$ (see for alternative, e.g., Ref. [357]). We notice on the other hand that lowering the couplings would simultaneously suppress both the DD rate and the DM annihilation cross-section, in particular the *SS* channel becomes negligible as soon as the DM couplings $\lambda ^S_\chi$, $g_\psi$, $\eta ^S_V$ deviate sensitively from $\mathscr {O}\,(1)$ values. As a consequence, in this setup, the thermal DM is achieved only in the pole region which requires particular fine tuning because of the typical small decay width of the scalar mediator.

The scenario of spin-1 BSM s-channel mediator is even more constrained than the spin-0 case. Indeed, the constraints from SI cross-section are typically much stronger, because of an effective enhancement of the cross-section due to the isospin violating interactions of the $Z'$ with nucleons, so that masses of the DM and the mediator approximately below 5 TeV are already excluded. In the case of no signals at the next generation DD experiments, the exclusion regions will extend up to masses of ${\mathscr {O}}\sim 10~{\mathrm{TeV}}$, beyond the reach of LHC. In addition, the (reasonable) assumption of a $Z'$ coupled with both the SM quarks and leptons implies a strong complementarity with the LHC searches of dilepton resonances. The corresponding limits, exclude, for the models considered here, masses of the $Z'$ between 2 and 3 TeV (the exclusion can be even above 4 TeV in other realizations [358]), even in setups in which the SI component of the DD cross-section is suppressed or absent. We remark again that although in our analysis we have limited to some fixed assignations of the couplings, our results, nevertheless, have general validity because of the strong correlation between the DM relic density and its scattering rate of nucleons. For example, reducing the sizes of the couplings would actually reduce the viable parameter regions since the correct DM relic density would then be achieved only in correspondence of the s-channel resonances.

Despite of the fact that our work is focused on scenarios already probed by the current and will be tested by the near future DD experiments, we have nevertheless also discussed a setup in which DD is, in general, evaded: the pseudoscalar portal. Under the assumption of CP conservation only a fermionic DM is considered in this case. Most of the parameter space is substantially insensitive to DD (we remind here that we have, conservatively, considered values of the pseudoscalar mass above 1 GeV in order to avoid flavour constraints) since tree level interactions with nucleons are momentum suppressed and, furthermore, are not subject to coherent enhancement. A rather limited region of the parameter space might still be probed by 1- and multi-TON detectors because of an one-loop induced SI cross-section. The thermal DM is nevertheless sensitively constrained from ID. In addition, there is again a strong complementarity from the collider constraints, dominating for this scenario from monojet searches. A light pseudoscalar mediator can be interpreted as the pseudo-Goldstone boson of a spontaneously broken global *U*(1) symmetry. We have then considered the case of a complex scalar mediator which can be decomposed into a scalar and a light pseudoscalar components. Although in this case sizable DD limits are reintroduced, the thermal DM still remains viable in the large portions of the parameter space due to the presence of efficient annihilation processes in the *aa* and *Sa* final states.

Relaxing the hypothesis of a SM singlet BSM mediator and assigning it non-trivial quantum numbers under the SM gauge groups, one can construct a simple and predictive class of model which we have labeled as t-channel portal. In this case a single DM state is coupled with the mediator and a SM fermion, according to the gauge charge assignation of the mediator (for simplicity we have restricted most of our analysis to couplings with the right-handed up-type quarks). Contrary to the other scenarios considered in this work, here the DM pair annihilation occurs through t-channel exchange of the mediator while DD scattering is induced by its s-channel exchange. Focusing, for simplicity, mostly to the case in which the mediator field has the same quantum numbers with respect to the SM gauge group as the right-handed up-type quarks, the scenarios, i.e., a complex scalar and a Dirac fermion DM, in which SI interactions are present are excluded for $\mathscr {O} (1)$ values of the couplings and for mediator masses up to $\mathscr {O}\sim 10$ TeV. On the contrary, thermal Majorana fermion DM is still viable for masses below a TeV and will be extensively probed by the next generation of DD experiments.

We have then performed some steps towards more theoretically motivated realizations of dark portals. As well known, the bilinears $H^{\dagger } H$ and $B^{\mu \nu }$ (together with a new BSM field strength) are Lorentz and gauge invariant, so naturally lead to portal interactions with a dark sector, even if this is completely secluded. We have thus, considered the cases of (i) a scalar mediator coupled both to the DM and the SM-Higgs boson and mixes with the latter because of a non-zero VEV and, (ii) a $Z'$ coupled to the *Z* boson through a kinetic mixing term, also responsible for a mixing between the two spin-1 states. These two mixing in turn allow the BSM states to interact with the other SM states. Also concerning the coupling of the DM with the mediators we have considered less generic assignations with respect to the cases considered in the previous frameworks. In the case of SM-Higgs $+$ spin-0 portal we have explicitly considered a dynamical origin for the DM mass. Indeed, a fermionic DM has been assumed to have Yukawa interaction so that its mass is originated by the VEV of the new scalar field. Similarly, a vectorial DM has been assumed to be the vector boson of a spontaneously broken dark *U*(1) gauge symmetry and its mass is again related to the VEV of the new scalar field, also charged with respect to the same *U*(1) group.

As the last case of study we have considered models in which the DM features SM gauge interactions, either being the lightest neutral component of a $SU(2)_L$ multiplet (in our studied examples we have focused on the case of $SU(2)_L$ doublets) or a mixing between the latter and a SM singlet. These models are characterized by the fact that the DM sector is composed of multiple states. Particularly relevant is the case in which some of these states are very close in mass to the DM. In this setup the DM annihilation cross-section features a twofold enhancement with respect to the other Dark portals. First of all the annihilation into $W^{+}W^{-}$ is enhanced by the t-channel exchange of electroweakly charged states belonging to the DM sector (this, at the same time, requires that the DM has a non-negligible *SU*(2) component). A further enhancement comes from co-annihilation effects. Despite of a limited number of free parameters, these models are capable of encompassing many theoretically motivated scenarios like for example SUSY models. Interestingly, the Singlet-Doublet models considered at the end of Sect. 12 allows renormalizable couplings between the DM with the SM Higgs and the Z bosons. From the phenomenological point of view, the Singlet-Doublet model with a Dirac fermion DM features the greatest similarities with the SM Z-portal model, sharing with the latter the extremely strong constraints from DD. This model appears viable only in a very fine tuned co-annihilation configuration. On the contrary, only the response of the future experimental facilities can fully probe the case of a Majorana fermion DM. More particular is the case of the Inert Doublet Model. Its main limitation is represented by the very efficient annihilation cross-section into gauge bosons. The viable DM relic density can be obtained when this annihilation channel is kinematically allowed and only very specific assignations of the masses of the new particle sector are considered.

## Conclusions

We have reviewed the theoretical foundations of the WIMP paradigm and discussed the limits, prospect and challenges of direct DM detection in a multitude of models encompassing scalar, vectorial and fermionic DM setups. In the light of extensive programme of direct DM searches and including a broad variety of complementary probes from indirect and collider searches, we assessed the status of the WIMP paradigm in the context of simplified models, accounting for the current and projected limits.

In particular, we have reviewed well known portals such as the SM-Higgs portal and the Z-portal. We have also addressed the popular dark $Z^{\prime }$ portal and many others models that possess in their spectrum more than one mediator. Moreover, we have also investigated new models dictated by the kinetic mixing, often used in dark photon models.

We concluded that the simplest constructions, i.e., the SM dark portals will be substantially ruled out, ad exception of the case of a fermionic DM with only axial couplings with the Z-boson (e.g., a Majorana fermion DM), in the absence of signals in the next generation of DD experiments.

The most straightforward extension of the SM dark portals, represented by the introduction of BSM s-channel mediators, are, similarly, strongly constrained in the presence of SI interactions of the DM with the nuclei. In particular, the case of spin-1 mediator is strongly disfavored because of the presence of complementary constraints from searches of resonances at the LHC, pushing the DM mass towards the multi-TeV scale.

The tension with DD constraints can be relaxed somehow in the next-to-minimal scenarios, featuring multiple mediators or new states lighter than the DM (we have reviewed the example of a light pseudoscalar).

In summary, we combined a plethora of experimental data set and theoretical models, computed the DM relic density, direct, indirect and collider observables to have a clear picture of where the WIMP paradigm stands and the future prospects. It is clear that most of the WIMP models will be scrutinized in the next decades, highlighting the paramount role of the next generation of experiments.

Moreover, our work shows that some DM constructions will survive the null results from the collider, direct and indirect experiments,suggesting that a further step in sensitivity reach is needed to falsify the WIMP paradigm.

## Appendix A: Annihilation cross-sections

The thermally averaged pair annihilation cross-section is defined as:

A.1 $$\begin{aligned} \langle \sigma v \rangle= & {} \frac{{K_2\left( \frac{m_\chi }{T}\right) }^{-2}}{8 m_\chi ^4 T }\int _{4 m_\chi ^2}^{\infty } ds~ \sigma (s) \nonumber \\&\times \, \sqrt{s} \left( s-4 m_\chi ^2\right) K_1\left( \frac{\sqrt{s}}{T}\right) . \end{aligned}$$

Away from resonances, a manageable analytical expression for the thermally averaged pair annihilation cross-section is obtained by performing the formal velocity expansion, in the non-relativistic limit, as defined in Ref. [23]. The thermally averaged cross-section can be computed as:

A.2 $$\begin{aligned} \langle \sigma v \rangle = \frac{2 x^{3/2}}{\pi ^{1/2}}\int _0^{\infty } \sigma v_\mathrm{lab} \epsilon ^{1/2}e^{-\epsilon x}, \end{aligned}$$

where:

A.3 $$\begin{aligned} v_\mathrm{lab}=\frac{2 \epsilon ^{1/2}{\left( 1+\epsilon \right) }^{1/2}}{\left( 1+2\epsilon \right) }, \quad \epsilon =\frac{s-4 m_\chi ^2}{4 m_\chi ^2},\quad x=\frac{m_\chi }{T}. \end{aligned}$$

This kind of integral can be computed analytically by considering an expansion in the series of $\epsilon$ for $\sigma v_\mathrm{lab}$, namely:

A.4 $$\begin{aligned} \sigma v_\mathrm{lab}=a_0 +a_1 \epsilon +a_2 \epsilon ^2 + \cdots . \end{aligned}$$

We will report subsequently the full analytical expressions for the thermally averaged cross-section in the various dark portal scenarios investigated in this work, using the velocity expansion. Apart from some exceptions, we will adopt general notations for the couplings so that the various expression can be applied to the different models discussed in the main text as well as being of more general utility. In other words, we will use the generic notations $g_{xxm}$, $g_{y y m}$, $g_{xxyy}$ for the relevant ones involving a generic DM particle “*x*”, the mediator “*m*” and the SM particle “*y*”, respectively (notice that couplings $g_{\chi \chi S},\,g_{\chi \chi S S}$ in these notations should not be confused with the ones defined in Eq. (90) with similar notations, for the case of SM-Higgs + spin-0 portal).

Further, note that we have used the symbols $g_{ZZh},\,g_{WWZ}$ to parametrized the SM $ZZh,\,W^+W^-Z$ couplings, consistent with the aforesaid notations. We will adopt a slightly different notation for the couplings of a scalar (pseudoscalar) mediator *S* (*a*) with the SM fermions, which will be indicated as $g_{Sf}$($g_{af}$) (in this work we have considered the case $g_{S(a) f}=c_{S(a)}\frac{m_f}{v_h}$), and the couplings of the $Z'$ mediator with fermions (both the DM and SM), which will be indicated as $g_{Ii}^{Z'},\,I=V,A,\,i=\psi ,f$ (contrary to what discussed in the main text, these couplings encode the gauge couplings in their definition). Notice that the couplings of scalar mediators with the scalar and vector fields are dimensional, rather than being decomposed, as in the main text, into a physical energy scale and a dimensionless parameter. In the case of spin-0 and spin-1 portals, the expressions for *hh*, *ZZ* and *Zh* final states can be derived directly, by suitable substitutions, from the ones corresponding to the *SS*, $Z'Z'$ and $Z'h$ final states, respectively. The SM-Higgs + spin-0 portal and the kinetic mixing models feature, for the DM, also annihilation processes into *hS* and $ZZ'$ final states, respectively. We won’t explicitly report the corresponding expressions since they are particularly complicated. For the same reason we also do not report analytical expressions for the relevant annihilation cross-sections of the models discussed in Sect. 12.

Finally, we remind that the velocity expansion is not valid in the vicinity of s-channel resonances and kinematic threshold of the annihilation channels [23, 25].

### Appendix A.1: Scalar portal

#### Appendix A.1.1: Scalar dark matter

$$\chi ^{*} \chi \rightarrow \overline{f} f$$:

A.5 $$\begin{aligned} \langle \sigma v \rangle _{ff} = \sum _f n^c_f \frac{(g_{\chi \chi S})^2 (g_{Sf})^2 \left( m_{\chi }^2-m_f^2\right) {}^{3/2}}{8 \pi m_{\chi }^3 \left( m_S^2-4 m_{\chi }^2\right) ^2}. \end{aligned}$$

$$\chi ^{*} \chi \rightarrow W^+ W^-$$:

A.6 $$\begin{aligned} \langle \sigma v \rangle _{WW}= & {} \frac{(g_{\chi \chi S})^2 (g_{WWS})^2 \sqrt{m_{\chi }^2-m_W^2} }{64 \pi m_{\chi }^3 m_W^4 \left( m_S^2-4 m_{\chi }^2\right) ^2}\nonumber \\&\times \left( -4 m_{\chi }^2 m_W^2+4 m_{\chi }^4+3 m_W^4\right) . \end{aligned}$$

$$\chi ^{*} \chi \rightarrow ZZ$$:

A.7 $$\begin{aligned} \langle \sigma v \rangle _{ZZ}= & {} \frac{(g_{\chi \chi S})^2 (g_{Z Z S})^2 \sqrt{m_{\chi }^2-m_Z^2} }{128 \pi m_{\chi }^3 m_Z^4 \left( m_S^2-4 m_{\chi }^2\right) ^2}\nonumber \\&\times \left( -4 m_{\chi }^2 m_Z^2+4 m_{\chi }^4+3 m_Z^4\right) . \end{aligned}$$

$$\chi ^{*} \chi \rightarrow SS$$:

A.8 $$\begin{aligned}&\langle \sigma v \rangle _{SS} = \frac{1}{64 \pi m_\chi ^2} \sqrt{1-\frac{m_S^2}{m_\chi ^2}} \nonumber \\&\quad \times \left( 2 (g_{\chi \chi SS})^2 -\frac{2 g_{\chi \chi S}\, g_{SSS}\, g_{\chi \chi SS}}{m_S^2-4 m_\chi ^2}+\frac{(g_{\chi \chi S})^2 (g_{SSS})^2}{{\left( m_S^2-4 m_\chi ^2\right) }^2} \right. \nonumber \\&\quad \left. +\,\frac{4(g_{\chi \chi S})^4}{{\left( m_S^2-2 m_\chi ^2\right) }^2} -\frac{4 g_{\chi \chi S}\, g_{SSS}\, g_{\chi \chi SS}}{\left( m_S^2-4 m_\chi ^2\right) \left( m_S^2-2 m_\chi ^2\right) }\right. \nonumber \\&\quad \left. +\,\frac{4 g_{\chi \chi S S} (g_{\chi \chi S})^2}{m_S^2-2 m_\chi ^2}\right) . \end{aligned}$$

#### Appendix A.1.2: Fermionic dark matter

$$\overline{\psi }\psi \rightarrow \overline{f} f$$:

A.9 $$\begin{aligned} \langle \sigma v \rangle _{ff} = (g_{\psi \psi S})^2 \sum _f n_c^f \frac{(g_{Sf})^2 \left( m_{\psi }^2-m_f^2\right) {}^{3/2}}{4 \pi m_{\psi } \left( m_S^2-4 m_{\psi }^2\right) ^2}v^2. \end{aligned}$$

$$\overline{\psi }\psi \rightarrow W^+ W^-$$:

A.10 $$\begin{aligned} \langle \sigma v \rangle _{WW}= & {} (g_{\psi \psi S})^2 (g_{SWW})^2 \frac{\sqrt{m_{\psi }^2-m_W^2} }{64 \pi m_{\psi } m_W^4 \left( m_S^2-4 m_{\psi }^2\right) ^2}v^2 \nonumber \\&\times \left( -4 m_{\psi }^2 m_W^2+4 m_{\psi }^4+3 m_W^4\right) . \end{aligned}$$

$$\overline{\psi }\psi \rightarrow ZZ$$:

A.11 $$\begin{aligned} \langle \sigma v \rangle _{ZZ}= & {} (g_{\psi \psi S})^2 (g_{SZZ})^2 \frac{\sqrt{m_{\psi }^2-m_Z^2}}{128 \pi m_{\psi } m_Z^4 \left( m_S^2-4 m_{\psi }^2\right) ^2}v^2 \nonumber \\&\times \left( -4 m_{\psi }^2 m_Z^2 +4 m_{\psi }^4+3 m_Z^4\right) . \end{aligned}$$

$$\overline{\psi }\psi \rightarrow SS$$:

A.12 $$\begin{aligned} \langle \sigma v \rangle _{SS}= & {} \frac{v^2}{192 \pi m_\psi ^2} \sqrt{1-\frac{m_S^2}{m_\chi ^2}} \nonumber \\&\times \left( \frac{8\, g_{SSS} (g_{\psi \psi S})^{3} m_\psi ^3 (2 m_S^2-5 m_\psi ^2)}{(m_S^2-4 m_\psi ^2) (m_S^2-2 m_\psi ^2)^2}\right. \nonumber \\&\left. +\, \frac{3 (g_{SSS})^2 (g_{\psi \psi S})^2 m_\psi ^2}{(m_S^2-4 m_\psi ^2)^2} \right. \nonumber \\&\left. +\,\frac{16 (g_{\psi \psi S})^4 \left( 9 m_\psi ^8 -8 m_\psi ^6 m_S^2+2 m_S^8\right) }{(m_S^2-2 m_\psi ^2)^4}\right) .\nonumber \\ \end{aligned}$$

#### Appendix A.1.3: Vectorial dark matter

$$VV \rightarrow \overline{f} f$$:

A.13 $$\begin{aligned}&\langle \sigma v \rangle _{ff}=\sum _f n_c^f (g_{VVS})^2 (g_{Sf})^2 \nonumber \\&\quad \times \left( \frac{\sqrt{4-\frac{4 m_f^2}{m_V^2}} \left( 4 m_V^2-4 m_f^2\right) }{96 \pi m_V^2 \left( 4 m_V^2-m_S^2\right) ^2}\right. \nonumber \\&\quad \left. +\,\frac{v^2 \left( m_f^2 \left( 7 m_S^2-76 m_V^2\right) +2 m_V^2 \left( m_S^2+20 m_V^2\right) \right) }{144 \pi m_V^2 \left( m_S^2-4 m_V^2\right) ^3 {(1-\frac{m_f^2}{m_V^2})}^{-{1/2}}}\right) .\nonumber \\ \end{aligned}$$

$$VV \rightarrow W^+ W^-$$:

A.14 $$\begin{aligned}&\langle \sigma v \rangle _{WW} = {(g_{WWS})}^2 {(g_{VVS})}^2 \nonumber \\&\quad \times \left( \frac{ \sqrt{4- \frac{4 m_W^2}{m_V^2}} \left( 16 m_V^4-16 m_V^2 m_W^2+12 m_W^4\right) }{768 \pi m_V^2 m_W^4 \left( 4 m_V^2-m_S^2\right) ^2}\right. \nonumber \\&\quad \left. -\, v^2 \left[ \frac{3 m_W^6 \left( 76 m_V^2-7 m_S^2\right) +16 m_V^2 m_W^4 \left( m_S^2-25 m_V^2\right) }{1152 \pi m_V^4 m_W^4\left( m_S^2-4 m_V^2\right) ^3 \sqrt{1-\frac{m_W^2}{m_V^2}}}\right. \right. \nonumber \\&\quad \left. \left. - \,\frac{32 m_V^2 \left( m_S^2+2 m_V^2\right) -4 m_W^2 \left( 7 m_S^2 +68 m_V^2\right) }{1152 \pi m_W^4\left( m_S^2-4 m_V^2\right) ^3 \sqrt{1-\frac{m_W^2}{m_V^2}}} \right] \right) .\nonumber \\ \end{aligned}$$

$$VV \rightarrow ZZ$$:

A.15 $$\begin{aligned}&\langle \sigma v \rangle _{ZZ} = {(g_{VVS})}^2 {(g_{ZZS})}^2\nonumber \\&\quad \times \left( \frac{\sqrt{4 -\frac{4 m_Z^2}{m_V^2}} \left( 16 m_V^4-16 m_V^2 m_Z^2+12 m_Z^4\right) }{1536 \pi m_V^2 m_Z^4 \left( 4 m_V^2-m_S^2\right) ^2}\right. \nonumber \\&\quad \left. -\,v^2 \left[ \frac{3 m_Z^6 \left( 76 m_V^2-7 m_S^2\right) +16 m_V^2 m_Z^4 \left( m_S^2-25 m_V^2\right) }{2304 \pi m_V^4 m_Z^4 \left( m_S^2-4 m_V^2\right) ^3 \sqrt{1-\frac{m_Z^2}{m_V^2}}} \right. \right. \nonumber \\&\quad \left. \left. -\,\frac{32 m_V^2 \left( m_S^2+2 m_V^2\right) -4 m_Z^2 \left( 7 m_S^2+68 m_V^2\right) }{2304 \pi m_Z^4 \left( m_S^2-4 m_V^2\right) ^3 \sqrt{1-\frac{m_Z^2}{m_V^2}}}\right] \right) .\nonumber \\ \end{aligned}$$

$$VV \rightarrow SS$$:

A.16 $$\begin{aligned}&\langle \sigma v \rangle _{SS}=\frac{\sqrt{1-\frac{m_S^2}{m_V^2}}}{288 \pi m_V^2} \left[ \frac{3 (g_{SSS})^2 (g_{VVS})^2}{\left( m_S^2-4 m_V^2\right) ^2}+\frac{4 g_{SSS} (g_{VVS})^3}{m_S^2 m_V^2-2 m_V^4} \right. \nonumber \\&\quad \left. -\,\frac{3 g_{SSS}\, g_{VVS}\, g_{VVSS}}{m_S^2-4 m_V^2} +4 (g_{VVS})^4 \left( \frac{2}{\left( m_S^2-2 m_V^2\right) ^2}+\frac{1}{m_V^4}\right) \right. \nonumber \\&\quad \left. -\,\frac{(2 g_{VVS})^2 g_{VVSS} \left( m_S^2-4 m_V^2\right) }{m_V^2 \left( m_S^2-2 m_V^2\right) }+\frac{3 (g_{VVSS})^2}{4}\right] \nonumber \\&\quad +\,\frac{v^2 \left( m_S^2-4 m_V^2\right) ^{-3} m_V^{-7} \pi ^{-1} }{27648 \left( m_S^2-2 m_V^2\right) ^4 \sqrt{m_V^2-m_S^2}} \left\{ 4 (g_{VVS})^2 m_V^2 \left( 2 m_V^2 - m_S^2\right) \right. \nonumber \\&\quad \left. \times \left( 2 g_{VVSS} \left( m_S^2-4 m_V^2\right) ^3 \left( 21 m_S^8-192 m_S^6 m_V^2+602 m_S^4 m_V^4 \right. \right. \right. \nonumber \\&\quad \left. \left. \left. -\,784 m_S^2 m_V^6+344 m_V^8\right) \right. \right. \nonumber \\&\quad \left. \left. -\,3 (g_{SSS})^2 m_V^2 \left( m_S^2-2 m_V^2\right) ^3 \left( 7 m_S^4-80 m_S^2 m_V^2+64 m_V^4\right) \right) \right. \nonumber \\&\quad \left. +\,16 g_{SSS} (g_{VVS})^3 m_V^2 \left( m_S^2-4 m_V^2\right) ^2 \left( m_S^2-2 m_V^2\right) \right. \nonumber \\&\quad \left. \times \left( 21 m_S^8-216 m_S^6 m_V^2+722 m_S^4 m_V^4-976 m_S^2 m_V^6+440 m_V^8\right) \right. \nonumber \\&\quad \left. -\,12 g_{SSS} \,g_{VVS}\, g_{VVSS} m_V^4 (m^2_S-4 m^2_V) \left( m_S^2-2 m_V^2\right) ^4 \right. \nonumber \\&\quad \left. \times \left( 7 m_S^4-56 m_S^2 m_V^2+40 m_V^4\right) \right. \nonumber \\&\quad \left. +\,16 (g_{VVS})^4 \left( m_S^2-4 m_V^2\right) ^3 \left( 19 m_S^{10}-180 m_S^8 m_V^2+706 m_S^6 m_V^4 \right. \right. \nonumber \\&\quad \left. \left. -\,1408 m_S^4 m_V^6 -544 m_V^{10} +1416 m_S^2 m_V^8\right) +3 g_{VVSS}^2 m_V^4 \right. \nonumber \\&\quad \left. \times \left( m_S^2-4 m_V^2\right) ^3 \left( 7 m_S^2-4 m_V^2\right) \left( m_S^2-2 m_V^2\right) ^4\right\} . \end{aligned}$$

### Appendix A.2: Spin-1 portal

#### Appendix A.2.1: Scalar dark matter

$$\chi \chi ^{*} \rightarrow \overline{f} f$$:

A.17 $$\begin{aligned} \langle \sigma v \rangle _{ff}= & {} (g_{\chi \chi Z})^2 v^2 \sum _{f} n_c^f \frac{\sqrt{m_\chi ^2-m_f^2}}{3 \pi m_\chi \left( m_{Z'}^2-4 m_\chi ^2\right) ^2} \nonumber \\&\times \left( 2{(g_{Af}^{Z'})}^2 \left( m_{\chi }^2- m_f^2\right) + {(g_{Vf}^{Z'})}^2 (2 m_\chi ^2+ m_f^2)\right) .\nonumber \\ \end{aligned}$$

$$\chi \chi ^{*} \rightarrow W^+ W^-$$:

A.18 $$\begin{aligned} \langle \sigma v \rangle _{WW}=\frac{ (g_{\chi \chi Z})^2 (g_{WWZ})^2 v^2 \left( 3 m_W^4+20 m_W^2 m_\chi ^2 +4 m_\chi ^4\right) }{6 \pi m_\chi ^{-2} m_W^4 \left( m_Z^2-4 m_\chi ^2\right) ^2 {\left( 1-\frac{m_W^2}{m_\chi ^2}\right) }^{-{3/2}}}.\nonumber \\ \end{aligned}$$

$$\chi \chi ^{*} \rightarrow Z^\prime Z^\prime $$:

A.19 $$\begin{aligned}&\langle \sigma v \rangle _{Z'Z'}=\frac{\sqrt{m_\chi ^2-m_{Z'}^2}}{16 \pi m_{Z'}^4 m_\chi ^3 \left( m_{Z'}^2-2 m_\chi ^2\right) ^2} \nonumber \\&\quad \times \left( 8 (g_{\chi \chi Z'})^2 g_{\chi \chi Z'Z'} m_\chi ^2 (m_{Z'}^2-m^2_\chi ) \left( m_{Z'}^2-2 m_\chi ^2\right) ^2\right. \nonumber \\&\quad \left. +\,16 (g_{\chi \chi Z'})^4 m_\chi ^4 \left( m_{Z'}^2-m_\chi ^2\right) ^2 + {(g_{\chi \chi Z'Z'})}^2 \right. \nonumber \\&\quad \left. \times \left( m_{Z'}^2-2 m_\chi ^2\right) ^2 \left( 3 m_{Z'}^4-4 m_{Z'}^2 m_\chi ^2+4 m_\chi ^4\right) \right) \nonumber \\&\quad +\,\frac{v^2 \left( m_{Z'}^2-2 m_\chi ^2\right) ^{-4}}{192 \pi m_{Z'}^2 m_\chi ^3 \sqrt{m_\chi ^2-m_{Z'}^2}} \left( 16 (g_{\chi \chi Z'})^4 m_\chi ^4 \right. \nonumber \\&\quad \left. \times \left( m_{Z'}^2 -m_\chi ^2\right) ^2 \left( 3 m_{Z'}^4-20 m_{Z'}^2 m_\chi ^2+36 m_\chi ^4\right) \right. \nonumber \\&\quad \left. +\,8 (g_{\chi \chi Z'})^2 g_{\chi \chi Z'Z'} m_\chi ^2 (m_{Z'}^2-m^2_\chi ) \left( m_{Z'}^2-2 m_\chi ^2\right) ^2 \right. \nonumber \\&\quad \left. \times \left( 5 m_{Z'}^4-26 m_{Z'}^2 m_\chi ^2+36 m_\chi ^4\right) +9 {(g_{\chi \chi Z'Z'})}^2 \right. \nonumber \\&\quad \left. \times \left( m_{Z'}^2-2 m_\chi ^2\right) ^4 \left( 5 m_{Z'}^4-8 m_{Z'}^2 m_\chi ^2+4 m_\chi ^4\right) \right) . \end{aligned}$$

$$\chi \chi ^{*} \rightarrow Zh$$:

A.20 $$\begin{aligned} \langle \sigma v \rangle _{Zh}= & {} (g_{\chi \chi Z})^2 (g_{ZZh})^2 v^2 \nonumber \\&\times \, \sqrt{1-\frac{(m_h-m_Z)^2}{4 m_\chi ^2}}\sqrt{1-\frac{(m_h+m_Z)^2}{4 m_\chi ^2}}\nonumber \\&\times \, \frac{(m_h^2-m_Z^2)^2-8 (m_h^2-5 m_Z^2) m_\chi ^2+16 m_\chi ^4}{384 \pi m_Z^2 m_\chi ^2 \left( m_Z^2-4 m_\chi ^2\right) ^2}.\nonumber \\ \end{aligned}$$

#### Appendix A.2.2: Fermionic dark matter

For simplicity we explicitly report the case of a Dirac fermion DM. The expressions for a Majorana fermion DM can be derived straightforwardly from these.

$$\overline{\psi } \psi \rightarrow \overline{f} f$$:

A.21 $$\begin{aligned} \langle \sigma v \rangle _{ff}= & {} \sum _{f} n_c^f \frac{2\sqrt{m_\psi ^2-m_f^2}}{4 \pi m_\psi m_{Z'}^4 \left( m_{Z'}^2-4 m_\psi ^2\right) ^2} \nonumber \\&\times \left[ {(g_{Af}^{Z'})}^2 {({g_{A\psi }^{Z'}})}^2 m_f^2 \left( m_{Z}^2-4 m_\psi ^2\right) ^2 + 2 m_{Z'}^4 {(g_{V\psi }^{Z'})}^2 \right. \nonumber \\&\left. \times \left( 2 {(g_{Af}^{Z'})}^2 \left( m_\psi ^2-m_f^2\right) +{(g_{Vf}^{Z'})}^2 \left( m_f^2+2 m_\psi ^2\right) \right) \right] \nonumber \\&-\,\frac{v^2 \left( m_{Z'}^2-4 m_\psi ^2\right) ^{-3}}{24 \pi m_\psi m_{Z'}^4 \sqrt{m_\psi ^2-m_f^2}} \nonumber \\&\times \left[ {(g_{Af}^{Z'})}^2 \left\{ 2 m_{Z'}^4 (g_{V\psi }^{Z'})^2 (m^2_f-m^2_\psi ) \right. \right. \nonumber \\&\left. \left. \times \left( -2 m_\psi ^2 \left( 46 m_f^2+m_{Z'}^2\right) +11 m_f^2 m_{Z'}^2+56 m_\psi ^4\right) \right. \right. \nonumber \\&\left. \left. -\,(g_{A\psi }^{Z'})^2 \left( m_{Z'}^2-4 m_\psi ^2\right) \right. \right. \nonumber \\&\left. \left. \times \left( 23 m_f^4 m_{Z'}^4-192 m_f^2 m_\psi ^6-4 m_f^2 m_\psi ^2 m_{Z'}^2 \left( 30 m_f^2+7 m_{Z'}^2\right) \right. \right. \right. \nonumber \\&\left. \left. \left. +\,8 m_\psi ^4 \left( 30 m_f^4+12 m_f^2 m_{Z'}^2+m_{Z'}^4\right) \right) \right\} \right. \nonumber \\&\left. +\,m_{Z'}^4 {(g_{Vf}^{Z'})}^2 \left\{ 4 (g_{\psi A}^{Z'})^2 \left( m_f^4+m_f^2 m_\psi ^2-2 m_\psi ^4\right) \left( m_{Z'}^2-4 m_\psi ^2\right) \right. \right. \nonumber \\&\left. \left. +\, {(g_{V\psi }^{Z'})}^2\left( -11 m_f^4 m_{Z'}^2+4 m_\psi ^4 \left( 14 m_f^2+m_{Z'}^2\right) \right. \right. \right. \nonumber \\&\left. \left. \left. -\,2 m_f^2 m_\psi ^2 \left( m_{Z'}^2-46 m_f^2\right) -112 m_\psi ^6\right) \right\} \right] . \end{aligned}$$

$$\overline{\psi } \psi \rightarrow W^+ W^-$$:

A.22 $$\begin{aligned} \langle \sigma v \rangle _{WW}= & {} (g_{WWZ'})^2 \frac{ {(g_{V\psi }^{Z'})}^2 \sqrt{m_\psi ^2-m_W^2}}{4 \pi m_W^4 m_\psi \left( m_{Z'}^2-4 m_\psi ^2\right) ^2 } \nonumber \\&\times \left( -3 m_W^6-17 m_W^4 m_\psi ^2+16 m_W^2 m_\psi ^4+4 m_\psi ^6\right) \nonumber \\&+\, \frac{v^2 (g_{WWZ'})^2\sqrt{m_\psi ^2-m_{W}^2}}{48 \pi m_W^4 m_\psi \left( m_{Z'}^2-4 m_\psi ^2\right) ^3}\nonumber \\&\times \left[ 4 {(g_{A\psi }^{Z'})}^2 \left( -3 m_W^6-17 m_W^4 m_\psi ^2 \right. \right. \nonumber \\&\left. \left. +\,16 m_W^2 m_\psi ^4+4 m_\psi ^6\right) \left( m_{Z'}^2-4 m_\psi ^2\right) + \right. \nonumber \\&\left. +\, {(g_{V\psi }^{Z'})}^2 \left\{ 33 m_W^6 m_{Z'}^2+8 m_\psi ^6 \left( 58 m_W^2+5 m_{Z'}^2\right) \right. \right. \nonumber \\&\left. \left. +\,4 m_W^2 m_\psi ^4 \left( 19 m_{Z'}^2-298 m_W^2\right) \right. \right. \nonumber \\&\left. \left. +\, 2 m_W^4 m_\psi ^2 \left( 47 m_{Z'}^2-138 m_W^2\right) +32 m_\psi ^8 \right\} \right] .\nonumber \\ \end{aligned}$$

$$\overline{\psi } \psi \rightarrow Z' Z'$$:

A.23 $$\begin{aligned} \langle \sigma v \rangle _{Z'Z'}= & {} \frac{\left( {(g_{A\psi }^{Z'})}^4 m_{Z'}^2+m_{Z'}^2 {(g_{V\psi }^{Z'})}^4\right) }{\pi m_\psi \left( m_{Z'}^3-2 m_\psi ^2 m_{Z'}\right) ^2 \left( m_\psi ^2-m_{Z'}^2\right) ^{-{3/2}} }\nonumber \\&+\, \frac{\left( 2 {(g_{A\psi }^{Z'})}^2 {(g_{V\psi }^{Z'})}^2 \left( 4 m_\psi ^2-3 m_{Z'}^2\right) \right) }{\pi m_\psi \left( m_{Z'}^3-2 m_\psi ^2 m_{Z'}\right) ^2 \left( m_\psi ^2-m_{Z'}^2\right) ^{-{3/2}} }\nonumber \\&+\, \frac{v^2\sqrt{m_\psi ^2-m_{Z'}^2}}{4 \pi m_\psi \left( m_{Z'}^3-2 m_\psi ^2 m_{Z'}\right) ^4} \nonumber \\&\times \left[ {(g_{A\psi }^{Z'})}^4 \left( 128 m_\psi ^{10}+23 m_{Z'}^{10}-118 m_\psi ^2 m_{Z'}^8 \right. \right. \nonumber \\&\left. \left. +\,172 m_\psi ^4 m_{Z'}^6+32 m_\psi ^6 m_{Z'}^4-192 m_\psi ^8 m_{Z'}^2\right) \right. \nonumber \\&\left. -\,2 m_{Z'}^2 {(g_{A\psi }^{Z'})}^2 {(g_{V\psi }^{Z'})}^2 \left( 160 m_\psi ^8+21 m_{Z'}^8 \right. \right. \nonumber \\&\left. \left. -\,182 m_\psi ^2 m_{Z'}^6 +508 m_\psi ^4 m_{Z'}^4-528 m_\psi ^6 m_{Z'}^2\right) \right. \nonumber \\&\left. +\,m_{Z'}^6 (g_{V\psi }^{Z'})^4 \left( 76 m_\psi ^4+23 m_{Z'}^4-66 m_\psi ^2 m_{Z'}^2\right) \right] .\nonumber \\ \end{aligned}$$

$$\overline{\psi } \psi \rightarrow Zh$$:

A.24 $$\begin{aligned}&\langle \sigma v \rangle _{Zh}= \frac{\sqrt{m_h^4-2 m_h^2 \left( 4 m_\psi ^2+m_Z^2\right) +\left( m_Z^2-4 m_\psi ^2\right) ^2}}{3072 \pi (g_{ZZh})^{-2} m_\psi ^4 m_Z^6} \nonumber \\&\quad \times \left[ \left\{ 3 {(g_{A\psi }^{Z})}^2 \left( m_h^4-2 m_h^2 \left( 4 m_\psi ^2 +m_Z^2\right) +\left( m_Z^2-4 m_\chi ^2\right) ^2\right) \right. \right. \nonumber \\&\quad \left. \left. +\,{(g_{V\psi }^{Z})}^2 3 m_Z^4 \left( -8 m_\psi ^2 \left( m_h^2-5 m_Z^2\right) \right. \right. \right. \nonumber \\&\quad \left. \left. \left. +\left( m_h^2-m_Z^2\right) ^2+16 m_\psi ^4\right) {\left( m_Z^2-4 m_\psi ^2\right) ^{-2}} \right\} \right. \nonumber \\&\quad \left. -\,\frac{v^2 \left( m_Z^2-4 m_\psi ^2\right) ^{-3}}{ \left( (m_h-m_Z)^2-4 m_\psi ^2\right) \left( (m_h+m_Z)^2-4 m_\psi ^2\right) }\right. \nonumber \\&\quad \left. \times \left[ {(g_{A\psi }^{Z})}^2 \left( m_Z^2-4 m_\psi ^2\right) \left\{ -96 m_\psi ^6 \left( 5 m_h^2+7 m_Z^2\right) \right. \right. \right. \nonumber \\&\quad \left. \left. \left. +\,5 m_Z^4 \left( m_h^2-m_Z^2\right) ^2+8 m_\psi ^4 \left( 12 m_h^4+6 m_h^2 m_Z^2+43 m_Z^4\right) \right. \right. \right. \nonumber \\&\quad \left. \left. \left. -\,2 m_\chi ^2 m_Z^2 \left( 24 m_h^4-37 m_h^2 m_Z^2+59 m_Z^4\right) +384 m_\psi ^8\right\} \right. \right. \nonumber \\&\quad \left. \left. \times \left( m_h^4-2 m_h^2 \left( 4 m_\psi ^2+m_Z^2\right) +\left( m_Z^2-4 m_\psi ^2\right) ^2\right) \right. \right. \nonumber \\&\quad \left. \left. +\,m_Z^4 {(g_{V\psi }^{Z})}^2 \left\{ 128 m_\psi ^8 \left( 37 m_h^2-82 m_Z^2\right) +5 m_Z^2 \left( m_h^2-m_Z^2\right) ^4\right. \right. \right. \nonumber \\&\quad \left. \left. \left. +\,32 m_\psi ^6 \left( -69 m_h^4+217 m_h^2 m_Z^2+242 m_Z^4\right) \right. \right. \right. \nonumber \\&\quad \left. \left. \left. +\,2 m_\psi ^2 \left( m_h^2-m_Z^2\right) ^2 \left( -16 m_h^4+m_h^2 m_Z^2+37 m_Z^4\right) \right. \right. \right. \nonumber \\&\quad \left. \left. \left. +\,8 m_\chi ^4 \left( 55 m_h^6-178 m_h^4 m_Z^2 \right. \right. \right. \right. \nonumber \\&\quad \left. \left. \left. \left. +\,147 m_h^2 m_Z^4-200 m_Z^6\right) -3584 m_\psi ^{10}\right\} \right] \right] . \end{aligned}$$

#### Appendix A.2.3: Vectorial dark matter

We use superscripts “A” and “NA” to represent Abelian and non-Abelian scenarios, respectively.

$$VV \rightarrow \overline{f} f$$:

A.25 $$\begin{aligned}&\langle \sigma v \rangle _{ff}^\mathrm{A}= (g_{VVZ'})^2\sum _f n_c^f \frac{{(g_{Af}^{Z'})}^2 m_f^2 v^2 \sqrt{m_V^2-m_f^2}}{9 \pi m_V m_{Z'}^4}\nonumber \\&\quad +\,\sum _f n_c^f \frac{5 (g_{VVZ'})^2 v^4}{324 \pi m_V m_{Z'}^4 \sqrt{m_V^2-m_f^2} \left( m_{Z'}^2-4 m_V^2\right) ^2} \nonumber \\&\quad \times \left[ {(g_{Af}^{Z'})}^2 \left\{ m_f^4 \left( 240 m_V^4-120 m_V^2 m_{Z'}^2+23 m_{Z'}^4\right) \right. \right. \nonumber \\&\quad \left. \left. -\,4 m_f^2 \left( 48 m_V^6-24 m_V^4 m_{Z'}^2+7 m_V^2 m_{Z'}^4\right) +8 m_V^4 m_{Z'}^4\right\} \right. \nonumber \\&\quad \left. -\,4 m_{Z'}^4 {(g_{Vf}^{Z'})}^2 \left( m_f^4+m_f^2 m_V^2-2 m_V^4\right) \right] . \end{aligned}$$

A.26 $$\begin{aligned}&\langle \sigma v \rangle _{ff}^\mathrm{NA}=(g_{VVZ'})^2 v^2 \sum _{f} n_c^f \frac{\sqrt{m_V^2-m_f^2}}{\pi m_V \left( m_{Z'}^2-4 m_V^2\right) ^2}\nonumber \\&\quad \times \left( 2{(g_{Af}^{Z'})}^2 \left( m_V^2- m_f^2\right) ^2+ {(g_{Vf}^{Z'})}^2 (2 m_V^2 + m_f^2)\right) .\nonumber \\ \end{aligned}$$

$$VV \rightarrow W^+ W^-$$:

A.27 $$\begin{aligned}&\langle \sigma v \rangle _{WW}^\mathrm{A}=(g_{VVZ'})^2 (g_{WWZ'})^2 v^4 \frac{5}{108 \pi m_W^4 (m_{Z'}^2-4 m_V^2)^2} \nonumber \\&\quad \times \, \sqrt{1-\frac{m_W^2}{m_V^2}}\left( 4 m_V^6-8 m_V^4 m_W^2-9 m_V^2 m_W^4-3 m_W^6\right) . \end{aligned}$$

A.28 $$\begin{aligned}&\langle \sigma v \rangle _{WW}^\mathrm{NA}=(g_{VVZ'})^2 (g_{WWZ'})^2 v^2 {\left( 1-\frac{m_W^2}{m_V^2}\right) }^{3/2} \nonumber \\&\quad \times \,\frac{m_V^2 \left( 3 m_W^4+20 m_W^2 m_V^2 +4 m_V^4\right) }{2 \pi m_W^4 \left( m_{Z'}^2-4 m_V^2\right) ^2}. \end{aligned}$$

$$VV \rightarrow Z' Z'$$:

A.29 $$\begin{aligned}&\langle \sigma v \rangle _{Z' Z'}^\mathrm{A}=(g_{VVZ'})^4 \nonumber \\&\quad \times \left( \frac{ \left( 32 m_V^8-56 m_V^6 m_{Z'}^2+69 m_V^4 m_{Z'}^4-50 m_V^2 m_{Z'}^6+14 m_{Z'}^8\right) }{144 \pi m_V^3 m_{Z'}^4 {(m^2_V-m^2_{Z'})}^{-{1/2}} \left( m_{Z'}^2-2 m_V^2\right) ^2}\right. \nonumber \\&\quad \left. +\,\frac{v^2 \left( 512 m_V^{14}-832 m_V^{12} m_{Z'}^2-952 m_V^{10} m_{Z'}^4+4292 m_V^8 m_{Z'}^6\right) }{1728 \pi m_V^3 \sqrt{(m^2_V-m^2_{Z'})} \left( m_{Z'}^3-2 m_V^2 m_{Z'}\right) ^4}\right. \nonumber \\&\quad \left. +\,\frac{v^2 \left( -5500 m_V^6 m_{Z'}^8+3391 m_V^4 m_{Z'}^{10} \right) }{1728 \pi m_V^3 \sqrt{(m^2_V-m^2_{Z'})} \left( m_{Z'}^3-2 m_V^2 m_{Z'}\right) ^4}\right. \nonumber \\&\quad \left. +\,\frac{v^2 \left( -994 m_V^2 m_{Z'}^{12}+110 m_{Z'}^{14}\right) }{1728 \pi m_V^3 \sqrt{(m^2_V-m^2_{Z'})} \left( m_{Z'}^3-2 m_V^2 m_{Z'}\right) ^4}\right) . \end{aligned}$$

A.30 $$\begin{aligned}&\langle \sigma v \rangle _{Z'Z'}^\mathrm{NA}=(g_{VVZ'})^4 \frac{\sqrt{(m^2_V-m^2_{Z'})}}{144 \pi m_V^7 m_{Z'}^4 \left( m_{Z'}^2-2 m_V^2\right) ^2}\nonumber \\&\quad \times \left( 144 m_V^{12}-272 m_V^{10} m_{Z'}^2 +316 m_V^8 m_{Z'}^4-264 m_V^6 m_{Z'}^6 \right. \nonumber \\&\quad \left. +\,168 m_V^4 m_{Z'}^8-14 m_V^2 m_{Z'}^{10}+3 m_{Z'}^{12}\right) \nonumber \\&\quad +\,\frac{ v^2 (g_{VVZ'})^4}{1728 \pi m_V^7 \sqrt{(m^2_V-m^2_{Z'})} \left( m_{Z'}^3-2 m_V^2 m_{Z'}\right) ^4}\nonumber \\&\quad \times \left( 3584 m_V^{18}-8256 m_V^{16} m_{Z'}^2+6208 m_V^{14} m_{Z'}^4 +41 m_{Z'}^{18} \right. \nonumber \\&\quad \left. -\,17456 m_V^{10} m_{Z'}^8+21372 m_V^8 m_{Z'}^{10}-10488 m_V^6 m_{Z'}^{12}\right. \nonumber \\&\quad \left. +\,2480 m_V^4 m_{Z'}^{14}-346 m_V^2 m_{Z'}^{16}+3104 m_V^{12} m_{Z'}^6\right) . \end{aligned}$$

$$VV \rightarrow Zh$$:

A.31 $$\begin{aligned} \langle \sigma v \rangle _\mathrm{Zh}^\mathrm{NA}= & {} (g_{ZZh})^2 v^2 \nonumber \\&\times \, \sqrt{ 4 m^2_V - {(m_h-m_Z)}^2} \sqrt{4 m^2_V-{(m_h+m_Z)}^2}\nonumber \\&\times \, \frac{\left( -2 m_Z^2 \left( m_h^2-20 m_V^2\right) +\left( m_h^2-4 m_V^2\right) ^2+m_Z^4\right) }{512 \pi m_V^4 \left( m_Z^3-4 m_V^2 m_Z\right) ^2}.\nonumber \\ \end{aligned}$$

### Appendix A.3: Pseudoscalar portal

A.32 $$\begin{aligned} \langle \sigma v \rangle _{ff}= & {} \sum _f \frac{n_c^f (g_{af})^2 (g_{\psi \psi a})^2 m_\psi \sqrt{m_\psi ^2-m_f^2}}{2 \pi \left( m_a^2-4 m_\psi ^2\right) ^2}. \end{aligned}$$

A.33 $$\begin{aligned} \langle \sigma v \rangle _{aa}= & {} \frac{1}{12 \pi }(g_{\psi \psi a})^4 \frac{m_\psi ^6}{{\left( m_a^2-2 m_\psi ^2\right) }^4}{\left( 1-\frac{m_a^2}{m_\psi ^2}\right) }^{5/2}. \end{aligned}$$

### Appendix A.4: Scalar + light pseudoscalar portal

The expressions for the cross-sections into $\overline{f} f$ and *SS* final states can be straightforwardly derived from the previous ones. We will, thus, just report the cross-sections relative to the *aa* and *Sa* final states. Contrary to the previous cases, this setup being more specific, we will stick to the same notations for the couplings as adopted in the main text.

The process $\overline{\psi }\psi \rightarrow aa$, with respect to the case of the pure pseudoscalar portal, receives an additional contribution from s-channel exchange of the scalar field *S*, so that:

A.34 $$\begin{aligned}&\langle \sigma v \rangle _{aa}=\frac{1}{128 \pi m_\psi ^2}\sqrt{1-\frac{m_a^2}{m_\psi ^2}}\nonumber \\&\quad \times \left( \frac{32}{3}\frac{g_{\psi }^4 m_\psi ^4 {\left( m_a^2-m_\psi ^2\right) }^2}{{\left( m_a^2 -2 m_\psi ^2\right) }^4}+\frac{4 \lambda g_\psi ^2 m_\psi ^2 m_S^2}{{\left( m_S^2- 4 m_\psi ^2\right) }^2}\right) v^2. \end{aligned}$$

A.35 $$\begin{aligned}&\langle \sigma v \rangle _{Sa}=\frac{g_\psi ^2 \sqrt{m_a^4-2 m_a^2 \left( 4 m_\psi ^2+m_S^2\right) +\left( m_S^2-4 m_\psi ^2\right) ^2}}{64 \pi m_\psi ^4} \nonumber \\&\quad \times \left[ \frac{2 \lambda m_\psi ^2 m_S^2}{\left( m_a^2-4 m_\psi ^2\right) ^2} +\frac{g_\psi ^2 \left( m_a^2+4 m_\psi ^2-m_S^2\right) ^2}{\left( m_a^2-4 m_\psi ^2+m_S^2\right) ^2} \right. \nonumber \\&\quad \left. +\,\frac{2 \sqrt{2} g_\psi \sqrt{\lambda } m_\psi m_S \left( m_a^2+4 m_\psi ^2-m_S^2\right) }{\left( m_a^2-4 m_\psi ^2\right) \left( m_a^2-4 m_\psi ^2+m_S^2\right) } \right] \nonumber \\&\quad +\, \frac{v^2}{2} \left[ \frac{g_\psi ^2 \sqrt{m_a^4-2 m_a^2 \left( 4 m_\psi ^2+m_S^2\right) +\left( m_S^2-4 m_\psi ^2\right) ^2}}{12 m_\psi ^4}\right. \nonumber \\&\quad \left. \times \left\{ \frac{g_\psi ^2 \left( m_a^8-8 m_a^6 \left( m_S^2-2 m_\psi ^2\right) +14 m_a^4 \left( m_S^2-4 m_\psi ^2\right) ^2 \right) }{\left( m_a^2-4 m_\psi ^2+m_S^2\right) ^4} \right. \right. \nonumber \\&\quad \left. \left. -\,\frac{g_\psi ^2 \left( 8 m_a^2 \left( m_S^2-4 m_\psi ^2\right) ^2 \left( 10 m_\psi ^2+m_S^2\right) -\left( m_S^2-4 m_\psi ^2\right) ^4\right) }{\left( m_a^2-4 m_\psi ^2+m_S^2\right) ^4}\right. \right. \nonumber \\&\quad \left. \left. +\,\frac{2 \sqrt{2} g_\psi \sqrt{\lambda } m_\psi m_S}{\left( m_a^2-4 m_\psi ^2\right) ^2 \left( m_a^2-4 m_\psi ^2+m_S^2\right) ^3} \right. \right. \nonumber \\&\quad \left. \left. \times \left( m_a^8+m_a^6 \left( 28 m_\psi ^2-3 m_S^2\right) \right. \right. \right. \nonumber \\&\quad \left. \left. \left. +\,m_a^4 \left( -240 m_\psi ^4+3 m_S^4+40 m_\psi ^2 m_S^2\right) \right. \right. \right. \nonumber \\&\quad \left. \left. \left. -m_a^2 \left( -320 m_\psi ^6+m_S^6+12 m_\psi ^2 m_S^4 +16 m_\psi ^4 m_S^2\right) \right. \right. \right. \nonumber \\&\quad \left. \left. \left. -\,8 m_\psi ^2 \left( m_S^2-4 m_\psi ^2\right) ^3\right) +\frac{6 \lambda m_\psi ^2 m_S^2 \left( m_a^2+4 m_\psi ^2\right) }{\left( m_a^2-4 m_\psi ^2\right) ^3}\right\} \right. \nonumber \\&\quad \left. -\,\frac{g_\psi ^2 \left( m_a^2-4 m_\psi ^2\right) ^{-2} }{4 m_\psi ^4 \left( m_a^2-4 m_\psi ^2+m_S^2\right) ^2}\right. \nonumber \\&\quad \left. \times \frac{ \left( -20 m_\psi ^2 \left( m_a^2+m_S^2\right) +3 \left( m_a^2-m_S^2\right) ^2+32 m_\psi ^4\right) }{\sqrt{m_a^4-2 m_a^2 \left( 4 m_\psi ^2+m_S^2\right) +\left( m_S^2-4 m_\psi ^2\right) ^2}}\right. \nonumber \\&\quad \left. \times \left( g_\psi ^2 \left( m_a^2-4 m_\psi ^2\right) ^2 \left( m_a^2+4 m_\psi ^2-m_S^2\right) ^2 \right. \right. \nonumber \\&\quad \left. \left. +\,2 \sqrt{2} g_\psi \sqrt{\lambda } m_\psi m_S \left( m_a^2-4 m_\psi ^2\right) \times \left( m_a^4-\left( m_S^2-4 m_\psi ^2\right) ^2\right) \right. \right. \nonumber \\&\quad \left. \left. +\,2 \lambda m_\psi ^2 m_S^2 \left( m_a^2-4 m_\psi ^2+m_S^2\right) ^2\right) \right] . \end{aligned}$$

### Appendix A.5: t-channel portals

As already mentioned in the main text that in the case of $\mathscr {O}(1)$ coupling, the DM relic density is mainly accounted by the DM pair annihilations into the SM fermion pairs. We will, thus, explicitly report only the cross-sections corresponding to these processes.

#### Appendix A.5.1: Complex scalar dark matter

A.36 $$\begin{aligned} \langle \sigma v \rangle _{ff}v= & {} \sum _f n^c_f \frac{{(\lambda _{\varPsi _u})}^4 ~m_f^2 \left( m_\chi ^2-m_f^2\right) ^{3/2}}{16 \pi m_\chi ^3 \left( -m_f^2+m_\chi ^2+m_{\varPsi _u}^2\right) ^2} \nonumber \\&+\,\sum _f n^c_f \frac{{(\lambda _{\varPsi _u})}^4 \sqrt{m_\chi ^2-m_f^2} v^2}{192 \pi m_\chi ^3 \left( -m_f^2+m_\chi ^2+m_{\varPsi _u}^2\right) ^4} \nonumber \\&\times \left[ 15 m_f^8-6 m_f^6 \left( 9 m_\chi ^2+5 m_{\varPsi _u}^2\right) \right. \nonumber \\&\left. +\,m_f^4 \left( m_\chi ^2 +m_{\varPsi _u}^2\right) \left( 71 m_\chi ^2+15 m_{\varPsi _u}^2\right) \right. \nonumber \\&\left. -\,8 m_f^2 m_\chi ^2 \left( 5 m_\chi ^4+9 m_\chi ^2 m_{\varPsi _u}^2+m_{\varPsi _u}^4\right) \right. \nonumber \\&\left. +\,8 m_\chi ^4 \left( m_\chi ^2+m_{\varPsi _u}^2\right) ^2\right] . \end{aligned}$$

#### Appendix A.5.2: Dirac fermion dark matter

A.37 $$\begin{aligned}&\langle \sigma v \rangle _{ff}=\sum _f n^c_f \frac{{(\lambda _{\Sigma _u})}^4 ~m_\psi \sqrt{m_\psi ^2-m_f^2}}{32 \pi \left( -m_f^2+m_\Sigma ^2+m_\psi ^2\right) ^2}\nonumber \\&\quad +\,\sum _f n^c_f\frac{{(\lambda _{\Sigma _u})}^4 ~v^2}{384 \pi m_\psi \sqrt{m_\psi ^2-m_f^2} \left( -m_f^2+m_{\Sigma _u}^2+m_\psi ^2\right) ^4}\nonumber \\&\quad \times \left[ 3 m_\psi ^2 \left( 5 m_f^2-4 m_\psi ^2\right) \left( -m_f^2+m_{\Sigma _u}^2+m_\psi ^2\right) ^2 \right. \nonumber \\&\quad \left. -\,2 \left( m_\psi ^2-m_f^2\right) \left( m_f^2-m_{\Sigma _u}^2+m_\psi ^2\right) \right. \nonumber \\&\quad \left. \times \left( m_f^4-m_f^2 \left( m_{\Sigma _u}^2+2 m_\psi ^2\right) +7 m_{\Sigma _u}^2 m_\psi ^2+m_\psi ^4\right) \right] .\nonumber \\ \end{aligned}$$

#### Appendix A.5.3: Majorana fermion dark matter

A.38 $$\begin{aligned} \langle \sigma v \rangle _{ff}= & {} \sum _f n^c_f \frac{{(\lambda _{\Sigma _u})}^4 ~m_f^2 \sqrt{m_\psi ^2-m_f^2}}{8 \pi m_\psi \left( -m_f^2+m_{\Sigma _u}^2+m_\psi ^2\right) ^2} \nonumber \\&+\, \sum _f n^c_f \frac{{(\lambda _{\Sigma _u})}^4 ~m_\psi v^2}{96 \pi \sqrt{m_\psi ^2-m_f^2} \left( -m_f^2+m_{\Sigma _u}^2+m_\psi ^2\right) ^4}\nonumber \\&\times \left[ -42 m_f^2 m_\psi ^6+13 m_f^4 \left( m_f^2-m_{\Sigma _u}^2\right) ^2 \right. \nonumber \\&\left. +\,m_\psi ^4 \left( 49 m_f^4-44 m_f^2 m_{\Sigma _u}^2+16 m_{\Sigma _u}^4\right) +16 m_\psi ^8 \right. \nonumber \\&\left. -\,2 m_f^2 m_\psi ^2 \left( 18 m_f^4-35 m_f^2 m_{\Sigma _u}^2+13 m_{\Sigma _u}^4\right) \right] .\nonumber \\ \end{aligned}$$

## Footnotes

1 See Ref. [22] for an exception (“relentless” DM) for modified expansion histories.

2 We make also use of: $\frac{ds}{dt}=\left[ \frac{3s}{T}\left( 1+\frac{T}{3h_\mathrm{eff}}\frac{dh_\mathrm{eff}}{dT}\right) \right] \frac{dT}{dt}$.                   (8)

3 In scenarios where the DM is not the only new particle state, other processes like co-annihilations, might also be relevant for the DM relic density. A more general definition of $\langle \sigma v \rangle$, including such processes, can be found e.g., in Ref. [24].

4 The velocity distribution is understood as the probability of finding a WIMP with velocity *v* at a time *t*.

5 See Refs. [106–114] for other competitive limits.

6 There are still some exceptions to this direct relation between non-velocity dependent annihilation cross-section and relic density as discussed in detail in Ref. [25].

7 In the model considered in this work the mediator fields are mostly coupled with the SM fermions. The discussion can be straightforwardly extended to the case of sizable couplings with the gauge and Higgs bosons.

8 We will briefly consider a single case of spin-1/2 mediator. We won’t consider higher spin assignations. For these cases the interested reader can look for example at Refs. [164, 167].

9 We will also consider the case of a pseudoscalar mediator. As will be clarified later, this is a more specific scenario since, under the main assumptions considered in this work, it can occur only for a fermionic DM. We refer to the dedicated section later for the corresponding Lagrangian.

10 As can be easily guessed that the DM annihilations also yield *WW*, *ZZ* , *Zh* and *hh* as possible final states.

11 An analogous study has been performed in Ref. [172]. Our results are in substantial agreement with the ones reported in this reference.

12 We limit, for simplicity, to the lowest dimensional operators. Higher dimensional operators are discussed, for example, in Ref. [180].

13 We will report in the main text just the results of the analysis. Analytical expressions of the relevant rates are extensively reported in the Appendix.

14 In the mentioned figure and throughout all the paper we will, as convention, use the label “PLANCK”, as the corresponding experiment, for iso-contours corresponding to the correct DM relic density.

15 Similar to the case of the Higgs portal we just quote, as an example, the lowest dimensional operator. This is however, not the only possible option.

16 Similar to the SM-Higgs portal case we will report in the main text only the main results while discussing the computation in more detail in the Appendix.

17 This statement is strictly valid in the case, considered here, of $\lambda _\chi ^S=1$. In the case $\lambda _\chi ^S \ll \lambda _S$ the contribution of the trilinear couplings $\lambda _S$ to the annihilation cross-section into *SS* might be sizable and event dominant. However, we expect in such a case, since the annihilation cross-section would scale at least as $(\lambda _\chi ^{S})^2$, the DM to be in general overabundant.

18 Interestingly, couplings of the $Z^{\prime }$ with SM particles as presented here are similar to the ones in several existing EW extensions of the SM which can be embedded in different GUT models [257–260].

19 The difference arises from the fact that in the case of spin-0 mediator the quantities $f_p$ and $f_n$ are originated by matrix element $\langle N| q q |N\rangle$ which is related to the mass of the nucleon. In the case of spin-1 mediator one instead evaluates the matrix element $\langle N|q \gamma ^\mu q |N\rangle$ which is, instead, related to the electric charge of the nucleon.

20 Given the non-trivial gauge charges, the t-channel mediators can also couple with the gluons, the photon and the *Z*-boson. We do not explicitly write such interactions for simplicity. In the case of the scalar mediator a renormalizable 4-field coupling with the SM-Higgs doublet, i.e., $H^\dagger H \Sigma ^\dagger _q \Sigma _q$, can arise at the tree-level. We will assume that the corresponding coupling is negligible.

21 In Eq. (63) the labels *u* and *d* refer globally to up- and down-type quarks. The couplings and the masses of the mediator fields carry also a generation index which is not explicitly reported (see main text for further clarification).

22 By relaxing this hypothesis it is possible to have a viable decaying DM candidate model with characteristic phenomenology [275–277].

23 One could weaken the constraints by taking smaller values of the associated couplings. However, the relic density would then be achieved only in the very fine-tuned co-annihilation regime.

24 For this reason it is also dubbed as coy DM [294].

25 Values of $m_a$ below 10 GeV are still partially in tension with low energy constraints for $c_a=1$. We will ignore this issue since it has marginal impact on our discussion.

26 For the mass range assumed for the DM, the ID signal probed by dSph relies on the $\overline{b} b$ final state for $m_\psi < m_t$ and on the $\overline{t} t$ final state for $m_\psi > m_t$. For simplicity we have adopted everywhere the limit from the $\overline{b}b$ final state. This is a good enough approximation for the purposes of our study.

27 A scenario in which $m_\psi$ and $g_\psi$ are related by a dynamical mechanism has recently been discussed in Refs. [299, 300].

28 For simplicity, we have considered in our analysis only limits from searches in dSphs. As pointed out in Refs. [299, 302, 303] the annihilation into *Sa* can lead to box shaped gamma-ray signals which could be probed in the near future by CTA. However, our choice for $m_a$, implies a too suppressed decay branching ratio for $a\rightarrow \gamma \gamma$ process. For this reason we have not considered explicitly this possible signal in our analysis.

29 Stability of a scalar DM is protected either by a *U*(1) (complex scalar) or by a ${\mathbb {Z}}_2$ symmetry (real scalar) and thus, the associated Lagrangian contains ${|\chi |}^2$ or $\chi ^2$ term, respectively. In this analysis we have used the notation ${|\chi |}^2$ for generality although the DM is considered to be a real scalar.

30 The DM as a gauge boson of the dark sector connected to the SM through the SM-Higgs sector has also been proposed, for different DM production mechanisms, in Refs. [310, 311].

31 We have adopted the same definition of the covariant derivative as of Ref. [312].

32 In the case of a scalar DM it would even be possible to generate a “blind spot” in the scattering cross-section for a rather specific choice of the couplings $\lambda ^\chi _{H,\varPhi }$ [221, 313] which would induce a destructive interference between the amplitudes associated to the *h* and *S* exchange. A different solution for relaxing DD constraints in the presence of multiple scalar singlets coupled to the SM-Higgs has recently been proposed in Ref. [314].

33 Note that in the definition of $g_X$ we have also encoded the value of *U*(1) charge of the DM.

34 The case of an Abelian vectorial DM in the kinetic mixing scenario will be addressed in a dedicated future publication.

35 In the case of extreme mass degeneracy between the $H^0$ and $A^0$ states, one should consider an additional SI interaction, namely $H^0 p \rightarrow A^0 p$, mediated by the *Z* boson [327]. We will neglect this possibility implicitly by assuming a mass splitting of at least 1 GeV between these two states. This, in turn, implies that the coupling $\lambda _5$ is always not negligible.

36 There are elegant ways to overcome this underabundant DM regime in the two Higgs Doublet Model using axions [343].

37 SI interactions are also induced by interactions of the DM with the SM Higgs boson. Their contributions are sub-dominant and for simplicity we will omit them in our discussion. It has nevertheless been included in our numerical analysis.

38 The maximal value of the DM mass is actually higher because of Sommerfeld enhancement [352–354]. For simplicity we have neglected this effect since it would marginally influence the discussion.

39 Figure 27 reports only limits from SI interactions. For the chosen assignation of the parameters, limits from SD interactions are always sub-dominant. For simplicity, thus, these have not been reported in the figure.
